# Clinical Outcomes and Cost-Effectiveness of Collaborative Dementia Care: A Secondary Analysis of a Cluster Randomized Clinical Trial

**DOI:** 10.1001/jamanetworkopen.2024.19282

**Published:** 2024-07-05

**Authors:** Bernhard Michalowsky, Iris Blotenberg, Moritz Platen, Stefan Teipel, Ingo Kilimann, Elena Portacolone, Jens Bohlken, Anika Rädke, Maresa Buchholz, Annelie Scharf, Franka Muehlichen, Feng Xie, Jochen René Thyrian, Wolfgang Hoffmann

**Affiliations:** 1 German Center for Neurodegenerative Diseases (DZNE), Rostock/Greifswald, Greifswald, Germany; 2 German Center for Neurodegenerative Diseases (DZNE), Rostock/Greifswald, Rostock, Germany; 3 Department of Psychiatry and Psychotherapy, University Medicine Greifswald, Greifswald, Germany; 4 Institute for Health & Aging, University of California, San Francisco; 5 Philip R. Lee Institute for Health Policy Studies, University of California, San Francisco; 6 Institute of Social Medicine, Occupational Health and Public Health, Faculty of Medicine, University of Leipzig, Leipzig, Germany; 7 Department of Health Research Methods, Evidence, and Impact, McMaster University, Hamilton, Canada; 8 Program for Health Economics and Outcome Measures, Hamilton, Canada; 9 Institute for Community Medicine, Section Epidemiology of Health Care and Community Health, University Medicine Greifswald, Greifswald, Germany

## Abstract

**Importance:**

Long-term evidence for the effectiveness and cost-effectiveness of collaborative dementia care management (CDCM) is lacking.

**Objective:**

To evaluate whether 6 months of CDCM is associated with improved patient clinical outcomes and caregiver burden and is cost-effective compared with usual care over 36 months.

**Design, Setting, and Participants:**

This was a prespecified secondary analysis of a general practitioner (GP)–based, cluster randomized, 2-arm clinical trial conducted in Germany from January 1, 2012, to December 31, 2014, with follow-up until March 31, 2018. Participants were aged 70 years or older, lived at home, and screened positive for dementia. Data were analyzed from March 2011 to March 2018.

**Intervention:**

The intervention group received CDCM, comprising a comprehensive needs assessment and individualized interventions by nurses specifically qualified for dementia care collaborating with GPs and health care stakeholders over 6 months. The control group received usual care.

**Main Outcomes and Measures:**

Main outcomes were neuropsychiatric symptoms (Neuropsychiatric Inventory [NPI]), caregiver burden (Berlin Inventory of Caregivers’ Burden in Dementia [BIZA-D]), health-related quality of life (HRQOL, measured by the Quality of Life in Alzheimer Disease scale and 12-Item Short-Form Health Survey [SF-12]), antidementia drug treatment, potentially inappropriate medication, and cost-effectiveness (incremental cost per quality-adjusted life year [QALY]) over 36 months. Outcomes between groups were compared using multivariate regression models adjusted for baseline scores.

**Results:**

A total of 308 patients, of whom 221 (71.8%) received CDCM (mean [SD] age, 80.1 [5.3] years; 142 [64.3%] women) and 87 (28.2%) received usual care (mean [SD] age, 79.2 [4.5] years; 50 [57.5%] women), were included in the clinical effectiveness analyses, and 428 (303 [70.8%] CDCM, 125 [29.2%] usual care) were included in the cost-effectiveness analysis (which included 120 patients who had died). Participants receiving CDCM showed significantly fewer behavioral and psychological symptoms (adjusted mean difference [AMD] in NPI score, −10.26 [95% CI, −16.95 to −3.58]; *P* = .003; Cohen *d*, −0.78 [95% CI, −1.09 to −0.46]), better mental health (AMD in SF-12 Mental Component Summary score, 2.26 [95% CI, 0.31-4.21]; *P* = .02; Cohen *d*, 0.26 [95% CI, −0.11 to 0.51]), and lower caregiver burden (AMD in BIZA-D score, −0.59 [95% CI, −0.81 to −0.37]; *P* < .001; Cohen *d*, −0.71 [95% CI, −1.03 to −0.40]). There was no difference between the CDCM group and usual care group in use of antidementia drugs (adjusted odds ratio, 1.91 [95% CI, 0.96-3.77]; *P* = .07; Cramér *V*, 0.12) after 36 months. There was no association with overall HRQOL, physical health, or use of potentially inappropriate medication. The CDCM group gained QALYs (0.137 [95% CI, 0.000 to 0.274]; *P* = .049; Cohen *d*, 0.20 [95% CI, −0.09 to 0.40]) but had no significant increase in costs (437€ [−5438€ to 6313€] [US $476 (95% CI, −$5927 to $6881)]; *P* = .87; Cohen *d*, 0.07 [95% CI, −0.14 to 0.28]), resulting in a cost-effectiveness ratio of 3186€ (US $3472) per QALY. Cost-effectiveness was significantly better for patients living alone (CDCM dominated, with lower costs and more QALYs gained) than for those living with a caregiver (47 538€ [US $51 816] per QALY).

**Conclusions and Relevance:**

In this secondary analysis of a cluster randomized clinical trial, CDCM was associated with improved patient, caregiver, and health system–relevant outcomes over 36 months beyond the intervention period. Therefore, it should become a health policy priority to initiate translation of CDCM into routine care.

**Trial Registration:**

ClinicalTrials.gov Identifier: NCT01401582

## Key Points

**Question:**

Is collaborative dementia care management (CDCM) clinically effective and cost-effective over 36 months compared with usual care?

**Findings:**

In this secondary analysis of a cluster randomized clinical trial of 308 patients with dementia, those receiving CDCM had significantly fewer behavioral and psychological symptoms and better mental health compared with those receiving usual care over 36 months. CDCM was associated with reduced caregiver burden and likely to have been cost-effective, especially for patients living alone.

**Meaning:**

The findings suggest that CDCM is associated with improved patient, caregiver, and health system–relevant outcomes over 36 months and that its translation into routine care should become a health policy priority.

## Teaser

This secondary analysis of a cluster randomized clinical trial investigates clinical outcomes and the cost-effectiveness of collaborative dementia care management compared with usual care among adults with dementia who live at home.

## Introduction

Health care systems worldwide are experiencing resource constraints, which could cause poor adherence to recommended treatments.^1^ This is especially the case for the increasing number of people living with dementia, who have high multimorbidity in addition to cognitive impairment^2^ and require comprehensive multiprofessional support.^3,4^

General practitioners (GPs) are responsible for identifying the first signs of cognitive decline and initiating dementia-specific diagnostics and treatment.^5^ However, primary care systems are unprepared to manage this complex postdiagnostic support.^6^ Studies have revealed that dementia often remains undetected,^7^ with patients not receiving evidence-based treatment in primary care^8,9^; this may result in unaddressed health care needs, which are associated with poor health outcomes and caregiver burden.^10,11,12^ Measures aimed at attenuating and alleviating symptom progression can enhance health-related quality of life (HRQOL), prolong autonomy in daily life for a person living with dementia, and conserve health care resources.^13,14^

Collaborative dementia care management (CDCM) programs are defined as interventions delivered in the community to coordinate postdiagnostic, multiprofessional support in primary care for people living with dementia and their caregivers, considering their individual needs according to evidence-based guidelines.^3,15,16^ A meta-analysis across 13 randomized clinical trials conducted until 2011 revealed that CDCM was associated with delayed institutionalization, reduced behavioral and psychological symptoms over 18 months, and improved caregiver burden over 6 months.^17^ Subsequent randomized clinical trials showed a significant increase in antidementia drug prescriptions and improved behavioral and psychological symptoms, caregiver burden and HRQOL over 12 months, improvements in quality of care, reduction in caregiver burden, and decreased health care utilization and costs over 18 months.^18,19,20,21^

All trials were limited to short observational periods. Evidence from methodologically rigorous studies over an extended time horizon (>24 months) is lacking. Studies also yielded heterogeneous cost-effective and cost-ineffective results over 12 and 24 months.^22,23,24^ Promising results of delayed institutionalization over 12 and 18 months^18,19,20,21^ could offset the cost-effectiveness if more institutionalizations occur later, but this is currently unknown. Therefore, the objective of this study was to evaluate, for the first time to our knowledge, the association of CDCM with patient, caregiver, and health system–relevant outcomes and the cost-effectiveness of CDCM compared with usual care over 36 months.

## Methods

### Trial Design

This was a secondary analysis based on 36-month follow-up data from the DelpHi-MV GP-based cluster randomized clinical trials (NCT01401582), which was designed to test the efficacy of a CDCM in primary dementia care.^25^ The trial protocol (Supplement 1), eligibility criteria, sample size calculation, baseline characteristics, efficacy after 12 months, and cost-effectiveness after 24 months are described elsewhere.^24,25,26,27,28,29^ Reporting followed the Consolidated Standards of Reporting Trials (CONSORT) ^30,31^ and Consolidated Health Economic Evaluation Reporting Standards (CHEERS)^32^ reporting guidelines. The trial was approved by the ethical committee of the Chamber of Physicians of Mecklenburg-Western Pomerania, Germany. Written informed consent was obtained. Physicians received incentives for patient screening (10€ [US $11]) and inclusion (100€ [US $109]).

For the trial, GP practices (n = 854) in Mecklenburg Western-Pomerania, a federal state in Germany, were invited to participate. A total of 136 GP practices that agreed to participate were randomized (1:1) to the intervention (CDCM) or the control group (usual care) by the study center using simple randomization without stratification or matching.

The GP practices were not informed about their randomization status but became aware of the randomization due to the nature of the intervention, likely causing a reduced recruitment motivation in practices in the control group and, thus, an imbalanced group distribution. The same nurses performed data assessment and interventions to reduce the burden for the participants with dementia. Therefore, blinding was not possible. Physicians checked patients’ eligibility (age ≥70 years, community dwelling, and screened positive for dementia^33^), informed them about the study, and asked for written informed consent.

### Sample, Participant Flow, and Dropout

Study enrollment started on January 1, 2012, and ended on December 31, 2014. The 36-month follow-up was completed on March 31, 2018. Participants were included if they completed at least 2 of 3 annual assessments. Patients who died were included in the cost-effectiveness analyses. The trial flowchart and the results of dropout analyses are given in eFigure 1 and eTables 1 and 2 in Supplement 2.

### CDCM Intervention

The CDCM intervention was developed according to dementia-specific guidelines,^34,35,36,37^ targeting the individual participant level, and was delivered in participants’ homes by specifically qualified nurses for 6 months, aiming to support participants living with dementia and their caregivers through coordination and management of individualized optimal treatment and care within the health and social care system.^26,27^ The main intervention pillars were (1) management of individualized treatment and care, (2) medication management, and (3) caregiver support. After a standardized, comprehensive assessment of patients’ and caregivers’ unmet needs, the nurses generated an individualized intervention task list, discussed these tasks in an interdisciplinary case conference (nursing scientist, neurologist or psychiatrist, psychologist, and pharmacist) and with the treating GP, and carried out tasks in cooperation with the GP and various health and social care practitioners over 6 months. The medication management system^38^ generated recommendations for GPs concerning antidementia drugs, drug-related problems, interactions, and adverse events. The nurses monitored the task completion in 6 home visits with an average duration of 1 hour. Intervention feedback from GPs was documented, indicating high agreement.^39^

The intervention was supported by an information technology– and algorithm-based intervention management system, enabling swift systematic identification of needs and implementation and monitoring of intervention tasks. The qualification, intervention, and intervention management system are explained elsewhere.^26,27,28,38,40^ The intervention implementation costs are described in eTable 3 in Supplement 2. Participants of the control group received care as usual.

### Outcomes

Nurses conducted assessments at baseline and at 12, 24, and 36 months. Assessments consisted of standardized, computer-assisted, face-to-face interviews at the participant’s or caregiver’s home.

#### Clinical Outcomes

Primary study outcomes included the following assessments. Behavioral and psychological symptoms were measured by the Neuropsychiatric Inventory (NPI; score range 1-144, with higher scores indicating greater severity and frequency of neuropsychiatric symptoms, a proxy rating given by caregivers on 12 dimensions of neuropsychiatric behaviors among people living with dementia).^41^ Caregiver burden was measured by the Berlin Inventory of Caregivers’ Burden in Dementia (BIZA-D; score range 0-4, with higher scores indicating greater caregiver burden), with the subdomain “individual limitation and health” summarizing lack of energy, perceived physical and mental burden of informal care, and limitations in the realization of one’s own needs.^42^ Health-related QOL was measured by the Quality of Life in Alzheimer Disease scale (QOL-AD; score range 1-4, with higher scores indicating better QOL), a disease-specific measure of HRQOL with 13 dimensions,^43^ and by the 12-Item Short-Form Health Survey (SF-12; score range 0-100, with higher scores indicating better health),^44^ a generic, multidimensional instrument assessing physical (Physical Component Summary [PCS]) and mental (Mental Component Summary [MCS]) health. Contrary to the trial protocol, the SF-12 was used to assess the association with mental and physical health separately. Prescription of antidementia drugs (donepezil, galantamine, rivastigmine, and memantine) and potentially inappropriate medications according to the PRISCUS criteria^45^ was also assessed.

Secondary outcomes were cognitive impairment according to the Mini-Mental State Examination (score range 0-30, with higher scores indicating better cognition),^46^ depression according to the 15-item Geriatric Depression Scale (score range 0-15, with higher scores indicating worse depression),^47^ and functional impairment according to the Bayer Activities of Daily Living Scale (B-ADL; score range, 0-10, with higher scores indicating worse functional status).^48^

#### Cost-Effectiveness Outcomes

The cost-effectiveness analysis was based on a preference-based scoring algorithm converting SF-12 responses to health utilities (Short Form 6-Dimension algorithm [SF-6D]) anchored at 0 (death) and 1 (full health) to calculate quality-adjusted life-years (QALYs). The analysis also considered health care utilization using the Resource Utilization in Dementia questionnaire,^49^ completed by caregivers and health services practitioners, and medical records (eTable 4 in Supplement 2).

### Statistical Analysis

This secondary analysis deviated from the trial protocol by extending the time horizon to 36 months.^25^ Data were analyzed from March 2011 to March 2018 using Stata, version 16 (StataCorp LLC). Two-sided *P* < .05 was considered significant.

In addition to clinical variables, demographics (age, sex, and living situation), comorbidities (number of diagnoses listed in GP files, Charlson Comorbidity Index), and the number of drugs regularly taken were used for sample description and model adjustments. Study group differences at baseline were assessed using generalized linear and logistic models with random effects for clusters (GP practices). Missing data were handled using multiple imputations by chained equations (eAppendix 1 in Supplement 2).^50,51^

#### Clinical Outcome Analyses

Descriptive statistics and *t* tests were used to describe and compare outcomes between groups. Calculated effect sizes (Cohen *d*, Cramér *V*) between groups were classified as small (Cohen *d* = 0.2; Cramér *V* = 0.1), medium (Cohen *d* = 0.5; Cramér *V* = 0.3), or large (Cohen *d* = 0.8; Cramér *V* = 0.5).^52^ We performed multivariable linear regression models for 12-, 24-, and 36-month associations with the outcome variable (nonstandardized and z-standardized) as a dependent variable and the study group as the variable of interest. The outcome baseline value was included as a covariate to account for interindividual variance and to reduce residual variance. Age, sex, and living situation (alone vs not alone) were included as covariates. Due to a declining number of patients per cluster with increasing study duration, random effects were only considered when the intraclass correlation for the respective outcome was higher than 0.3 (eAppendixes 2 and 3 in Supplement 2).^53^

#### Cost-Effectiveness Analysis

Costs were calculated from a public payer perspective in 2023 values in euros ($1.08 = €1) based on health care utilization and unit costs^54,55^ (eTable 3 in Supplement 2). The health utilities and day of death were used for QALY calculation (eAppendix 4 in Supplement 2). In cases of death, from the day of death, health utilities and costs were set to 0. Quality-adjusted life-years and costs were discounted at 3.5% per annum.

The incremental cost-effectiveness ratio (ICER) was calculated using the incremental cost per QALY gained by the CDCM compared with usual care.^56^ Due to baseline differences in functional impairment, which is associated with costs,^57^ incremental costs and QALYs were estimated using linear regression models adjusted for age, sex, living situation, and functional impairment.^58,59^ To handle sampling uncertainty in the ICER, nonparametric bootstrapping with 1000 resamples stratified for group distribution was used.^60^

#### Sensitivity Analyses

Sensitivity analyses were used to test the robustness of results. These included complete case analysis, truncating cost outliers (99th percentile) to minimize the impact of patients with high costs, and subgroup analysis for people living with dementia who were living alone at home vs not living alone for exploratory purposes, since caregiver availability could affect the CDCM cost-effectiveness.

## Results

### Patient Characteristics

A total of 6838 people were screened at 128 GP practices; 1166 fulfilled the eligibility criteria, 634 agreed to participate, and 516 started the baseline assessment. A total of 174 participants withdrew their informed consent, 139 died, and 10 suspended the assessments until the 3-year follow-up.

A total of 308 participants, of whom 221 (71.8%) received CDCM (mean [SD] age, 80.1 [5.3] years; 79 [35.7%] men; 142 [64.3%] women) and 87 (28.2%) received usual care (mean [SD] age, 79.2 [4.5] years; 37 [42.5%] men; 50 [57.5%] women), were included in the clinical outcomes analyses. An additional 120 participants who died were included in the cost-effectiveness analyses, extending the sample to 428 people with dementia (303 [70.8%] receiving CDCM and 125 [29.2%], usual care). The participants’ characteristics are summarized in Table 1. There were no significant differences in baseline characteristics between groups except for functional impairment (mean [SD] B-ADL score, 3.6 [2.5] for CDCM vs 2.7 [1.8] for controls; *P* = .006). Dementia severity tended to progress more slowly in the usual care group, resulting in fewer moderate and more mild cases than in the intervention group after 36 months (eTable 11 in Supplement 2).

**Table 1.** Baseline Participant Characteristics of the Sample for the Clinical Outcome Analysis Available After 3 Years

Characteristic	Participants^a^		
	CDCM (n = 221)	Usual care (n = 87)	*P* value^b^
Demographics			
Age, mean (SD), y	80.1 (5.3)	79.2 (4.5)	.15
Sex			
Female	142 (64.3)	50 (57.5)	.30
Male	79 (35.7)	37 (42.5)	
Caregiver included	159 (71.9)	54 (62.0)	.11
Living alone	115 (52.0)	39 (44.8)	.31
Clinical characteristics			
Cognitive status			
Mean (SD), MMSE score^c^	22.2 (5.4)	22.9 (5.7)	.32
Positive screening result not confirmed	50 (22.6)	28 (32.2)	.28
Mild dementia	115 (52.1)	42 (48.3)	
Moderate dementia	50 (22.6)	14 (16.1)	
Severe dementia	6 (2.7)	3 (3.5)	
Depression, mean (SD), GDS-15 score^d^	3.1 (2.4)	2.7 (1.8)	.18
Functional impairment, mean (SD), BADL score^e^	3.6 (2.5)	2.7 (1.8)	.006
*ICD-10* diagnoses, mean (SD), No.^f^	13.9 (8.1)	14.2 (6.8)	.78
Charlson Comorbidity Index score^g^			
Mean (SD)	3.5 (2.2)	3.3 (2.3)	.52
No score	6 (2.7)	4 (4.6)	.48
Low	35 (15.8)	19 (21.8	
High	81 (36.7)	29 (33.3	
Very high	99 (44.8)	35 (40.2	
Drugs taken, mean (SD), No.^h^	6.5 (3.2)	6.4 (2.7)	.31

Abbreviations: BADL, Bayer Activities of Daily Living Scale; CDCM, collaborative dementia care management; GDS-15, Geriatric Depression Scale–15; *ICD-10*, *International Statistical Classification of Diseases and Related Health Problems, Tenth Revision*; MMSE, Mini-Mental State Examination.

^a^ Data are presented as number (percentage) of participants unless otherwise indicated.

^b^ Based on generalized linear models (metric variables) or logistic regression models (categorical variables) with random intercepts for the general practitioner (metric variables), representing the cluster.

^c^ MMSE score range, 0 to 30, with higher scores indicating better cognition.

^d^ GDS-15 score range, 0 to 15, with higher scores indicating worse depression.

^e^ BADL score range, 0 to 10, with higher scores indicating more functional deficits.

^f^ Based on the number of *ICD-10* diagnoses recorded in the medical record of the treating general practitioner.

^g^ Low was indicated by 0 to 2 points; high, 3 to 4 points; and very high, more than 4 points.

^h^ Drugs needing to be taken regularly, based on cabinet review at patients’ homes.

### Clinical Outcomes

Compared with people living with dementia receiving usual care, those receiving CDCM for 6 months had fewer behavioral and psychological symptoms at 12 months (adjusted mean difference in NPI scores, −7.66 [95% CI, −11.38 to −3.94]; *P* < .001), 24 months (−8.55 [95% CI, −13.58 to −3.53]; *P* = .001), and 36 months (−10.26 [95% CI, −16.95 to −3.58]; *P* = .003), characterized by medium effect sizes (Cohen *d*, −0.52 [95% CI, −0.83 to −0.21] at 12, −0.52 [95% CI, −0.84 to −0.21] at 24, and −0.78 [95% CI, −1.09 to −0.46] at 36 months). There was a decrease in caregiver burden at 36 months (adjusted mean difference in BIZA-D scores, −0.59 [95% CI, −0.81 to −0.37]; *P* < .001), with a moderate effect size (Cohen *d*, −0.71 [95% CI, −1.03 to −0.40]), but there was no difference at 12 months (−0.18 [95% CI, −0.39 to 0.02]; *P* = .08) or 24 months (−0.06 [95% CI, −0.31 to 0.18]; *P* = .60).

There was no association of CDCM with overall HRQOL (QOL-AD, SF-6D) and physical health (SF-12 PCS). However, mental health was significantly better in the intervention group at 24 (adjusted mean difference in SF-12 MCS scores, 2.37 [95% CI, 0.31-4.42]; *P* = .02) and 36 (2.26 [95% CI, 0.31-4.21]; *P* = .02) months, with small effect sizes (Cohen *d*, 0.26 [95% CI, −0.01 to 0.51] and 0.26 [95% CI, −0.11 to 0.51], respectively), but not at 12 months (adjusted mean difference, 1.72 [95% CI, −0.24 to 3.68]; *P* = .09; Cohen *d*, 0.18 [95% CI, −0.07 to 0.43]).

**Figure 1.**
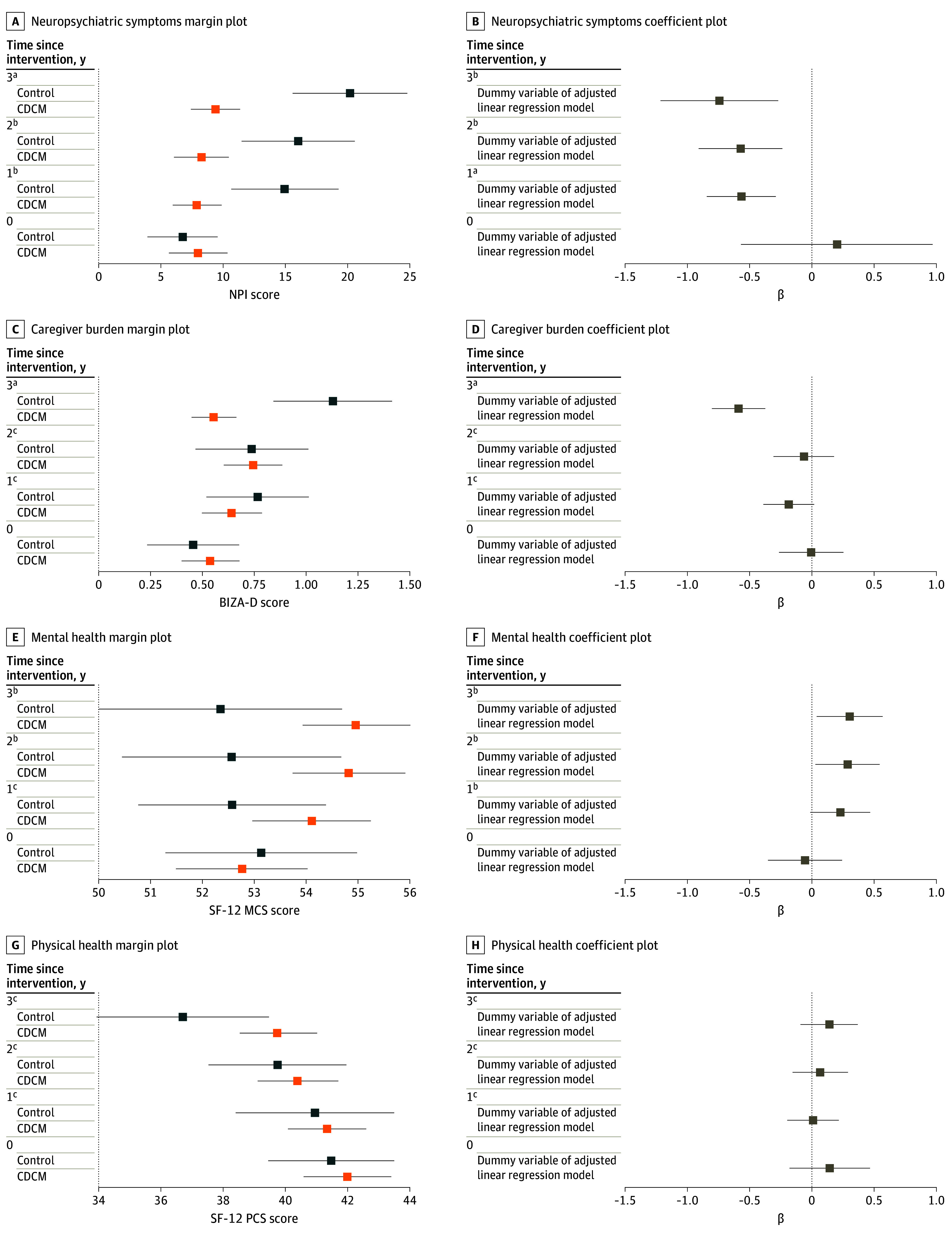
Margin and Coefficients Plots Demonstrating the Treatment Effects of Collaborative Dementia Care Management (CDCM) vs Usual Care BIZA-D indicates Berlin Inventory of Caregivers’ Burden With Dementia Patients questionnaire; NPI, Neuropsychiatric Inventory; SF-12, 12-Item Short-Form Health Survey. Error bars indicate 95% CIs. ^a^*P* < .001. ^b^*P* < .05. ^c^*P* < .01.

People living with dementia receiving CDCM had significantly higher odds of taking antidementia drugs after 12 months (adjusted odds ratio [AOR], 2.56 [95% CI, 1.18-5.55]; *P* = .02) and 24 months (AOR, 3.06 [95% CI, 1.39-6.75]; *P* = .006) but not at 36 months (AOR, 1.91 [95% CI, 0.96-3.77]; *P* = .07), with small effect sizes (Cramér *V*, 0.14 at 12 months, 0.16 at 24 months, and 0.12 at 36 months). There was no association of CDCM with use of inappropriate drugs. Clinical effectiveness results are shown in Table 2, Figure 1, and eTables 5 to 7 in Supplement 2. Complete case analyses showed similar results. Secondary outcome findings (worsening cognition and depression at 24 and 36 months in the CDCM group) are shown in eTable 7 in Supplement 2.

**Table 2.** Comparison of Primary Outcomes Between Collaborative Model of Dementia Care and Usual Care Over Time

Metric	Score, mean (SE)		Difference, mean (95% CI)		Cohen *d* (95% CI)^c^
	CDCM	Usual care	Unadjusted^a^	Adjusted^b^	
**Behavioral and psychological symptoms of dementia, NPI^d^**					
Baseline	7.9 (1.2)	6.7 (1.4)	1.23 (−3.12 to 5.59)	2.59 (−7.39 to 12.58)	0.09 (−0.22 to 0.40)
Year 1	7.9 (1.0)	15.0 (2.1)	−7.07 (−11.27 to −2.87)^e^	−7.66 (−11.38 to −3.94)^e^	−0.52 (−0.83 to −0.21)
Year 2	8.3 (1.1)	16.0 (2.2)	−7.78 (−12.39 to −3.17)^e^	−8.55 (−13.58 to −3.53)^e^	−0.52 (−0.84 to −0.21)
Year 3	9.4 (1.0)	20.2 (2.3)	−10.81 (−15.12 to −6.50)^e^	−10.26 (−16.95 to −3.58)^f^	−0.78 (−1.09 to −0.46)
**Caregiver burden, BIZA-D^d^**					
Baseline	0.54 (0.07)	0.45 (0.11)	0.08 (−0.19 to 0.36)	−0.01 (−0.27 to 0.26)	0.09 (−0.21 to 0.41)
Year 1	0.64 (0.07)	0.77 (0.12)	−0.12 (−0.41 to 0.16)	−0.18 (−0.39 to 0.02)^g^	−0.13 (−0.44 to 0.17)
Year 2	0.74 (0.07)	0.74 (0.14)	0.00 (−0.28 to 0.29)	−0.06 (−0.31 to 0.18)	−0.01 (−0.30 to 0.32)
Year 3	0.56 (0.05)	1.13 (0.14)	−0.57 (−0.82 to −0.32)^e^	−0.59 (−0.81 to −0.37)^e^	−0.71 (−1.03 to −0.40)
**Quality of life, QOL-AD^d^**					
Baseline	2.77 (0.03)	2.84 (0.04)	−0.07 (−0.02 to 0.16)	−0.07 (−0.16 to 0.02)	−0.19 (−0.44 to 0.05)
Year 1	2.78 (0.02)	2.80 (0.04)	−0.02 (−0.06 to 0.10)	0.03 (−0.04 to 0.09)	−0.06 (−0.31 to 0.19)
Year 2	2.70 (0.03)	2.68 (0.04)	0.02 (−0.11 to 0.07)	0.06 (−0.02 to 0.14)	0.05 (−0.19 to 0.30)
Year 3	2.71 (0.02)	2.69 (0.03)	0.02 (−0.10 to 0.07)	0.06 (−0.02 to 0.13)	0.05 (−0.19 to 0.30)
**Mental health, SF-12 MCS^d^**					
Baseline	52.8 (0.64)	53.1 (0.93)	−0.38 (−2.70 to 1.95)	−0.23 (−2.55 to 2.08)	−0.04 (−0.29 to 0.21)
Year 1	54.1 (0.58)	52.6 (0.91)	1.53 (−0.62 to 3.68)	1.72 (−0.24 to 3.68)^g^	0.18 (−0.07 to 0.43)
Year 2	54.7 (0.55)	52.4 (1.06)	2.27 (0.09 to 4.44)^h^	2.37 (0.31 to 4.42)^h^	0.26 (−0.01 to 0.51)
Year 3	54.7 (0.50)	52.6 (0.98)	2.09 (0.09 to 4.09)^h^	2.26 (0.31 to 4.21)^h^	0.26 (−0.11 to 0.51)
**Physical health, SF-12 PCS^d^**					
Baseline	41.9 (0.71)	41.5 (1.01)	0.52 (−2.04 to 3.09)	0.86 (−1.71 to 3.42)	0.05 (−0.20 to 0.30)
Year 1	41.4 (0.64)	40.9 (1.28)	0.39 (−2.17 to 2.95)	0.17 (−1.80 to 2.15)	0.04 (−0.21 to 0.29)
Year 2	40.5 (0.66)	39.8 (1.11)	0.67 (−1.81 to 3.14)	0.58 (−1.48 to 2.64)	0.07 (−0.18 to 0.32)
Year 3	38.9 (0.57)	37.7 (1.16)	1.24 (−1.04 to 3.52)	1.14 (−0.78 to 3.06)	0.14 (−0.11 to 0.38)
**Health utility, SF-6D^d^**					
Baseline	0.77 (0.01)	0.75 (0.01)	0.01 (−0.02 to 0.05)	0.02 (−0.02 to 0.05)	0.09 (−0.16 to 0.34)
Year 1	0.78 (0.01)	0.75 (0.01)	0.02 (−0.01 to 0.05)	0.02 (−0.01 to 0.04)	0.14 (−0.11 to 0.39)
Year 2	0.76 (0.01)	0.74 (0.01)	0.02 (−0.01 to 0.06)	0.02 (−0.01 to 0.05)	0.15 (−0.09 to 0.40)
Year 3	0.75 (0.01)	0.73 (0.01)	0.02 (−0.02 to 0.05)	0.02 (−0.02 to 0.05)	0.09 (−0.12 to 0.37)
**Medication use**	**No. (%) (n = 221)**	**No. (%) (n = 87)**	**Difference, No. (%)**	**Adjusted OR (95% CI)**	**Cramér *V***
**Antidementia drug^i^**					
Baseline	58 (26.2)	18 (20.7)	48 (5.5)	1.49 (0.81 to 2.73)	0.06
Year 1	84 (38.0)	20 (23.0)	64 (15.0)^h^	2.56 (1.18 to 5.55)^f^	0.14
Year 2	86 (38.9)	19 (21.8)	67 (17.1)^f^	3.06 (1.39 to 6.75)^f^	0.16
Year 3	86 (38.9)	23 (26.4)	63 (12.5)^h^	1.91 (0.96 to 3.77)^g^	0.12
**Potentially inappropriate medication^j^**					
Baseline	57 (25.8)	16 (18.4)	41 (7.4)	1.58 (0.84 to 2.95)	0.07
Year 1	56 (25.3)	12 (13.8)	44 (11.5)^h^	1.94 (0.90 to 4.17)^g^	0.12
Year 2	49 (22.2)	13 (14.9)	36 (7.3)	1.37 (0.66 to 2.87)	0.08
Year 3	46 (20.8)	10 (11.5)	36 (9.3)^g^	1.76 (0.80 to 3.85)	0.11

Abbreviations: CDCM, collaborative dementia care management; BIZA-D, Berlin Inventory of Caregivers’ Burden With Dementia Patients questionnaire; NPI, Neuropsychiatric Inventory; OR, odds ratio; QOL-AD, Quality of Life in Alzheimer Disease scale; SF-6D, Short-Form 6-Dimension algorithm; SF-12 MCS, 12-Item Short-Form Mental Component Summary; SF-12 PCS, SF-12 Physical Component Summary.

^a^ A description of scores is given in the Methods section.

^b^ *t* Tests were calculated.

^c^ Multivariate regressions adjusted for baseline score, age, sex, and living situation.

^d^ Based on unadjusted mean difference.

^e^ *P* < .001. ^f^*P* < .01. ^g^*P* < .10. ^h^*P* < .05.

^i^ Donepezil, rivastigmine, galantamine, memantine, and donepezil and memantine.

^j^ According to the PRISCUS list.

### Cost-Effectiveness

**Figure 2.**
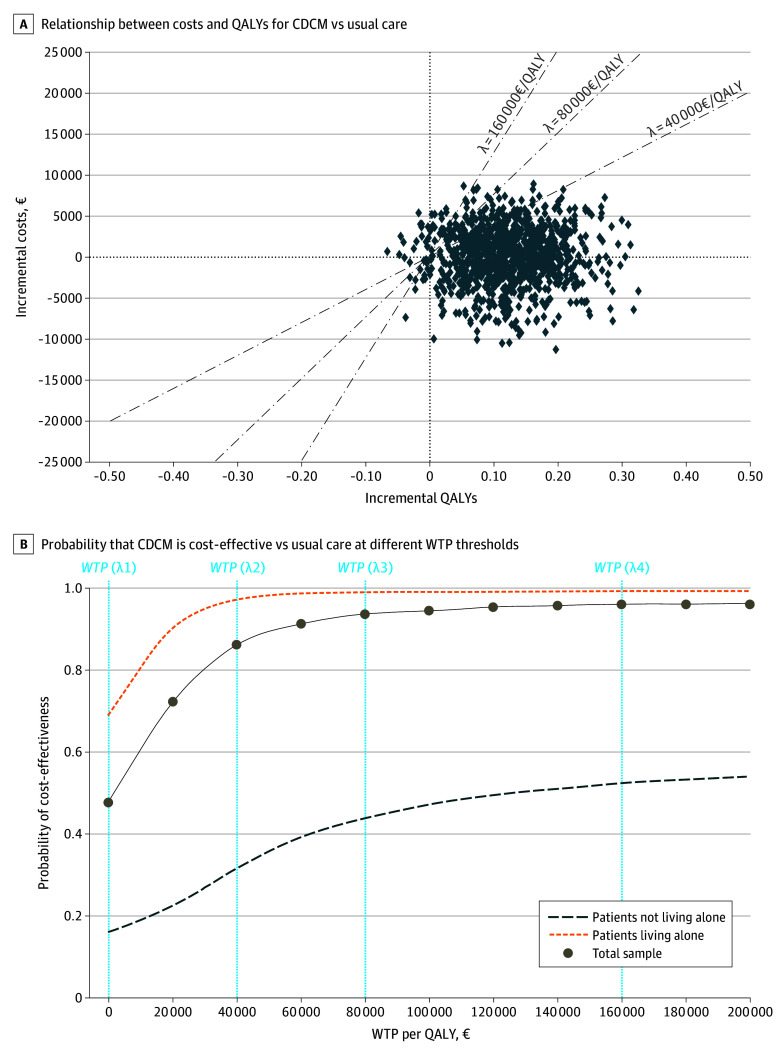
Cost-Effectiveness Plane and Acceptability Curves for Collaborative Dementia Care Management (CDCM) vs Usual Care A, Estimates are based on regression analyses of incremental costs and effects within 1000 bootstrap sample replications of the initial sample stratified for intervention and control group. Each point represents, for several resamples, the incremental cost and the incremental quality-adjusted life-years (QALYs) of the intervention compared with usual care, demonstrating whether the intervention was more effective and less costly (lower right quadrant), more effective and more costly (upper right quadrant), less effective and less costly (lower left quadrant), or less effective and more costly (upper left quadrant). B, Curves indicate the likelihood that the intervention is cost-effective at the given threshold if society has a willingness to pay (WTP) a certain amount per QALY. Vertical dashed lines (WTP [λ] values) indicate WTP thresholds of 40 000€, 80 000€, and 160 000€ per QALY. To convert euros to US dollars, multiply by 1.09.

Total costs after 36 months were 31 396€ (95% CI, 28 234€-34 558€) (US $34 222 [95% CI, $30 775-$37 668]) for the CDCM group and 30 959€ (95% CI, 26 024€-35 894€) (US $33 745 [95% CI, $28 366-$39 124]) for the usual care group. The CDCM intervention was associated with significantly increased medication and medical aid costs but with lower costs in all other cost categories, especially for hospitalizations. There was no association with institutionalization after 36 months. Adjusted mean difference in incremental QALYs (0.137 [95% CI, 0.000-0.274]; *P* = .049; Cohen *d*, 0.20 [95% CI, −0.09 to 0.40]) and cost (437€ [95% CI, −5438€ to 6313€] [US $476 (95% CI, −$5927 to $6881)]; *P* = .87; Cohen *d*, 0.07 [95% CI, −0.14 to 0.28]) resulted in an ICER of 3186€ (US $3472) per QALY, indicating that CDCM gained QALYs by higher costs (Table 3 and eTables 8 and 9 in Supplement 2). The probability of the CDCM being cost-effective was 87% and 94% at a willingness to pay (WTP) of 40 000€ (US $43 600) and 80 000€ (US $87 200) per QALY gained, respectively (Figure 2).

**Table 3.** Unadjusted and Adjusted Mean Cost and Differences and Incremental Cost-Effectiveness Ratio

Outcome	Mean (SE) [95% CI]^a^					
	Unadjusted			Adjusted		
	CDCM	Usual care	Difference	CDCM	Usual care	Difference
Health care cost						
Total	31 355 (1527) [28 354 to 34 356]	29 797 (3109) [23 686 to 35 909]	1558 (3104) [−4542 to 7659]	30 876 (1609) [27 714 to 34 038]	30 959 (2511) [26 024 to 35 894]	−82 (2989) [−5958 to 5793]
Medical treatments						
All	17 277 (887) [15 534 to 19 021]	17 686 (2413) [12 944 to 22 429]	−409 (2075) [−4487 to 3669]	17 211 (1125) [15 001 to 19 421]	17 849 (1755) [14 398 to 21 297]	−637 (2089) [−4744 to 3470]
Physician	972 (34) [906 to 1039]	1094 (68) [960 to 1228]	−122 (68) [−256 to 12]^b^	982 (36) [911 to 1053]	1070 (56) [959 to 1181]	−87 (67) [−220 to 44]
In-hospital	7400 (742) [5942 to 8858]	9186 (2333) [4600 to 13 772]	−1786 (1890) [−5502 to 1929]	7380 (1024) [5367 to 9394]	9234 (1599) [6092 to 12 377]	−1853 (1903) [−5595 to 1888]
Medications	4972 (239) [4503 to 5441]	4036 (299) [3448 to 4624]	937 (418) [114 to 1759]^c^	4968 (227) [4522 to 5414]	4048 (354) [3351 to 4744]	919 (421) [91 to 1749]^c^
Medical aids	3495 (144) [3216 to 3778]	2903 (186) [2538 to 3268]	592 (254) [92 to 1091]^c^	3451 (132) [3192 to 3709]	3011 (206) [2607 to 3415]	439 (244) [−42 to 920]^b^
Therapies	438 (59) [321 to 554]	466 (106) [258 to 675]	−29 (115) [−254 to 197]	430 (59) [315 to 545]	485 (91) [305 to 665]	−54 (108) [−269 to 159]
Formal care						
All	14 078 (1004) [12 103 to 16 052]	12 111 (1727) [8716 to 15 506]	1967 (1917) [−1802 to 5735]	13 665 (951) [11 796 to 15 535]	13 111 (1484) [1651 to 3335]	54 (1766) [−2919 to 4027]
Day and night care	2419 (276) [1877 to 2961]	2290 (497) [1313 to 3266]	129 (535) [−922 to 1181]	2335 (275) [1796 to 2875]	2493 (428) [1651 to 3335]	−157 (510) [−1160 to 845]
Ambulatory care	6121 (509) [5121 to 7122]	5688 (940) [3840 to 7535]	434 (996) [−1524 to 2391]	5888 (495) [4916 to 6860]	6253 (772) [4736 to 7770]	−364 (918) [−2170 to 1441]
Nursing home	5537 (831) [3904 to 7170]	4134 (1260) [1656 to 6611]	1404 (1526) [−1596 to 4403]	5442 (817) [3835 to 7048]	4365 (1275) [1859 to 6872]	1076 (1518) [−1908 to 4061]
Cost for CDCM intervention, €^d^	520 (0)	0 (0)	520 (0)	520 (0)	0 (0)	520 (0)
Total cost including intervention cost, €^d^	31 875 (1527) [28 871 to 34 880]	29 797 (3109) [23 686 to 35 909]	2078 (3104) [−4022 to 8179]	31 396 (1609) [28 234 to 34 558]	30 959 (2511) [26 024 to 35 894]	437 (2989) [−5438 to 6313]
QALYs	1.880 (0.04) [1.802 to 1.958]	1.794 (0.06) [1.666 to 1.922]	0.085 (0.07) [−0.061 to 0.232]	1.895 (0.04) [1.821 to 1.969]	1.758 (0.06) [1.643 to 1.873]	0.137 (0.07) [0.000 to 0.274]^c^
Incremental cost per QALY gained, €^d^	NA	NA	24 323	NA	NA	3186

Abbreviations: CDCM, collaborative dementia care management; NA, not applicable; QALY, quality-adjusted life-year.

^a^ For statistical comparison between groups, *t* tests (unadjusted means) and multivariate regression models adjusted for age, sex, and functional impairment at baseline (adjusted means) were calculated.

^b^ *P* < .10.

^c^ *P* < .05.

^d^ To convert to US dollars, multiply by 1.09.

Sensitivity analyses confirmed findings, demonstrating that CDCM was primarily cost-effective for people with dementia living alone (47 538€ [US $51 816] per QALY) (eTable 10 and eFigures 2 and 3 in Supplement 2). The gain in QALYs (living alone: 0.22 [95% CI, 0.03-0.42]; *P* = .03; not living alone: 0.08 [95% CI, −0.11 to 0.27]; *P* = .42), cost savings (living alone: −3295€ [95% CI, −12 573€ to 5982€] [US −$3592 (95% CI, −$13 705 to $6520)]; *P* = .49; not living alone: 3711€ [95% CI, −3514€ to 11 121€] [US $4045 (95% CI, −$3830 to $12 122)]; *P* = .31), and cost-effectiveness probability at WTP of 40 000€ (US $43 600) per QALY was higher in people with dementia who were living alone (92%, vs 32% in those not living alone). The observed trend of increased medication and medical aid costs and decreased hospitalization costs was solely evident for people with dementia who were living alone.

## Discussion

This study adds evidence for the effectiveness and cost-effectiveness of a 6-month collaborative care intervention for people living with dementia, demonstrating that CDCM was positively associated with behavioral and psychological symptoms, mental health, and caregiver burden over 36 months. Also, CDCM was likely to have been cost-effective, especially for people with dementia living alone.

Addressing behavioral and psychological symptoms in dementia is a clinical priority, but symptom management and alleviation are challenging. A meta-analysis by Reilly et al^17^ demonstrated that CDCM was associated with significantly reduced symptoms at 18 months (standardized mean difference, −0.35 [95% CI, −0.63 to −0.07]). We found that CDCM was consistently and clinically meaningfully^41^ positively associated with behavioral and psychological symptoms at 12, 24, and 36 months with moderate effect sizes, extending the current evidence. While symptoms steadily deteriorated in control individuals, the CDCM intervention of tailoring nonpharmacologic and pharmacologic approaches to the individual’s specific needs stabilized behavioral and psychological symptoms beyond the intervention period.^61,62^

Another goal of CDCM is to stabilize caregiver burden. Evidence from 2 studies suggests that CDCM can minimize caregiver burden with small effect sizes at 6 and 12 months.^20,63^ In another study, effects at 18 months or after were uncertain.^17^ We found a significantly lower caregiver burden after 12 and 36 months with moderate effect sizes. The absence of midterm differences at 24 months in previous studies^17,20,63^ and the minor differences in our study could be attributed to the intervention duration, usually 6 months or less. Addressing existing unmet needs during the intervention and carrying out interventions that better prepare people living with dementia and their caregivers in advance could have an immediate and lasting impact when the disease progresses. There was a trend for delayed institutionalization in the first and second year, followed by more frequent institutionalization in the third year in the intervention group, predominately for those living alone, which aligns with previous studies.^17^ Studies have revealed a decrease in caregiver burden immediately after institutionalization of people living with dementia,^64,65^ potentially explaining the large effect sizes in the current study at 36 months in the caregiver burden scale and behavioral and psychological symptoms. Both are caregiver ratings incorporating the caregiver burden. However, whether CDCM leads to better preparation for institutionalization should be investigated to support the community-dwelling living situation as long as possible and make institutionalizations as smooth as possible.

Pertaining to patients’ HRQOL, studies of CDCM outcomes have reported better HRQOL over 12 and 18 months.^20,66,67^ Our results align with these findings. However, other studies did not find effects on HRQOL.^19,68^ In this study, CDCM was associated with stabilized mental health over 36 months, whereas mental health continuously deteriorated in control individuals. Previous studies^19,68^ used HRQOL measures that summarized different domains into one without finding any effect. Assessing physical and mental health separately enables a differentiated view of the CDCM’s efficacy for HRQOL.

Concerning the economic outcomes, pooled data from 2 studies^69,70^ demonstrated that CDCM significantly reduced costs at 12 months. Preliminary data from the D-CARE study demonstrated that CDCM reduced hospitalization, delayed institutionalization, and lowered costs over 18 months.^21,71^ Our results align with these studies, indicating cost savings after 1 and 2 years attributable to lower hospitalization and institutionalization, and higher costs in the third year attributable to delayed institutionalizations. Our results also align with a meta-analysis of studies reporting higher care costs,^17^ as CDCM aims to increase the utilization of necessary care services. In terms of cost-effectiveness, previous studies provided inconclusive evidence.^18,23^ Our analysis revealed that CDCM was likely a cost-effective strategy compared with current WTP thresholds in high-income countries ($5480-$95 958 per QALY), especially for individuals living alone.^72^

### Limitations

This study has limitations. The generalizability of the results was limited mainly to patients with mild cognitive impairment in a primarily rural German setting. The data validity might be limited in terms of accuracy due to cognitive limitations of individuals with dementia. The group distribution was imbalanced, with fewer controls and a trend toward slower cognitive decline. To minimize patient burden, the same nurses collected data and intervened. These aspects may have biased results and conclusions. The identification of people living with dementia was based on a screening rather than a state-of-the-art diagnostic procedure. This could have led to false-positive inclusions. However, when equally distributed over groups, any false-positive inclusions would have led to an underestimation of intervention effects. Also, alternative statistical analyses, such as panel data regression, could have been used to better account for the longitudinal nature of the data, potentially providing more precise estimates. However, we aligned our approach with the trial’s statistical analysis plan (Supplement 1). Finally, sample size calculation was done for primary outcomes only, limiting the generalizability of secondary and economic outcome conclusions.

## Conclusions

In this secondary analysis of a cluster randomized clinical trial of CDCM for patients with dementia, CDCM was associated with improved patient, caregiver, and health-system–relevant outcomes over 36 months, suggesting that the intervention should be implemented into routine care, especially for people with dementia living alone. However, full implementation is challenging and would require health care reforms. While younger patients and patients with mild cognitive impairment have been more frequently diagnosed with dementia in recent years in primary care, which is an important aspect of disease-modifying treatments,^73^ there is still potential for increasing dementia diagnostics in older patients, demonstrating an implementation prerequisite for CDCM. Therefore, further research is needed to monitor primary dementia care. As a result, it is imperative that policymakers are informed about the evidence, starting government-led initiatives to support CDCM evidence transfer into health policies and care practice.

## Notes

Supplement 1.
Trial Protocol and Statistical Analysis Plan

Supplement 2. **eTable 1.** Drop-Out Analysis for Patients Excluded From Outcome and Cost-Effectiveness Analysis **eTable 2.** Drop-Out Analysis for Patients Who Dropped Out After Completing the Baseline Assessment Before Follow-Up at Years 1, 2, and 3 **eTable 3. **Cost Calculation for the Implementation of 1 Nurse Carrying Out the Collaborative Dementia Care Management for 90 Patients per Year **eTable 4.** Cost Categories and Unit Costs for Monetary Valuation of Medical and Formal Health Care Resources and Services **eTable 5. **Regression Analyses for the Treatment Effect of CDCM vs Usual Care **eTable 6. **Regression Analyses for the Treatment Effect of CDCM vs Usual Care Separately Over Years for Complete Cases **eTable 7. **Regression Analyses for the Treatment Effect of CDCM vs Usual Care Separately Over 1, 2, and 3 Years **eTable 8. **Adjusted Incremental Cost and Effects of CDCM vs Usual Care for Year 1, 2, 3, and Cumulative **eTable 9. **Unadjusted Health Care Resource Utilization, Cost, and Quality-Adjusted Life of CDCM vs Usual Care for Year 1, 2, 3, and Cumulative **eTable 10. **Adjusted Incremental Cost and Effects on QALYs of the Collaborative Model of Dementia Care vs Usual Care for Year 1, 2, 3, and Cumulative, Separately for Those Living Alone and Not Living Alone **eTable 11. **Comparison of the Participant Characteristics at Baseline and 3 Years After Baseline **eFigure 1. **Flow Chart of the DelpHi-MV Trial **eFigure 2. **Cost-Effectiveness Acceptability Curves of the Scenario Analyses **eFigure 3. **Cost-Effectiveness Plane and Cost-Effectiveness Acceptability Curves of the CDCM vs Usual Care After 3 Years, Shown Separately for Patients Living Alone and Not Living Alone **eAppendix 1. **Methods Used to Handle Missing Data and That Used Stata Code **eAppendix 2. **Intraclass Correlation and Design Effects for Each Outcome and Time Point **eAppendix 3. **Standardized Measure of Effect Size for Each Outcome and Time Point **eAppendix 4. **Calculation of Utility Values and Quality-Adjusted Life-Years

Supplement 3.
Data Sharing Statement

## References

[1] Plsek PE, Greenhalgh T. Complexity science: the challenge of complexity in health care. BMJ. 2001;323(7313):625-628. doi:10.1136/bmj.323.7313.625 11557716 PMC1121189

[2] Clague F, Mercer SW, McLean G, Reynish E, Guthrie B. Comorbidity and polypharmacy in people with dementia: insights from a large, population-based cross-sectional analysis of primary care data. Age Ageing. 2017;46(1):33-39. 28181629 10.1093/ageing/afw176

[3] Prince MCHA, Knapp M, Guerchet M, Karagiannidou M. World Alzheimer Report 2016: improving healthcare for people living with dementia—coverage, quality and costs now and in the future. Alzheimer’s Disease International. 2016. Accessed October 17, 2023. https://www.alzint.org/u/WorldAlzheimerReport2016.pdf

[4] 2020 Alzheimer’s disease facts and figures. Alzheimers Dement. Published online March 10, 2020. doi:10.1002/alz.12068 32157811

[5] Spenceley SM, Sedgwick N, Keenan J. Dementia care in the context of primary care reform: an integrative review. Aging Ment Health. 2015;19(2):107-120. doi:10.1080/13607863.2014.920301 24901364

[6] Johnston D, Samus QM, Morrison A, . Identification of community-residing individuals with dementia and their unmet needs for care. Int J Geriatr Psychiatry. 2011;26(3):292-298. doi:10.1002/gps.2527 20658473 PMC3039061

[7] Eichler T, Thyrian JR, Hertel J, . Rates of formal diagnosis in people screened positive for dementia in primary care: results of the DelpHi-Trial. J Alzheimers Dis. 2014;42(2):451-458. doi:10.3233/JAD-140354 24898640

[8] Platen M, Fless S, Rädke A, . Prevalence of low-value care and its associations with patient-centered outcomes in dementia. J Alzheimers Dis. 2021;83(4):1775-1787. doi:10.3233/JAD-210439 34459396 PMC8609693

[9] Wucherer D, Eichler T, Kilimann I, . Antidementia drug treatment in people screened positive for dementia in primary care. J Alzheimers Dis. 2015;44(3):1015-1021. doi:10.3233/JAD-142064 25391382

[10] Black BS, Johnston D, Rabins PV, Morrison A, Lyketsos C, Samus QM. Unmet needs of community-residing persons with dementia and their informal caregivers: findings from the maximizing independence at home study. J Am Geriatr Soc. 2013;61(12):2087-2095. doi:10.1111/jgs.12549 24479141 PMC4001885

[11] Eichler T, Thyrian JR, Hertel J, . Unmet needs of community-dwelling primary care patients with dementia in Germany: prevalence and correlates. J Alzheimers Dis. 2016;51(3):847-855. doi:10.3233/JAD-150935 26890767

[12] Platen M, Flessa S, Teipel S, . Impact of low-value medications on quality of life, hospitalization and costs—a longitudinal analysis of patients living with dementia. Alzheimers Dement. 2023;19(10):4520-4531. doi:10.1002/alz.13012 36905286

[13] Black BS, Rabins P. Quality of life in dementia: conceptual and practical issues. In: Ames D, Burns A, O’Brien J, eds. *Dementia*. 4th ed. CRC Press; 2010:293-304.

[14] Robinson L, Tang E, Taylor JP. Dementia: timely diagnosis and early intervention. BMJ. 2015;350:h3029. doi:10.1136/bmj.h3029 26079686 PMC4468575

[15] D’Souza MF, Davagnino J, Hastings SN, Sloane R, Kamholz B, Twersky J. Preliminary data from the Caring for Older Adults and Caregivers at Home (COACH) Program: a care coordination program for home-based dementia care and caregiver support in a Veterans Affairs medical center. J Am Geriatr Soc. 2015;63(6):1203-1208. doi:10.1111/jgs.13448 26032224

[16] Somme D, Trouve H, Dramé M, Gagnon D, Couturier Y, Saint-Jean O. Analysis of case management programs for patients with dementia: a systematic review. Alzheimers Dement. 2012;8(5):426-436. doi:10.1016/j.jalz.2011.06.004 22285637

[17] Reilly S, Miranda-Castillo C, Malouf R, . Case management approaches to home support for people with dementia. Cochrane Database Syst Rev. 2015;1(1):CD008345. doi:10.1002/14651858.CD008345.pub2 25560977 PMC6823260

[18] MacNeil Vroomen J, Bosmans JE, van de Ven PM, . Community-dwelling patients with dementia and their informal caregivers with and without case management: 2-year outcomes of a pragmatic trial. J Am Med Dir Assoc. 2015;16(9):800.e1-800.e8. doi:10.1016/j.jamda.2015.06.011 26170035

[19] Thyrian JR, Hertel J, Wucherer D, . Effectiveness and safety of dementia care management in primary care: a randomized clinical trial. JAMA Psychiatry. 2017;74(10):996-1004. doi:10.1001/jamapsychiatry.2017.2124 28746708 PMC5710469

[20] Possin KL, Merrilees JJ, Dulaney S, . Effect of collaborative dementia care via telephone and internet on quality of life, caregiver well-being, and health care use: the care ecosystem randomized clinical trial. JAMA Intern Med. 2019;179(12):1658-1667. doi:10.1001/jamainternmed.2019.4101 31566651 PMC6777227

[21] Reuben D, Panlilio M; UCLA Alzheimer’s and Dementia Care Program. Effects of an Alzheimer’s and dementia care co-management program on quality, clinical outcomes, and utilization. Alzheimers Dement. 2022;18(S9):e063939. doi:10.1002/alz.063939

[22] Guterman EL, Kiekhofer RE, Wood AJ, . Care ecosystem collaborative model and health care costs in Medicare beneficiaries with dementia: a secondary analysis of a randomized clinical trial. JAMA Intern Med. 2023;183(11):1222-1228. doi:10.1001/jamainternmed.2023.4764 37721734 PMC10507595

[23] Meeuwsen E, Melis R, van der Aa G, et al. Cost-effectiveness of one year dementia follow-up care by memory clinics or general practitioners: economic evaluation of a randomised controlled trial. *PLoS One*. 2013;8(11):e79797. doi:10.1371/journal.pone.0079797 PMC3839971 24282511

[24] Michalowsky B, Xie F, Eichler T, . Cost-effectiveness of a collaborative dementia care management—results of a cluster-randomized controlled trial. Alzheimers Dement. 2019;15(10):1296-1308. doi:10.1016/j.jalz.2019.05.008 31409541

[25] Thyrian JR, Fib T, Dreier A, . Life- and person-centred help in Mecklenburg-Western Pomerania, Germany (DelpHi): study protocol for a randomised controlled trial. Trials. 2012;13:56. doi:10.1186/1745-6215-13-56 22575023 PMC3482148

[26] Dreier A, Thyrian JR, Eichler T, Hoffmann W. Qualifications for nurses for the care of patients with dementia and support to their caregivers: a pilot evaluation of the dementia care management curriculum. Nurse Educ Today. 2016;36:310-317. doi:10.1016/j.nedt.2015.07.024 26277428

[27] Eichler T, Thyrian JR, Dreier A, . Dementia care management: going new ways in ambulant dementia care within a GP-based randomized controlled intervention trial. Int Psychogeriatr. 2014;26(2):247-256. doi:10.1017/S1041610213001786 24152974 PMC3891295

[28] Mühlichen F, Michalowsky B, Rädke A, . Tasks and activities of an effective collaborative dementia care management program in German primary care. J Alzheimers Dis. 2022;87(4):1615-1625. doi:10.3233/JAD-215656 35491783 PMC9277686

[29] Thyrian JR, Eichler T, Michalowsky B, . Community-dwelling people screened positive for dementia in primary care: a comprehensive, multivariate descriptive analysis using data from the DelpHi-Study. J Alzheimers Dis. 2016;52(2):609-617. doi:10.3233/JAD-151076 27031481

[30] Campbell MK, Piaggio G, Elbourne DR, Altman DG; CONSORT Group. CONSORT 2010 statement: extension to cluster randomised trials. BMJ. 2012;345:e5661. doi:10.1136/bmj.e5661 22951546

[31] Antes G. The new CONSORT statement. BMJ. 2010;340:c1432. doi:10.1136/bmj.c1432 20332507

[32] Fayanju OM, Haukoos JS, Tseng JF. CHEERS reporting guidelines for economic evaluations. JAMA Surg. 2021;156(7):677-678. doi:10.1001/jamasurg.2021.0540 33825848 PMC8282685

[33] Calabrese P, Kessler J. Screening for cognitive impairment in dementia—the DemTect procedure. Eur Neuropsychopharmacol. 2000;10(3):369. doi:10.1016/S0924-977X(00)80495-2

[34] Deutsche Gesellschaft für Allgemeinmedizin und Familienmedizin e. V.(DEGAM). DEGAM-Leitlinie Nr 12: Demenz. Omikron Publishing; 2008.

[35] Deutsche Gesellschaft für Psychiatrie. Psychotherapie und Nervenheilkunde (DGPPN). S3-Leitlinie “Demenzen.” August 1, 2015. Accessed September 16, 2016. https://www.dgn.org/images/red_leitlinien/LL_2015/PDFs_Download/Demenz/REV_S3-leiltlinie-demenzen.pdf

[36] Hort J, O’Brien JT, Gainotti G, . EFNS guidelines for the diagnosis and management of Alzheimer’s disease. Eur J Neurol. 2010;17(10):1236-1248. doi:10.1111/j.1468-1331.2010.03040.x 20831773

[37] Rabins PV, Blacker D, Rovner BW, . American Psychiatric Association practice guideline for the treatment of patients with Alzheimer’s disease and other dementias: second edition. Am J Psychiatry. 2007;164(12 suppl):5-56. 18340692

[38] Fib T, Thyrian JR, Wucherer D, . Medication management for people with dementia in primary care: description of implementation in the DelpHi study. BMC Geriatr. 2013;13:121. doi:10.1186/1471-2318-13-121 24225205 PMC3840668

[39] Thyrian JR, Eichler T, Pooch A, . Systematic, early identification of dementia and dementia care management are highly appreciated by general physicians in primary care—results within a cluster-randomized-controlled trial (DelpHi). J Multidiscip Healthc. 2016;9:183-190. doi:10.2147/JMDH.S96055 27143912 PMC4844257

[40] Eichler T, Thyrian JR, Fredrich D, . The benefits of implementing a computerized intervention-management-system (IMS) on delivering integrated dementia care in the primary care setting. Int Psychogeriatr. 2014;26(8):1377-1385. doi:10.1017/S1041610214000830 24811145

[41] Cummings JL. The Neuropsychiatric Inventory: assessing psychopathology in dementia patients. Neurology. 1997;48(5)(suppl 6):S10-S16. doi:10.1212/WNL.48.5_Suppl_6.10S 9153155

[42] Zank S, Schacke C, Leipold B. Berliner Inventar zur Angehörigenbelastung—Demenz (BIZA-D). Z Klin Psychol Psychother. 2006;35(4):296-305. doi:10.1026/1616-3443.35.4.296

[43] Logsdon RG, Gibbons LE, McCurry SM, Teri L. Assessing quality of life in older adults with cognitive impairment. Psychosom Med. 2002;64(3):510-519. doi:10.1097/00006842-200205000-00016 12021425

[44] Ware J Jr, Kosinski M, Keller SDA. A 12-Item Short-Form Health Survey: construction of scales and preliminary tests of reliability and validity. Med Care. 1996;34(3):220-233. doi:10.1097/00005650-199603000-00003 8628042

[45] Holt S, Schmiedl S, Thürmann PA. Potentially inappropriate medications in the elderly: the PRISCUS list. Dtsch Arztebl Int. 2010;107(31-32):543-551. doi:10.3238/arztebl.2010.0543 20827352 PMC2933536

[46] Folstein MF, Robins LN, Helzer JE. The Mini-Mental State Examination. Arch Gen Psychiatry. 1983;40(7):812. doi:10.1001/archpsyc.1983.01790060110016 6860082

[47] Gauggel S, Birkner B. Validity and reliability of a German version of the Geriatric Depression Scale (GDS). Zeitschrift für Klinische Psychologie-Forschung und Praxis. 1999;28(1):18-27. doi:10.1026//0084-5345.28.1.18

[48] Hindmarch I, Lehfeld H, de Jongh P, Erzigkeit H. The Bayer Activities of Daily Living Scale (B-ADL). Dement Geriatr Cogn Disord. 1998;9(suppl 2):20-26. doi:10.1159/000051195 9718231

[49] Wimo A, Jonsson L, Zbrozek A. The Resource Utilization in Dementia (RUD) instrument is valid for assessing informal care time in community-living patients with dementia. J Nutr Health Aging. 2010;14(8):685-690. doi:10.1007/s12603-010-0316-2 20922346

[50] Faria R, Gomes M, Epstein D, White IR. A guide to handling missing data in cost-effectiveness analysis conducted within randomised controlled trials. Pharmacoeconomics. 2014;32(12):1157-1170. doi:10.1007/s40273-014-0193-3 25069632 PMC4244574

[51] White IR, Royston P, Wood AM. Multiple imputation using chained equations: issues and guidance for practice. Stat Med. 2011;30(4):377-399. doi:10.1002/sim.4067 21225900

[52] Cohen J. Statistical Power Analysis for the Behavioral Sciences. 2nd ed. Lawrence Erlbaum; 1988.

[53] van Assen MA, van Aert RC, Wicherts JM. Meta-analysis using effect size distributions of only statistically significant studies. Psychol Methods. 2015;20(3):293-309. doi:10.1037/met0000025 25401773

[54] Seidl H, Bowles D, Bock JO, . FIMA–questionnaire for health-related resource use in an elderly population: development and pilot study. Article in German. Gesundheitswesen. 2015;77(1):46-52. doi:10.1055/s-0034-1372618 24806594

[55] Byford S, Torgerson DJ, Raftery J. Economic note: cost of illness studies. BMJ. 2000;320(7245):1335. doi:10.1136/bmj.320.7245.1335 10807635 PMC1127320

[56] Briggs AH, Gray AM. Handling uncertainty in economic evaluations of healthcare interventions. BMJ. 1999;319(7210):635-638. doi:10.1136/bmj.319.7210.635 10473486 PMC1116497

[57] Michalowsky BFS, Dreier A, Eichler T, . Healthcare utilization and total cost from payer and societal perspective in primary care patients with dementia—baseline results of the DelpHi-trial. Eur J Health Econ. doi:10.1007/s10198-017-0869-7 28160100

[58] Manca A, Hawkins N, Sculpher MJ. Estimating mean QALYs in trial-based cost-effectiveness analysis: the importance of controlling for baseline utility. Health Econ. 2005;14(5):487-496. doi:10.1002/hec.944 15497198

[59] Willan AR, Briggs AH. *Statistical Analysis of Cost-Effectiveness Data*. John Wiley & Sons; 2006. 10.1586/14737167.6.3.337 20528526

[60] Obenchain RL. Resampling and multiplicity in cost-effectiveness inference. J Biopharm Stat. 1999;9(4):563-582. doi:10.1081/BIP-100101196 10576404

[61] Brodaty H, Arasaratnam C. Meta-analysis of nonpharmacological interventions for neuropsychiatric symptoms of dementia. Am J Psychiatry. 2012;169(9):946-953. doi:10.1176/appi.ajp.2012.11101529 22952073

[62] Livingston G, Kelly L, Lewis-Holmes E, . Non-pharmacological interventions for agitation in dementia: systematic review of randomised controlled trials. Br J Psychiatry. 2014;205(6):436-442. doi:10.1192/bjp.bp.113.141119 25452601

[63] Dias A, Dewey ME, D’Souza J, . The effectiveness of a home care program for supporting caregivers of persons with dementia in developing countries: a randomised controlled trial from Goa, India. PLoS One. 2008;3(6):e2333. doi:10.1371/journal.pone.0002333 18523642 PMC2396286

[64] Gaugler JE, Mittelman MS, Hepburn K, Newcomer R. Clinically significant changes in burden and depression among dementia caregivers following nursing home admission. BMC Med. 2010;8:85. doi:10.1186/1741-7015-8-85 21167022 PMC3012012

[65] Yeh SH, Johnson MA, Wang ST. The changes in caregiver burden following nursing home placement. Int J Nurs Stud. 2002;39(6):591-600. doi:10.1016/S0020-7489(01)00055-4 12100870

[66] Lam LC, Lee JS, Chung JC, Lau A, Woo J, Kwok TC. A randomized controlled trial to examine the effectiveness of case management model for community dwelling older persons with mild dementia in Hong Kong. Int J Geriatr Psychiatry. 2010;25(4):395-402. doi:10.1002/gps.2352 19606455

[67] Vickrey BG, Mittman BS, Connor KI, . The effect of a disease management intervention on quality and outcomes of dementia care: a randomized, controlled trial. Ann Intern Med. 2006;145(10):713-726. doi:10.7326/0003-4819-145-10-200611210-00004 17116916

[68] Jansen AP, van Hout HP, Nijpels G, . Effectiveness of case management among older adults with early symptoms of dementia and their primary informal caregivers: a randomized clinical trial. Int J Nurs Stud. 2011;48(8):933-943. doi:10.1016/j.ijnurstu.2011.02.004 21356537

[69] Eloniemi-Sulkava U, Notkola IL, Hentinen M, Kivelä SL, Sivenius J, Sulkava R. Effects of supporting community-living demented patients and their caregivers: a randomized trial. J Am Geriatr Soc. 2001;49(10):1282-1287. doi:10.1046/j.1532-5415.2001.49255.x 11890485

[70] Newcomer R, Miller R, Clay T, Fox P. Effects of the Medicare Alzheimer’s disease demonstration on Medicare expenditures. Health Care Financ Rev. 1999;20(4):45-65. 11482124 PMC4194605

[71] Reuben DB, Gill TM, Stevens A, . D-CARE: the Dementia Care Study: design of a pragmatic trial of the effectiveness and cost effectiveness of health system-based versus community-based dementia care versus usual dementia care. J Am Geriatr Soc. 2020;68(11):2492-2499. doi:10.1111/jgs.16862 32949145 PMC8086629

[72] Pichon-Riviere A, Drummond M, Palacios A, Garcia-Marti S, Augustovski F. Determining the efficiency path to universal health coverage: cost-effectiveness thresholds for 174 countries based on growth in life expectancy and health expenditures. Lancet Glob Health. 2023;11(6):e833-e842. doi:10.1016/S2214-109X(23)00162-6 37202020

[73] Bohlken J, von Stillfried D, Schulz M. Prevalence rates of mild cognitive impairment and of dementia in the German outpatient health care sector 2009-2016. Article in German. Psychiatr Prax. 2020;47(1):16-21. 31671469 10.1055/a-1012-9502

