# Supplementary material for: Clinical Outcomes and Cost-Effectiveness of Collaborative Dementia Care: A Secondary Analysis of a Cluster Randomized Clinical Trial
Source: JAMA Netw Open. 2024 Jul 5;7(7):e2419282. doi: 10.1001/jamanetworkopen.2024.19282 (PMC11227088; doi:10.1001/jamanetworkopen.2024.19282)
Supplement: Supplement 1. — Trial Protocol and Statistical Analysis Plan [file jamanetwopen-e2419282-s001.pdf]

Application for approval of the project

"Dementia: Life- and person-centred help in Mecklenburg Western Pommerania"
-
(DelpHi-MV) Study

to the Ethics Committee of the Ernst Moritz Arndt University of Greifswald

Proposer:
Prof. Dr. Wolfgang Hoffmann, MPH
Head of the CIDC Greifswald site
Deputy spokesman of the CIDC
Deputy spokesperson of the DZNE Rostock/ Greifswald site
Managing Director of the Institute for Community Medicine
Ellernholzstr. 1-2
17489 Greifswald
Tel.: +49 3834 86 77 50
FAX +49 3834 86 77 52
wolfgang.hoffmann@uni-greifswald.de

PD Dr. rer. med. habil. J. René Thyrian, Dipl.-Psych,
Scientific coordinator of the Greifswald branch of the DZNE Rostock/Greifswald site
Institute for Community Medicine
Ellernholzstr. 1-2
17489 Greifswald
Tel.: +49 3834 86 75 92
Fax: +49 3834 86 19 551
rene.thyrian@dzne.de

Cover letter to the responsible ethics committee

Dear Sir or Madam

We hereby apply for the approving evaluation of the project "Dementia: environment and person-oriented help in Mecklenburg-Vorpommern" - (DelpHi-MV) Study
We declare that **no further application for approval** of the above-mentioned project has been or will be submitted to any other ethics committee established under national law.

Please refer to **the attached annexes** for further application documents.

The applicant bears the **costs** of the review by the Ethics Committee.

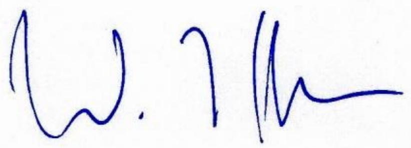

Prof. Dr. Wolfgang Hoffmann, MPH PD

Table of contents

1 Formalities 4
1.1 Title of the Study 4
1.2 Responsible Director 4
1.3 Type and number of test centers or participating physicians 4
1.3.1 Study center: 4
1.3.2 Cooperation partners: 5
1.4 Cost bearer 6

2 Project questions 7
2.1 Background 7
2.2 Objectives 10

3 Detailed description of the project 12
3.1 Recruitment of the test cohort 12
Screening 15
Contacting the test person 16
Assignment to the study condition 16
Basic survey of the test subject 16
3.2 Planned surveys and analyses 18
3.2.1 Screening 18
3.2.2 Baseline-Survey 19
3.2.3 Biomaterials 22
3.3 Intervention 23
3.3.1 Individual treatment and care plan 23
3.3.2 Medication 24
3.3.3 Systematic advice for relatives/HABC monitor 25
3.4 Relevance oft he project fort he care of people with dementia 25

4 Study Plan and Study Design 25
4.1 Type of Study 25
4.2 Continuation and Add-ons 26
4.3 Estimation of the number of cases 26
4.4 Statistical Methods 26
4.5 Storage of Biomaterials (Biobanking) 27
4.6 Quality assurance and Quality control 27
4.7 Data management 27

5 Survey methods and instruments 28
5.1 Interview 28
5.2 Blood sampling 28

5.3 Secondary data 28
5.4 Invasiveness 28
5.4.1 Quality assurance 29

6 Subject information and informed consent 29
6.1 Informing the subjects about the objectives and procedure of the study 29
6.2 Declaration of consent, Capacity to consent 30
6.3 Exceptions to obtaining consent 33
6.4 Extension of the research question 33

7 Confidential Treatment of data 33
7.1 Type and scope of encryption 33

8 Legal relationships 34
8.1 Subject insurance 34
8.2 Patient-fee 35
8.3 Reimbursement of expenses 35

9 Biological materials / Genetic Marker 35
9.1 Type of obtained materials 35
9.2 Declaration of consent 35
9.3 Subsequent expansion of the research question and recourse to existing materials 35
9.4 Notification of findings 35
The results of genetic and other low informative (i.e. from a medical point of view for quality of life, progression, therapy or diagnostics. The APoE status mentioned above, for example, is important for the differentiation of dementia, as it represents a risk factor, especially for Alzheimer's dementia. However, no treatments can be derived from this knowledge [45]. 35

10 Communication of the Results 36
10.1 Scientific Publication 36
10.2 Information of Test Subjects, Relatives, GPs 36
10.3 Communication to decision-makers, general public 36

11 Attachments 37
11.1 Participant information for test subjects with suspected dementia 37
11.2 Participant information Respondent without cognitive impairment 38
11.3 Declaration Test Subject 39
11.4 Declaration of consent by relatives of the test person 40

11.5 Declaration of consent for subjects without cognitive impairments 41
11.6 Consent to blood sampling **Error! Bookmark not defined.**
11.7 Ethics vote IDemUck study **Error! Bookmark not defined.**
11.8 Data protection concept of the Institute of Community Medicine**Error! Bookmark not defined.**
11.9 Ethics application for advanced diagnostics **Error! Bookmark not defined.**

1 Formalities

1.1 Title of the Study

Demenz: lebenswelt- und personenzentrierte Hilfen in Mecklenburg-Vorpommern - DelpHi-MV

1.2 Responsible Director

Prof. Dr. Wolfgang Hoffmann, MPH
Head of the CIDC Greifswald site
Deputy spokesman of the CIDC
Deputy spokesperson of the DZNE Rostock/ Greifswald site
Managing Director of the Institute for Community Medicine
Ellernholzstr. 1-2
17489 Greifswald
Tel.: +49 3834 86 77 50
FAX +49 3834 86 77 52
wolfgang.hoffmann@uni-greifswald.de

Scientific coordination:
PD Dr. rer. med. habil. J. René Thyrian, Dipl.-Psych,
Scientific coordinator of the Greifswald branch of the DZNE Rostock/Greifswald site
Institute for Community Medicine
Ellernholzstr. 1-2
17489 Greifswald
Tel.: +49 3834 86 75 92
Fax: +49 3834 86 19 551
rene.thyrian@dzne.de

1.3 Type and number of test centers or participating physicians

1.3.1 Study center:

The DelpHi-MV study is being conducted at the Center for Integrated Dementia Care Research (CIDC), a scientific cooperation between the universities and university hospitals of Greifswald and Rostock, and the German Center for Neurodegenerative Diseases. The study

center is located at the Greifswald branch of the DZNE Rostock/Greifswald site and at the Institute for Community Medicine at the Ernst Moritz Arndt University of Greifswald.
The study is scientifically coordinated by PD Dr. rer. med. habil. J. René Thyrian, Dipl.-Psych, Greifswald branch of the DZNE Rostock/Greifswald site.

1.3.2 Cooperation partners:

Various CIDC working groups are cooperating in the DelpHi-MV study:

- from the "Analytical Epidemiology and Healthcare Epidemiology" department“ (Prof. W. Hoffmann, Institut für Community Medicine, EMAU Greifswald)
  - Working group 5: prospective intervention study
  - Working group 11: Outpatient care managers
  - Working group 12 Qualification
- from the "Population-related psychiatric care research" department (Prof. H. Freyberger, Prof. H-J Grabe, Institute for Social Psychiatry, EMAU Greifswald)
- Working group 16: Population-based psychiatric care research
- from the „Neuroepidemiology“ department (Prof. U. Schminke, Clinic and Polyclinic for Neurology, EMAU Greifswald)
  - Working group 15: Neuroepidemiology
- from the „Health economics“ department (Prof. S. Fleßa, Chair of Health Management, EMAU Greifswald)
  - Working group 7: Health economics
- from the „Sociodemographics“ department (Prof. G. Doblhammer, Institute for Sociology and Demography, University of Rostock)
  - Working group 6: „Demographics“
- from the „Clinical dementia research“ department (Prof. S. Teipel, Clinic and Polyclinic for Psychiatry and Psychotherapy, University of Rostock)
  - Working group 8: General practitioners
  - Working group 9: non-medical services
  - Working group 10: Medical services

The study is also supported by:

- National association for social psychiatry (Prof. Ingmar Steinhart)
- Alzheimergesellschaft Mecklenburg-Vorpommern e.V. (Frau U. Greve)
- Ministry of Social Affairs Mecklenburg-Western Pomerania (Herr Dr. Koepke)

1.4 Cost bearer

The DelpHi-MV study is funded by the University of Greifswald and the DZNE. The DZNE with its seven sites is funded by the BMBF and the DZNE host countries in a ratio of 90 to 10.

2 Project questions

2.1 Background

Despite an absolute decrease in the population in Mecklenburg-Vorpommern over the next three decades, an increase in the number of people over the age of 65 is expected [1]. This is associated with an increase in the number of cases of age-related diseases such as type 2 diabetes mellitus, hypertension and dementia. The prevalence of dementia in the population is already high. It is estimated that between 6% and 9% of the population over the age of 65 currently suffer from dementia in Germany and other industrialized countries. Around 1.1 million people in Germany currently suffer from dementia. It is still estimated that around 250,000 new cases are added each year [2]. An exemplary extrapolation of the prevalence for Mecklenburg-Vorpommern showed that around 19,300 people were suffering from dementia in 2005. Due to demographic change, this figure will increase to 34,700 to 36,800 sufferers by 2020. This corresponds to an increase of 80 to 91%. The largest increase in the number of patients is expected in the 85 to 90 age group. Due to the longer life expectancy of women, they make up a significantly larger group of dementia sufferers than men [3].

The presence of dementia is a complication for the treatment of many other diseases. For example, age-related diseases such as Parkinson's and incontinence are difficult to treat due to drug interactions between the cholinesterase inhibitors used in dementia and the anticholinergic therapeutic agents. In addition, both the risk and the degree of care dependency, the frequency of inpatient hospital stays and the risk of institutionalization increase for those affected. It is known that early treatment can delay the progression of dementia and that cognitive function deteriorates less than with placebo. Furthermore, improvements in activities of daily living are possible and the disease-related quality of life often improves [4].

However, adequate care for dementia patients does not only start at the patient level, but must also include the immediate environment from the outset, which includes relatives, an official guardian if necessary and the main caregiver. In the following, we use the terms main caregiver and relatives synonymously. The main burden of the illness is often borne by relatives and the patient's social environment over long periods of time. This causes changes in several dimensions, for example the physical and emotional health, the social network and also the financial status of the relative is affected [5]. There is an increase in depression and anxiety [6] immunological dysfunctions [7] social withdrawal [8] as well as serious psychiatric problems and an increased risk of mortality. [9]. Patients and relatives see the fragmentation of care and communication between individual service providers and between service providers

and patients as a particular problem [10]. A systematic review of current literature shows that caring for a relative with dementia is associated with negative effects on the carer's own health, which in turn can lead to premature institutionalization of the person with dementia[11]. This relationship is influenced by the gender of the caregiver, the relationship to the person with dementia, the culture and personal characteristics of the caregiver [12]. A Cochrane review of interventions for family caregivers found no clear evidence for any particular intervention and was withdrawn after publication due to the general overhaul of psychosocial reviews [13]. An update is currently in the editorial process (as of 01.02.2011). A current systematic review by the authors of the Cochrane Group describes that there is hardly any scientific evidence for the effectiveness of interventions to reduce the burden on relatives [14]. This is due to the fact that very few high-quality, randomized intervention studies have been conducted on the topic. Furthermore, the authors state that statements on the statistical significance of the results achieved in the context of interventions alone are not sufficient. Rather, the clinical significance and relevance of the change for the relatives should also be examined. Intervention studies must be carried out that meet the scientific quality requirements of care epidemiology and are relevant to population-based care. The scientific effort seems justified, as individual analyses have repeatedly shown that improving the situation of relatives and also improving the coordination of care for the patient can have personal, health and, not least, economic benefits [15;16].

Demographic change is leading to significant changes in the demands placed on the healthcare system in Germany. For cost reasons alone, but also due to the necessary further development of medical quality, these cannot be met with a quantitative increase in current capacities alone. Rather, the demographic and economic conditions of the next three decades require a conceptual and structural realignment of large parts of the healthcare system.

The DZNE

Founded in 2009 as a national research center, the German Center for Neurodegenerative Diseases in the Helmholtz Association (DZNE) aims to strengthen research in the field of neurodegeneration and to pool and support the expertise available in Germany in this area. The new center's work program includes research into the causes and risk factors that lead to the development of dementia as well as the development of new treatment and care strategies. The DZNE currently comprises 7 locations throughout Germany, including the Rostock/Greifswald site.

The Rostock/Greifswald site aims to analyze the medical and nursing care situation of older people with dementia in the demographic focus region of Mecklenburg-Vorpommern, to implement innovative subsidiary care concepts and to test their effectiveness using care

epidemiological methods. One focus is on the supra-regional significance of the findings obtained in Mecklenburg-Vorpommern for other regions of Germany. The Rostock/Greifswald site works closely with the participating universities and clinics in Rostock and Greifswald in order to work on the subject area together with them as part of a Center for Integrated Dementia Care Research (CIDC). Structurally, the Rostock/Greifswald site is divided into the Rostock sub-site and the Greifswald sub-site. The head of the Greifswald site is Prof. Dr. Wolfgang Hoffmann.

The prospective intervention study DelpHi-MV (Dementia: lifeworld and patient-centered help in Mecklenburg-Vorpommern - DelpHi-MV) is to be carried out at the Greifswald site in close cooperation with the DZNE, the University of Greifswald and the University of Rostock. As part of the DelpHi-MV study, the dementia-related need for medical and nursing care in the population is to be recorded and new care concepts developed, implemented and tested for their effectiveness in practice.

The Institut of Community Medicine

The Department of Healthcare Epidemiology and Community Health at the Institute of Community Medicine focuses on clinical and population-based projects in the fields of healthcare research and epidemiology. In this department, issues of data management, interdisciplinary transfer and consulting activities for clinical research projects also play an important role.

When conducting studies, the CIDC can draw on the expertise and experience gained at the Institute of Community Medicine since it was founded in 2002. As part of the AGnES studies, the concept of delegation of home visits by the attending physician to specially qualified specialists was developed and tested in four federal states [17-19]. As part of this research, a curriculum was developed for the specialized work of professionals and outreach care [20]. To check for drug-related problems, the concept of the home medication history was expanded and a network involving pharmacists and doctors was implemented [21].

The project "Interdisciplinary care and treatment network for dementia patients in the Uckermark district (IDemUck)", funded by the BMG as part of a lighthouse initiative, provides expertise in conducting care epidemiology studies with dementia patients. Some of the study instruments to be used in the Delphi MV study were developed and tested in this study (ethics vote on IDemUck in Appendix 11.7).

The Rostock Clinical Dementia Research Center

In cooperation with the Department of Psychiatry and Psychotherapy and the Department of Neurology at the Center for Neurology at the University of Rostock and the German Center for Neurodegenerative Diseases, there is a clinical research facility for the diagnosis and

treatment of patients with dementia at the Rostock site. This center offers guideline-based diagnosis of dementia, including clinical, psychiatric and neurological examination methods, neuropsychology, multimodal imaging and laboratory and cerebrospinal fluid diagnostics. The site has many years of experience in the diagnosis of dementia, the center has a close national and international network in the field of clinical dementia research and serves as a diagnostic reference center for the implementation of cerebral imaging in national and European multicenter studies. The close cooperation with the Greifswald site in the DelpHi study ensures the patient-related quality of diagnostics.

The study project

The aim of the DelpHi-MV study is the scientific evaluation of an innovative, integrative and subsidiary care concept to improve the care of dementia patients living at home. The design of DelpHi-MV is a GP-based, cluster-randomized, prospective intervention study. The core of the integrative care concept is a structured treatment program that is provided by specially qualified Dementia Care Managers (DCM). The DCMs analyze the medical, nursing and social situation of the test person on the basis of a detailed standardized survey of the test person and their caregiving relatives or other caregivers at home. If a need for intervention is identified, the DCM designs an individualized intervention on the basis of modularized action paths, taking regional factors into account, and coordinates its implementation.

The success of the intervention is evaluated multidimensionally for both the patient and the caregiver(s) and assessed in terms of health economics.

If this concept proves to be effective and efficient, the aim is to implement the entire concept or selected sub-functionalities in the healthcare system, from which the group of people suffering from dementia will benefit directly.

2.2 Objectives

The objectives of the DelpHi-MV study, the dependent variables (AV) of the study design, are:

1. an improvement in the guideline-based care of people with dementia, which is determined by both individual and regional factors. The goals are:
    a) the early detection of people suffering from dementia in the general population as part of basic medical care,
    b) the individual, adequate diagnosis and treatment of dementia and the relevant comorbidities (especially depression, hypertension, diabetes),
    c) the identification of the individual, personal needs and priorities of the person with dementia from the perspective of the person affected.
    d) the avoidance of emergency medical interventions and hospitalizations

e) optimizing the use of dementia-specific help for the person affected in a way that is adapted to their living environment,
f) improving the primary caregiver's knowledge of aspects of the disease, treatment and the regional care and support system,
g) improving the living conditions and life satisfaction of those affected,
h) the improvement of social participation, prevention of social isolation, the postponement of institutionalization or the identification of the most appropriate time for institutionalization, taking into account the overall medical and nursing situation, relevant individual characteristics of the person concerned and the main caregiver(s), as well as the social environment

2. the relief of the relatives or the main caregiver. The relative will often also be the main carer. However, this is not necessarily the case. In the following, both terms are used synonymously. The intervention varies greatly from person to person and depends on the home situation (person with dementia, relative, other people), the overall medical situation and regional circumstances. In general, the goals are:
   a) the reduction of challenging behavior of the person suffering from dementia,
   b) improving communication between the person with dementia and their main carer,
   c) improving information about the illness and facilitating the use of support services, e.g. care and social support in the regional care system,
   d) maintaining and, if necessary, improving the physical and mental health of the main carer,
   e) improving the use of support services that affect the well-being and health of the main carer,
   f) reducing the strain, burden and stress on the main carer,
   g) the prevention of social isolation of the main caregiver.

These goals are to be achieved through the implementation of a care concept (the use of a Dementia Care Manager, DCM). This represents the independent variable (UV) in the study design. The analysis of the effectiveness of the DCM is carried out by statistically comparing the operationalization of the aforementioned objectives between the intervention group and the control group at different points in time.

In addition, various parameters that may also be affected by the intervention and/or that may have an influence as moderator or mediator variables on the target variables are considered and controlled at the levels of (a) the test person, (b) the main caregiver(s) and (c) the care system. These are listed and described in detail in Chapter 3.3. In addition to the main analysis, pre-post comparisons for the control and intervention groups and comparisons between the control and intervention groups and non-cognitively impaired individuals are also carried out here. The comparison with non-cognitively impaired persons allows conclusions to be drawn about the extent to which the parameters and intervention effects recorded are specific to dementia.

Moderator variables are variables that influence (weaken or strengthen) the relationship between the intervention (UV) and the measured outcome (AV). Mediator variables are variables that underlie the relationship between the intervention (UV) and the measured outcome (AV).

At the subject level, moderating variables can be: well-being, cognitive status, frequency of hospitalization, institutionalization, somatic health, health-related quality of life, drug-related problems associated with the pharmacotherapy of dementia or concomitant diseases, independence in lifestyle, social integration, informedness. For example: How does the patient's cognitive status influence the drug therapy for dementia? How does the patient's health-related quality of life influence the burden on the relative?

At the level of the main caregiver(s), the following variables are examined as moderator or mediator variables: age, relationship to the caregiver, health, quality of life, psychiatric disorders, morbidity and multimorbidity, burden and stress, use of assistance, social integration, information, use of medical services. For example: How does the age or degree of kinship of the main caregiver influence the number of hospitalizations? How does the burden and stress of the main caregiver influence the reduction of behavioral problems in the test person?

At the level of the care system, the effectiveness of DCM and efficiency as the relationship between resources used and effectiveness.

3 Detailed description of the project

3.1 Recruitment of the test cohort

The planned DelpHi-MV study is a GP-based, care epidemiological, cluster-randomized, prospective, controlled intervention study. Figure 1 provides an overview of the study design. The study is being conducted by the Center for Integrated Dementia Care Research (CIDC) at the University of Greifswald.

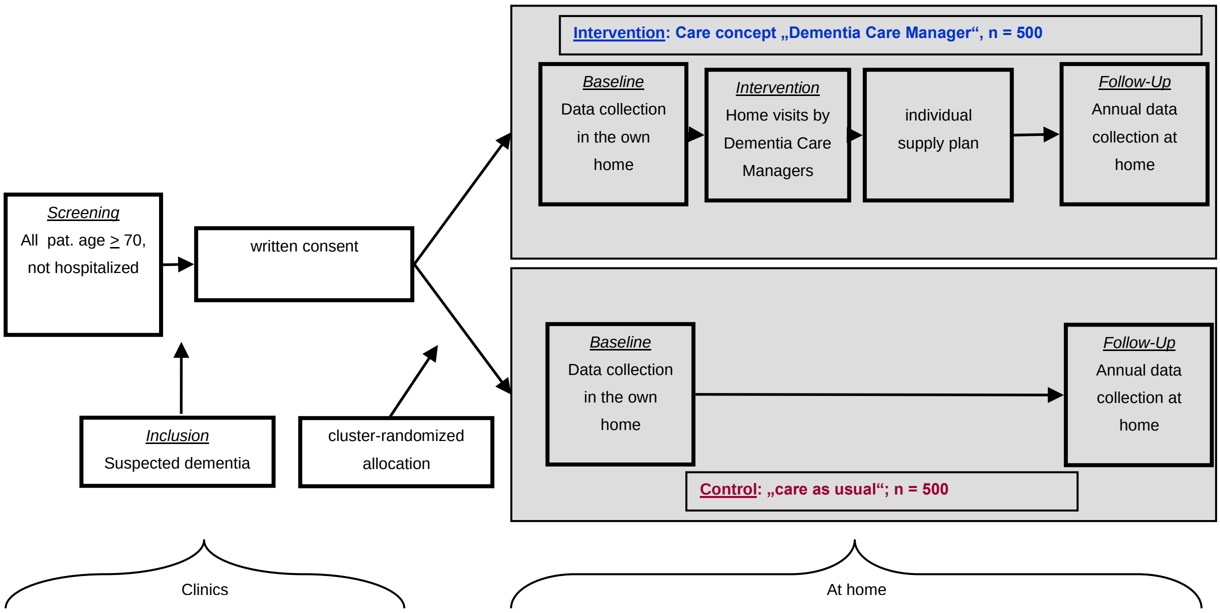

Figure 1: Study design for subjects with suspected dementia

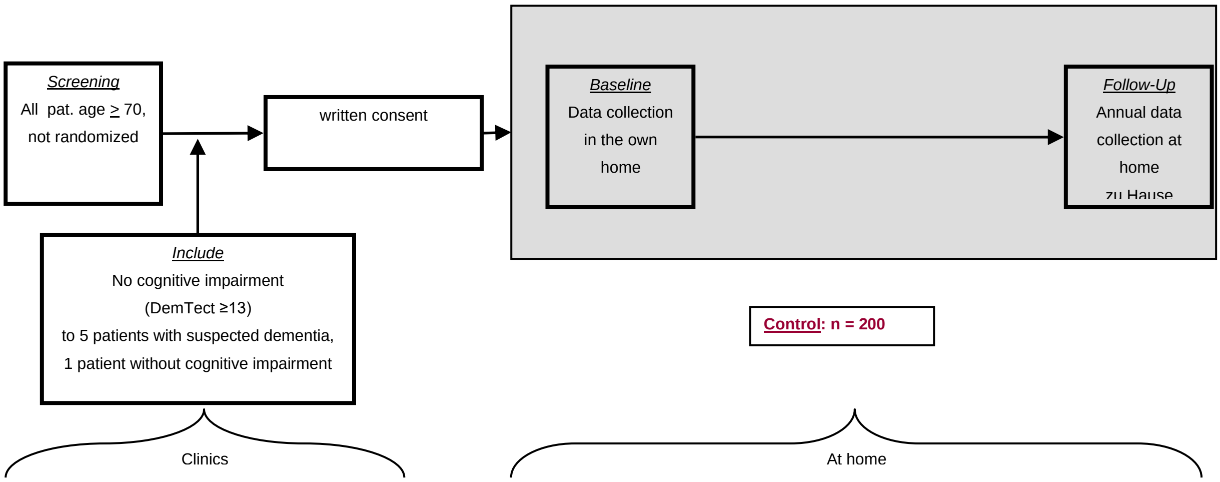

Figure 2: Study design for subjects without cognitive impairment

The DelpHi-MV study is planned for the period from March 2011 to December 2014. All men and women who visit their GP, are aged 70 or over and are suspected by their GP of having dementia will be asked to take part. The first contact is made by the GP. The GP carries out a simple screening test on patients who meet the inclusion criteria:

- 70 years or older; this criterion was chosen because it is expected that (a) the proportion of people screened positive for dementia in younger age groups is too low to justify general screening in GP practices. (b) the risk of selection bias by the GP is greater in younger age

groups (c) the workload of negatively screened persons in younger age groups leads to acceptance problems among the doctors carrying out the screening.
- Participant lives in a non-institutionalized setting (house, rented or owner-occupied apartment, shared flat, assisted living)
- Understanding of the German language
- No severe hearing impairments
- No severe visual impairments

The inclusion criteria for the DelpHi-MV study are:
- "Respondent with suspected dementia": Suspected dementia, regardless of any existing dementia diagnosis. Operationalization is carried out using the DemTect 22;23. With a DemTect ≤ 8, the criterion "suspected dementia" is fulfilled. Validation of the diagnosis in the home with the help of CERAD (Consortium to Establish a Registry for Alzheimer's Disease) 24. Differential diagnosis of the presence of depression with the help of the GDS (Geriatric Depression Scale) 25. The DemTect was selected because (a) it is already a widely used screening instrument in GP practices, (b) it is cost and time effective, (c) it can be carried out by trained practice staff, (d) results of GP screening in DelpHi-MV are comparable with results from IDemUck. The CERAD 24 was used as an internationally used, validated instrument for the diagnosis of dementia. The GDS 25 is a common procedure in the diagnosis of depression in older people.
- "Subject without cognitive impairment": To compare subjects with suspected dementia, subjects without cognitive impairment are also recruited using the same screening procedure, operationalized by a DemTect of ≥ 13. In DelpHi-MV, one subject without cognitive impairment is recruited for every 5 subjects screened positive for suspected dementia. These subjects serve as a healthy comparison group. Data collection in this group includes the same instruments and procedures as for the subjects with suspected dementia. There is no intervention in this group.
- The written consent of the test person or their authorized representative is available.

The course of the study consists of (a) a screening at the GP practice, (b) contacting and making an appointment with the study staff, (c) a visit at home and (d) further visits at regular intervals. Figure 3 shows the course of the study from the subject's

perspective.

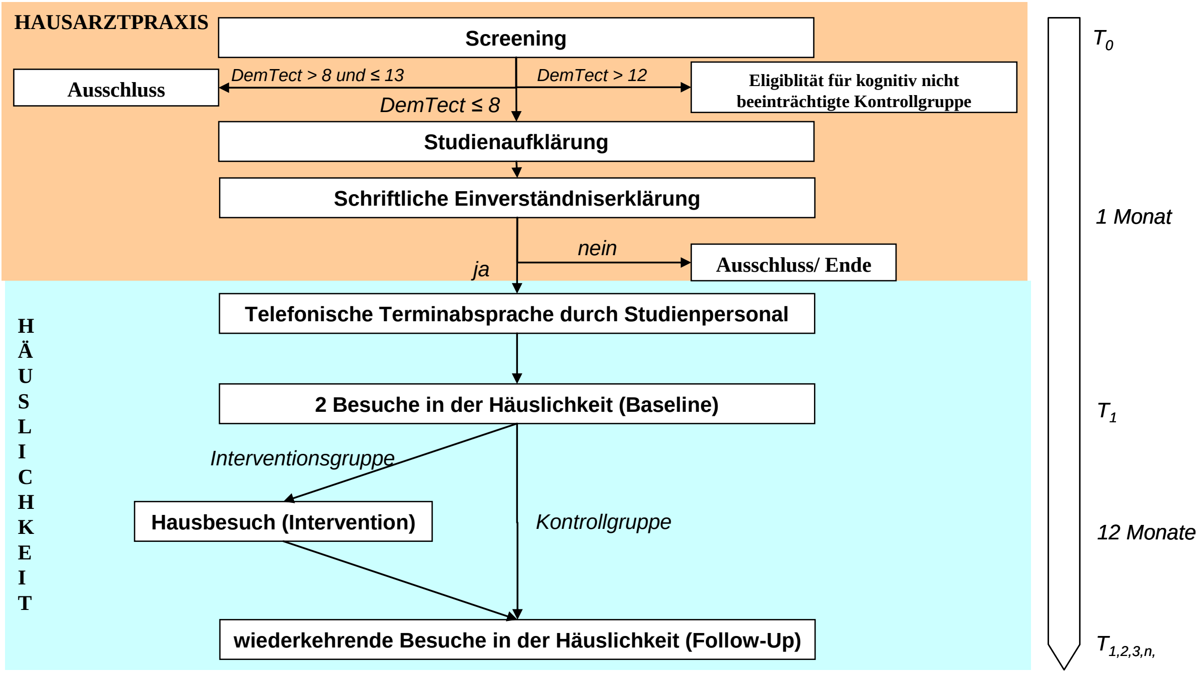

Figure 3: Course of the study from the subject's perspective

Screening

All patients who visit their GP during the course of the study and fulfill the inclusion criteria are asked to participate in the screening. If the screening is positive and the other inclusion criteria are also met, the GP will inform the patient about the aims and procedures of the DelpHi-MV study both verbally and in writing. As part of the explanation, the GP will hand out the written participant information (see Appendix 11.1 and 11.2) and explain it to the patient in detail. If the patient is willing to participate in the study, he/she fills out the written informed consent form (see Appendix 11.3), which turns him/her from a patient of the GP into a subject of the DelpHi-MV study. If the patient is not capable of giving consent, the supporting relative or a legal representative is also asked to provide written consent. This takes place after an analogous explanation and the handover of the written information.

At this point, the subject consents to the transfer of his or her personal data (name, address, telephone number) and screening result data to the DelpHi-MV study team. In addition, the proband releases the family doctor from his or her duty of medical confidentiality towards the study staff of the DelpHi-MV study, allows the team to visit him or her at home to conduct the study and (optionally) names a relative who can be asked to participate in the study. This relative is also released from the duty of confidentiality vis-à-vis the study team in order to enable the collection of anamnestic information from others. The next of kin will be informed about the study in the same way and a written declaration of consent for next of kin will be obtained (see Appendix 11.4).

Optionally, the subject can also consent to a blood sample being taken. The subject-related data is transmitted to the DelpHi-MV study management by personal transmission by the family doctor himself, his authorized practice staff or by study staff of the DelpHi-MV study.

Contacting the test person

Once the data has been submitted, it is checked by study staff for completeness, consistency and plausibility. Patients and relatives who meet the inclusion criteria are then contacted by the study team.

For this purpose, a member of the study team (if possible) contacts the patient and the relative (if available) by telephone and arranges an interview with the subjects, usually at the subject's home. If this is not desired by the test person, it is possible to conduct the initial contact at a suitable alternative location (e.g. local health authority, other cooperating facility). In addition to the appointment, the location of the initial contact is also agreed between the volunteer and the study team member and documented in the DelpHi volunteer management system.

Assignment to the study condition

The subjects are assigned to either the intervention or the control group before the survey. The allocation is cluster-randomized depending on the allocation of the screening GP practice. The screening GP practices are randomly allocated to the intervention or control arm beforehand. In the control group, the subjects are treated according to the usual medical and nursing procedures (usual care); in the intervention group, the integrative and optimized subsidiary care concept of the DelpHi-MV study is coordinated by the DCM.

Basic survey of the test subject

After contacting and making an appointment with the study staff, a standardized, interview-based survey of the subjects and the supporting relative(s) takes place at home. At the beginning of this interview, the most important study contents and objectives are explained again and the participants are given the opportunity to ask any questions concerning the study itself or its thematic and organizational environment.

The survey includes socio-demographic data, health-related information, data on the use of medical, nursing and social services, quality of life and various dimensions of the burden on relatives. Furthermore, detailed information on medication behavior is collected as part of a medication review. The survey is conducted on the basis of validated survey instruments. The parameters and instruments collected are presented in detail in Chapter 3.2 and Chapter 5.

After 12 months, a second standardized survey of the test subjects takes place. The content and procedure correspond to the initial survey.

As part of the intervention, additional data on the treatment and care of dementia that is relevant to individual care is collected from test subjects in the intervention group. For example, addresses of care services and other providers are collected, as well as data on health care in the form of prescribed aids, etc. This data collection is part of the DelpHi-MV care concept and is carried out on an indication-driven and individual basis as part of the intervention house visits carried out by the DCM.

Intervention

In addition to the surveys described in the methodology, the subjects assigned to the intervention condition receive an individualized intervention by the DCM. As part of the baseline interview instruments, possible intervention needs are identified in a standardized form. An individual, modular intervention plan is drawn up on the basis of the data collected in the interview. The subjects in the intervention group are integrated into a dementia-specific, individualized network, which is organized by the Dementia Care Manager. In preliminary work by the CIDC, the existing care structure in Mecklenburg-Vorpommern was analyzed and existing care services that are specifically suitable for dementia patients were compiled. In this context, for example, the existing dementia flat-sharing communities were identified for all regions in Mecklenburg-Vorpommern, addresses and contact details of relatives' groups were collected and other special support options were identified. The possible care services are made available to the test person in a tailored manner. The DCM will familiarize patients with care and support facilities in the region. If necessary, prescriptions for necessary services and referrals will be made by the family doctor, which will be initiated by the DCM.

Based on the patient's medication history at home, standardized documentation is created and forwarded to the patient's regular pharmacy. Using the data available in pharmacies (e.g. ABDA database, interaction monographs, etc.), the pharmacy checks the occurrence of selected drug-related problems and takes over pharmaceutical care in consultation with the GP. The GP discontinues any unsuitable or no longer required medication and optimizes the medication therapy. If necessary, the baseline survey is divided into up to three visits so as not to overburden the patient during the individual visits. If possible, the patient and relative interviews are conducted simultaneously in two separate rooms in order to exclude mutual interference as far as possible and to guarantee the confidentiality of the information between the interviewer and interviewee. Depending on the identified need for intervention, the DCM will organize and carry out individual monitoring visits.

Further Diagnostics

In a sub-sample of 20% of the people in the intervention group, further diagnostics will be carried out at the Rostock site to confirm the syndromal and etiological diagnosis in accordance with the relevant S3 guidelines on dementia. The aim of this diagnosis is to assess whether the diagnoses made in the primary care and home setting are predictive of the clinical diagnoses made in accordance with national guidelines. This is important because it allows the comparability of the study population with other national and international cohorts to be assessed. It also allows an assessment of the distribution of different etiologies for dementia syndromes in the overall sample, so that hypotheses on the differential effectiveness of interventions can be tested. Patients in the sub-sample will also be offered participation in an add-on study to assess the diagnostic quality of biomarkers of dementia, which will be carried out in addition to the diagnostic validation at the Rostock site. For the patients of the add-on study, persons are selected from the diagnostic validation sample who suffer from mild to moderate cognitive impairment, according to an MMSE score of at least 15 points and who do not have any metallic implants or pacemakers that would preclude the performance of a cranial MRI. The results of the diagnostic validation are reported back to the responsible general practitioner in the form of a medical letter. In addition to the diagnosis, this letter can also contain specific therapeutic recommendations based on the diagnostic information obtained. This is the reason why further specific diagnostics are only offered in the intervention group. Influencing the control group is avoided. The implementation of the further diagnostics and the further biomarker evaluation is described in a separate ethics application, which was submitted in parallel by the Rostock site to the ethics committee of the medical faculty of the University of Rostock and a copy of which is enclosed with this application (see Annex 11.9).

3.2 Planned surveys and analyses

3.2.1 Screening

As part of the screening

a) memory-related parameters

b) a test of visual-spatial organization and

c) personal data are collected.

An overview of the parameters collected and their operationalization can be found in Table 1.

Table 1: Parameters and their operationalization in screening

Topic	Content	Operationalization
a) Memory-related parameters	Subjective<br>Memory impairment	2 items [26;27]
	Suspected dementia	DEMTECT [22;23]
b) Visual-spatial organization	Watch test	[28;29]
c) Personal data		

3.2.2 Baseline-Survey

As part of the baseline survey, data is collected

a) on socio-demographics,
b) on the general state of health,
c) on medical status,
d) on depression,
e) psychiatric abnormalities
f) cognitive performance,
g) (instrumental) activities of daily living,
h) quality of life,
i) on social support,
j) stress and psychopathology of the relative
k) on the utilization of care services,
l) information about the illness and care services,
m) on the intake of medication collected.

An overview of the parameters surveyed and their operationalization can be found in Table 2.

Table 2: Parameters and their operationalization to the baseline

Topic	Content	Operationalization
Socio-demographics		Abridged interview module from SHIP und IDemUck
health-related quality of life	Physical functioning<br>Physical role function<br>Pain<br>Allgemeine Health perception	SF-12[30]

	Vitality<br>Social functioning<br>Emotional role function<br>Psychological well-being	
		STEP
General<br>medical status,		
Depression	None, mild to moderate, severe depression	GDS
Psychiatric abnormalities	None, mild to moderate, severe depression<br>Delusions<br>Hallucinations<br>Agitation/ aggression<br>Depression/ dysphoria<br>Anxiety<br>Elation/ euphoria<br>Apathy/ indifference<br>Disinhibition<br>Irritability/ lability<br>Deviant motor behavior<br>Sleep<br>Appetite and eating disorder	NPI [31]
mental performance,	Memory<br>language<br>constructive practice	CERAD
(instrumental) activities of daily living,	General assessment<br>Difficulties with certain activities<br>Cognitive abilities	B-ADL [32;33]
Activities of daily living<br>(internationally used instrument	Actual performance of everyday activities and extent of assistance required assistance	ACDS-ADL [34]
Health-related quality of life,	physical condition<br>Mood	QoLA-D [35]

	memory<br>functional abilities,<br>social contacts<br>Ability to participate in meaningful activities<br>Dealing with money<br>self-assessment<br>global QoL assessment.	
	Mental well-being	SF-12
Social support,	Emotional support	FSozU [36]
Burden on the relative,	Subjective stress<br>a) Physical exhaustion<br>b) Personal restriction<br>c) Lack of social recognition<br>d) Personal development<br>e) Negative evaluation of own care services<br>Objective burden<br>a) basic and extended care tasks<br>b) motivating and instructing<br>c) Support in maintaining contact<br>d) emotional support<br>e) Supervision	BIZA-D [37]
Mental stress of the relative	Depressiveness<br>anxiety<br>somatization	BSI-18 [38]
Psychopathology of the relative	Major depressive syndrome<br>Other depressive syndrome<br>anxiety disorder	PHQ-9 [39;40]
Utilization of utility services	utilization of medical (doctor's appointments, remedies and aids, etc.)<br>nursing care (e.g. outpatient care, day care)<br>- and other (transportation	1. self-created questionnaire<br>2. RUD

	services, home help, self-help group)<br>- Care services (patient and relatives)<br>2. time spent on and productivity losses due to informal care/support (relatives)	
Informed about the illness and care services,<br>Taking medication		

3.2.3 Biomaterials

As part of the DelpHi-MV study, blood samples are to be taken at home. Information about this is provided by the screening family doctor. Consent for the blood sample to be taken is obtained again in writing after further written and verbal explanation at home (see Appendix 11.6). The blood sample is taken for 2 purposes:

1. collection of DNA for genetic phenotyping of the subjects
2. validation of innovative biomarkers that are to be used in routine practice.

ad 1 For example, the determination of the ApoE genotype is described for the diagnostic SOP of dementia diagnostics [41]. DNA is required for these analyses. According to the current state of knowledge, ApoE should be included in the subsequent main analyses for effectiveness and efficiency.
Another focus of the DNA studies is the discovery of new genetic markers and their validation in a population-based patient cohort. Other important research questions concern the association between genetic markers and the results of certain imaging procedures, in particular functional MRI.

ad 2 There are a large number of biomarkers in clinical research, but to date only a few tests have found their way into routine use. An overview of perspectives in clinical proteomics optimistically describes the neurochemistry of Alzheimer's disease, stating that the identification of a biomarker for early detection is possible, but that there are large differences in the results and reference categories. It is also stated that the results are often based on individual studies and that replications are often still pending [42]. A consortium funded by the EU's 7th Framework Research Program explicitly describes cerebrospinal fluid and blood as promising biomaterials and describes the current status of the identification of

suitable proteins. No blood parameters are currently included in a diagnostic SOP for clinical dementia diagnostics. At the same time, however, the authors emphasize that blood parameters must be investigated for routine use [41].

3.3 Intervention

In order to achieve the objectives of the study described in Chapter 2.2, the DCM will carry out an intervention consisting of 3 modules.

a) the creation of an individual treatment and care plan
b) optimization of the subject's drug therapy as part of pharmaceutical care
c) systematic advice and support for the main caregiver

The individual modules are described in detail below.

3.3.1 Individual treatment and care plan

The tasks/activities of the DCM include the creation and implementation of an individual treatment plan:

a) the analysis of the individual medical-therapeutic, nursing and social situation of the patient
b) the individual determination of the specific resources, deficits and needs of the patient and the main caregiver(s)
c) Identification of available services in the medical and social care system for (1) the test person and (2) the main caregiver(s), including their availability, accessibility and individual access options
d) the development of an individualized care plan for the patient, family member(s) or caregiver(s)
e) the coordination and implementation of the individual care plan; the monitoring of the implementation and the resulting effects, on this basis adjustment and further development if necessary

The standard procedure for the intervention begins with an initial intervention visit to the test person's home. Here, specific needs and recommendations for action are analyzed and discussed. Within the following month, further telephone contact is made with the test person or their relatives to monitor/document and promote changes compared to the previous intervention visit. This is followed by an intervention contact by telephone at least once a month.

At the subject's request and depending on the complexity of the treatment plan, the follow-up intervention visits can take place in person in whole or in part, for example at home. For

budget and time reasons, however, home visits for intervention are to be limited to the absolutely necessary number of visits.

The type of intervention visits and all information and intervention activities collected are documented.

Following the baseline assessment and the first intervention visit, the DCM is responsible for reporting the treatment-relevant results back to the GP. This is done as standard by means of a short letter. The content of the letter, which is no longer than 1 DIN A4 page, consists of feedback on the social history, the results of the neuropsychological and neuropsychiatric tests, identified needs in care and contains treatment recommendations.

The treatment recommendations are developed based on the data from the baseline survey as part of a weekly case conference with a geriatric specialist. Each test person is introduced by the DCM and their treatment recommendations are reviewed and documented together with the geriatrician. This procedure ensures the development of quality-tested algorithms for later independent application by the DCM.

The implementation of this module is an essential core of the Dementia Care Manager qualification. It is based on a curriculum that was developed for this activity and will be evaluated and, if necessary, optimized during the pilot phase.

3.3.2 Medication

On the basis of a systematic medication review, which is carried out by the DCM using IT in the patient's home, systematic documentation is created for forwarding to the patient's home pharmacy. The pharmacist carries out the intervention as part of medication management using the so-called SOAP scheme. First, SUBJECTIVE problems such as adverse drug reactions and lack of adherence are evaluated and then OBJECTIVE problems such as the occurrence of clinically relevant drug reactions are considered and classified according to clinical relevance. A PLAN for the next steps is then developed on the basis of the ANALYSIS. The goals set are discussed at regular intervals and new steps are discussed. For the procedure in the DelpHi-MV study, this means that the pharmacist contacts the test person and, if possible, a relative as part of the intervention and gives tips on how to improve medication intake (correct intake in terms of the time of intake) and adherence. Options for improving adherence include drawing up a medication plan, using medication dispensers and reminder systems (initially stickers for the bathroom mirror). In order to be able to document special medication-related events, the test person receives a therapy diary.

The plan and results of the medication management intervention are recorded by the pharmacist on a documentation sheet and forwarded to the supervising GP. The GP has therapeutic responsibility and decides on possible changes to pharmacotherapy in terms of

dose adjustment, new prescriptions or discontinuation of medication. Possible problem cases should be discussed in regular quality circles.

3.3.3 Systematic advice for relatives/HABC monitor

A semi-structured interview is conducted with the relative at each contact, which is based on the contents of the HABC monitor. At each contact, 28 areas of everyday life are systematically classified in terms of their frequency of occurrence (from 0 = never to 3 = almost daily). This serves to identify and assess 13 dementia-specific problem areas in the daily life of the test person and their relative. (stress, movement, communication, legal & financial, physical health, depression/ anxiety, repetitive behavior, aggression/ agitation, mobility, personal hygiene, sleep disturbance, hallucinations/ paranoia, delirium).
The problem areas identified in this way are the content of the intervention carried out during this visit. A maximum of 2 problem areas are intervened per visit. These depend on the frequency of occurrence (systematically determined) as well as on the subjective stress caused by this problem (determined in discussion with the patient/relatives). There are standardized protocols for each problem area, i.e. instructions for the DCM on what information to convey during the conversation and what to actively organize or otherwise initiate. Each protocol contains a number of information sheets for the test persons and relatives, which are issued to them according to a fixed algorithm and explained accordingly.

3.4 Relevance oft he project fort he care of people with dementia

The aim of the DelpHi-MV study is the scientific evaluation of an innovative, integrative care concept to improve the care of dementia patients living at home. The integrative care concept represents a structured treatment program by specially qualified Dementia Care Managers (DCM). If this concept proves to be effective, efficient and efficient, the aim is to implement the overall concept or relevant sub-modules of the concept in the healthcare system in the medium term, from which the group of people suffering from dementia will benefit directly.

4 Study Plan and Study Design

4.1 Type of Study

GP-based, health care epidemiological, cluster-randomized, prospective, controlled intervention study

4.2 Continuation and Add-ons

As part of the study, an annual follow-up takes place at home. The subjects are contacted exclusively by the study staff, if possible even by the same person as at the initial contact.

Additional examinations/questions and sub-projects that are not explicitly mentioned in this project description will only take place with the written informed consent of the subjects. For these "add-ons", written authorization is given to pass on the contact details. Such add-ons are intended to enable the subjects to benefit from current developments and new research results during the ongoing DelpHi-MV study. The contact details will not be passed on to third parties.

4.3 Estimation of the number of cases

The size of the targeted cohort (n = 981 test subjects and their relatives) is due to the fact that smaller cohorts would not be meaningful with regard to the effectiveness of the intervention.

From the IDemUck study, for example, a small to medium main effect can be expected with regard to the medication of dementia patients. According to preliminary analyses, the intervention there increased the intake of anti-dementia medication in subjects with dementia by 25.8% (from 25.8% to 51.6%). In the same period, the intake in the control group increased by 8.5% (from 27.4% to 35.9%). This difference between the two groups corresponds to an effect size of h = 0.461. According to Cohen's criteria, this corresponds to a small effect. A smaller effect can be expected for the DelpHi-MV study, as no selection of the participating GPs was made as in IDemUck.

In order to statistically validate the effectiveness of the intervention compared to a control group with a small effect (h=0.2), a sample size of n = 309 subjects is required for the intervention group for a comparison at a significance level of α=0.05 with a statistical power of 80%. This corresponds to a total study group of n = 618. With the longitudinal design of the study, however, a "loss due to follow-up" must also be expected, which is calculated at 30% per survey time point. We also expect a mortality rate of approx. 10%, as described in the LEILA75+ study, for example [43]. This results in n = 981.

4.4 Statistical Methods

All statistical analyses are carried out using standard software with documentable syntax (SAS, SPSS, stata, etc.). The statistical analyses of the study data are based on many years of expertise at the Institute of Community Medicine. For prevalence and incidence estimates

and trend statistics, the study data are weighted against an external standard. Association analyses are performed multivariably, taking into account relevant confounders for the relationships under investigation.

4.5 Storage of Biomaterials (Biobanking)

The materials not used for direct analysis are pseudonymized and stored in a biobank. These are aliquots of serum, plasma and DNA that are to be stored for later analysis of newly identified markers or previously unmeasurable analytes for as long as this is technically possible and legally permissible.
This long-term storage only takes place with the written consent of the patient, who has been informed and informed accordingly beforehand. The patient transfers the biomaterials to the Center for Integrated Dementia Research (CIDC) at the University of Greifswald for the purpose of long-term storage and later use.
The samples are analyzed together with the Rostock sub-site and other partners within and outside the DZNE. The samples are accessed according to a transparent and standardized allocation procedure under the responsibility of the Institute for Community Medicine at the University of Greifswald.

4.6 Quality assurance and Quality control

The aim for this study is to achieve a similarly high quality standard as that established for the SHIP (Study of Health in Pomerania) in Greifswald. Binding manuals (Standard Operating Procedures - SOP) will be developed for all parts of the study.
The interviews are conducted by study staff who have been specially trained in conducting quality-assured patient interviews. The study investigators are employees of the University of Greifswald and the German Center for Neurodgenerative Diseases. The employees are bound by a data protection directive and are sworn to comply with the regulations of the Mecklenburg-Vorpommern State Data Protection Act. Descriptive statistics and plausibility checks are recorded at annual intervals and evaluated together with the project managers and the investigators. Quality assurance of data collection is ensured in cooperation with the Institute for Community Medicine (Individualized Medicine Project Group).
An external body for quality assurance was created with the establishment of a scientific advisory board on December 7, 2010, which meets at least once a year.

4.7 Data management

For data collection and processing, already established IT resources and structures of the Institute of Community Medicine will be used as far as possible. The strict guidelines on institutional data protection in accordance with the ICM's framework data protection concept are also adopted (see appendix).

The subject-related data collected during the visit to the GP will be documented on paper, entered into a central database and then permanently stored in filing cabinets to which only study staff have access. This procedure enables a timely quality and plausibility check corresponding to the data collection as well as the scientific evaluation of the transferred data.
The data collected during the survey is usually entered and stored directly in the study information system on mobile IT systems (notebooks, tablet PCs). The database is physically stored on a server of the Institute of Community Medicine, which is located in the externally closed medical network of the University Hospital Greifswald. Data is transferred between mobile IT systems and the server via secure VPN connections. The security standards set out in the framework concept for data protection and data security apply (see appendix).

5 Survey methods and instruments

5.1 Interview

The computer-assisted personal baseline interview is conducted by DelpHi interviewers after extensive training. It comprises the validated and commonly used measurement instruments shown in Table 2.

5.2 Blood sampling

An SOP is created for blood sampling at home, which defines the sampling and logistics in detail. This is based on the usual procedures in GANI_MED and SHiP-Home.

5.3 Secondary data

The respondent is asked to consent to the transmission of treatment data stored by the family doctor. This data is required to validate the respondent's information on diagnoses and medical treatments. As this data is restricted to the family doctor, the respondent is also asked to sign a confidentiality release for treatment data from the health insurance company. This data enables a comprehensive economic analysis of the intervention.

5.4 Invasiveness

Blood sampling is slightly invasive. The study applied for is an intervention study that does not represent a medical-therapeutic intervention within the meaning of the AMG or the Medical Devices Act. Neither drugs nor medical devices will be tested. All procedures to be

used in the context of care optimization have already been approved, validated for the intended purpose and established in other studies.
It is not to be expected that participation in the study could pose a risk to the test subjects. This is a study with voluntary participation on the basis of comprehensive informed consent. No further invasive medical interventions are planned; there will be no additional medical diagnostics in the Greifswald part of the study that could indicate an intervention. A random sample of participants will be invited to undergo in-depth diagnostics (imaging, biomarkers). These diagnostics will be performed at the Rostock study center. Separate information is provided for the validation studies and separate ethics concepts are submitted.
In the following parts of the Greifswald study, side effects (usually very minor) may occur:

Blood sampling

- Hematomas, pain

5.4.1 Quality assurance

5.4.1.1 Standardization

The methods are described before the start of the study in a standard operating procedure (SOP), which must be read and followed by all employees involved in the study. This ensures standardization of the study procedures.

5.4.1.2 Training, Certification

All interviewers are trained at the Institute of Community Medicine and the cooperating partners and take part in regular training sessions based on the SHIP procedure.
The DCMs take part in a weekly case discussion in which sample and problem cases from the intervention group are described. This serves the purpose of professional development, collegial exchange and the joint resolution of difficulties arising in the project. Psychiatrists, psychologists, pharmacologists and epidemiologists take part in these case discussions in order to ensure an interdisciplinary solution to any problems that arise.

6 Subject information and informed consent

6.1 Informing the subjects about the objectives and procedure of the study

Before carrying out the screening, the GP informs the patient that the tests also serve as inclusion criteria for the DelpHi-MV study if a certain result is achieved. The doctor provides the subject with study information (see Appendix 11.1, 11.2) with a brief explanation of the

study procedure and the names of the contact persons at the CIDC. He asks whether the subject agrees that the results of the screening may be forwarded to the study team for evaluation, stating his name, age, address and telephone number. If he/she agrees, the proband signs a declaration of consent in which he/she (a) agrees to the forwarding of personal address data, (b) agrees to the forwarding of the results of his/her screening test to the study team, (c) releases the doctor from his/her duty of confidentiality towards the study team and (d) allows the study team to contact him/her (see Annexes 11.3, 11.4, 11.4).

The doctor then forwards the personal data, the results of the screening test and the declaration of consent to the responsible study team. In addition, before the start of the screening phase, the GPs involved in the project receive training on how to carry out the DemTect and information about the study, as well as an SOP (Standard Operating Procedure) for carrying out the screening. The doctor carrying out the screening receives an expense allowance of €10 for each screening. For each participant who fulfills the inclusion criteria and participates in the study, the physician receives an expense allowance of €100.

If the respondent tests positive for suspected dementia and meets the other inclusion criteria specified above, the respondent is included in the DelpHi-MV. On the basis of the recruiting GP practice, the respondent is initially assigned to the intervention or control group. This assignment is based on the status of the GP involved in the project. In the control group, the test subjects are treated according to the usual medical procedures (usual care). In the intervention group, the test person receives care within the network in accordance with the individually agreed intervention pathways.

As, in addition to assessing the patient's situation, a further aim of the care analysis is to assess the burden on relatives, the main caregiver of each test person is also included in the study.

In the intervention group, the DCM is responsible for interviewing the subjects at home and the supporting/caregiving relative is interviewed by trained study staff. In the control group, study staff will conduct both the subject and relative interviews.

6.2 Declaration of consent, Capacity to consent

It can normally be assumed that patients who visit their GP have full capacity to give consent. If they are not capable of giving consent, it can be assumed that the GP is aware of this due to many years of contact, or that the patient is accompanied by a carer. The GP may assume that the patient has limited capacity to consent, but the ultimate certainty must be determined by an expert.

In the context of the GP visit, the assessment of capacity is secondary because the aim of the GP is to provide treatment that is subject to different rules with regard to the patient's consent, such as a scientific study with people suffering from dementia.

In the DelpHi-MV study, subjects who are unable to give consent are treated and their capacity to give consent is assessed as follows. In which cases the patient's consent is sufficient is at the discretion of the general practitioner. If the patient already has a caregiver or authorized representative and the family doctor is aware of this, the declaration of consent must be obtained from them. If the patient is accompanied by a relative, the doctor is required to obtain their consent. This also applies in view of the fact that the relative may themselves become a subject in the study.

This approach is pragmatic, but not without ethical concerns and must therefore be treated in a more differentiated manner. On the whole, 2 considerations must be taken into account. On the one hand, the assessment of capacity to consent by the family doctor is not sufficient for the long-term perspective of the study; on the other hand, the inclusion of the relative may violate the right to self-determination.

The problem of capacity to consent is a special case in the context of the DelpHi-MV study, which has no analogous equivalent in SHIP or GaniMed, for example. Part of the nature of dementia is that the ability to assess the consequences of one's own actions is increasingly partially or completely lost as the disease progresses. The screening will therefore recruit both people who are unable to assess all aspects of the consequences of their decision and those who are able to do so at the time.

In routine care, this problem is less serious, as the visit to the GP is normally for medical treatment and the decision on the ability to give consent is at the doctor's discretion. The situation is different in the context of a scientific study. If there is legal guardianship, a written declaration of consent from the legal representative must be available in order to include the test person in the study. If there is no legal guardianship, the subject must be informed of the consequences of participating in the study. This includes the fact that their inclusion is based on the suspicion of dementia.

As recognized screening procedures are used, which are also used in routine clinical practice to identify dementia, the subject must be informed that further steps will be taken to clarify the suspicion. This may mean that the first screening may have produced a false positive result, which initially led to inclusion in the study, or that dementia will be diagnosed. Clarification of the diagnosis is the responsibility of the general practitioner, as they must also investigate the suspicion outside of the DelpHi-MV study as part of their routine care. If the suspicion of the presence of dementia in the test person in question is confirmed, there is a need for action on the part of the doctor. He or she will provide information about this at an early stage and also initiate that the test person seeks legal representation. If a guardian has been appointed, a written declaration of consent must be obtained from the legal representative for the subject's continued participation (subsequent consent). This means that the study team is obliged to clarify the status of the legal representation of the individual

subjects with the family doctor prior to contacting them. This is logistically feasible because the DCMs supervise the GP practices and are regularly on site. The procedure is illustrated graphically in Figure 4.

In reality, it will be the case that a positive screening will not lead to the appropriate steps being taken. We have no way of influencing this. Nor can we yet estimate how large this group will be. The greatest influence we can have is through appropriate training of GPs, which is planned anyway as part of the activities of the CIDC and DZNE. In addition, it is planned that the participating GPs will be provided with the guidelines for the treatment of people suffering from dementia.

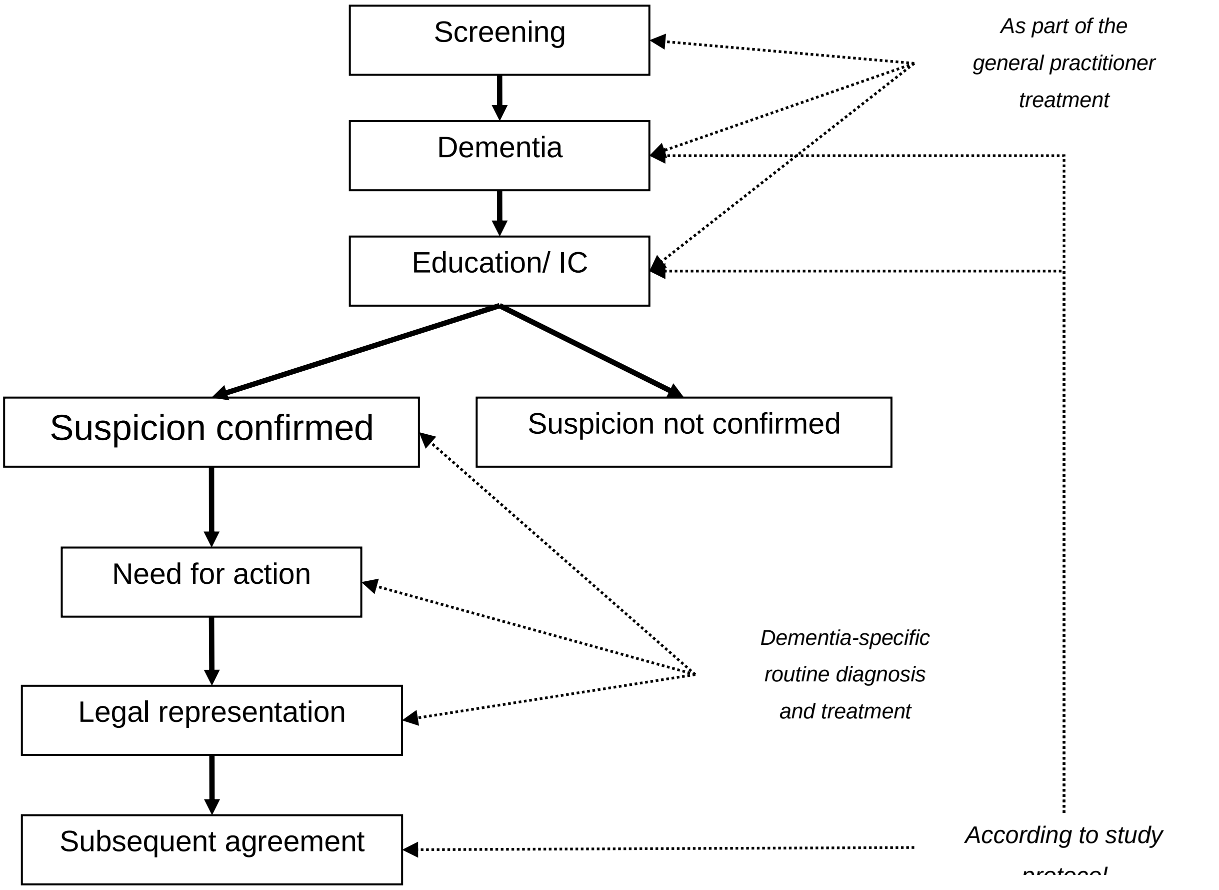

Figure 4: Flowchart written declaration of consent (IC) and subsequent consent

The inclusion of the subject's relatives poses a further challenge. A separate declaration of consent is required for this (see Appendix 11.4), because under certain circumstances the patient's right to self-determination could be restricted. As part of this, the subject must be informed that the next of kin may learn limited diagnoses and other medical information about the subject. Even if one aim of the study is to include the next of kin and this should lead to an improvement in the subject's situation, the subject must also be able to participate

without the participation of a next of kin. This problem is explained in detail in the participant information.

In general, the study design is such that the group benefit of the scientific findings is in the foreground and legitimate. The integration of add-on studies should be uncritical, analogous to Gani_Med. For their integration, ethics concepts will be submitted that relate to the specifics of the recruitment envisaged in the DelpHi-MV (no clinical population, specific participant information, etc...).

The corresponding declarations of consent are attached to this application.

6.3 Exceptions to obtaining consent

There are no exceptions.

6.4 Extension of the research question

The follow-up of test subjects is essential for cohort studies and intervention studies. As already explained for the follow-up studies under 4.2, subjects will only be contacted or interviewed by study personnel. We plan to continue to write to the test subjects' treating physicians and their health insurance companies. Patients will also give their written consent to this (see declaration of consent in the appendix).

The preservation of biosamples also serves the future expansion of questions. The subjects are asked for their consent (see Appendix 11.6).

7 Confidential Treatment of data

7.1 Type and scope of encryption

Personal data is collected that is used to (a) organize contact with the test subjects and (b) link the data to the individual participants via the various survey types and times of the survey and (c) assign the data to the test subjects and their supporting relatives/persons. The subjects are informed about this and asked for their written consent.

The data collected as part of the screening, the baseline study, the intervention and the follow-ups are pseudonymized and stored separately from the personal data in accordance with the data protection concept. The scientific analyses are pseudonymized. Only anonymized and aggregated data are published, which do not allow any conclusions to be drawn about the individual person.

The following data protection precautions are taken:
The documents containing the personal data are only accessible to the authorized employees of the DelpHi-MV study. All other data will be kept by the DCM or the trained interviewers in a study database specially developed for this purpose. The personal and results data required for the home visits to be carried out on a particular day are transferred promptly from the study server to the tablet PC. At the end of the day, the data is transferred back to the study server, where it is encrypted and stored. Once the data has been uploaded, there is no more patient data on the tablet PC.
Data entry is carried out exclusively by authorized members of the study teams via a VPN connection (Virtual Private Network) to a database located on a CIDC server. This is located within the medical network of the University Hospital Greifswald. The PCs are configured in such a way that no other connections to the Internet are possible. The data entered is therefore promptly available to the CIDC for quality and completeness checks and for evaluation. At the same time, the completed interview forms of the test subjects and caregiving relatives are stored in separately locked filing cabinets in the archive room of the CIDC in accordance with the guidelines of good epidemiological practice. [44]
Both the survey software and the study software are protected by a password from the respective DCM. The tablet PC is protected by the Windows firewall and the McAfee program is used for virus protection. The data is stored in encrypted form in the survey software. Only encrypted data is stored on the hard disk at any time. Personal identifying data can only be viewed exclusively by the DCM via a special view. When the evaluation data is created, the personally identifiable data is removed from the data set and only the pseudonymized patient IDs are available to the study staff.
The use and forwarding of the medication data to the pharmacy selected by the patient is secured by the patient's data use consent in accordance with Section 4a of the Federal Data Protection Act.

8 Legal relationships

8.1 Subject insurance

For this study, no subject insurance is required under the German Drug Act or Medical Devices Act. However, a subject insurance for clinical trials not subject to the obligation to provide cover (ProbV-NV) is being sought. We have requested offers for such insurance from 3 insurance companies (as of February 11, 2011; Allianz, HDI, AXA).

8.2 Patient-fee

The patients do not receive any compensation.

8.3 Reimbursement of expenses

The participating GPs receive an expense allowance of €10 per screened patient and €100 per successfully recruited test person.

9 Biological materials / Genetic Marker

9.1 Type of obtained materials

Blood is taken. Parts of the blood samples are stored frozen in aliquots. DNA is extracted from the blood samples and also stored frozen.

9.2 Declaration of consent

The test subjects are informed in detail about the collection and storage of the biosamples and their consent is obtained. If consent is not obtained, no material will be collected. Participation in the study is independent of consent to blood sampling.

9.3 Subsequent expansion of the research question and recourse to existing materials

The purpose of storing the biosamples is to be able to access them later, also with regard to an extension of the questions (future research). This applies, for example, to genetic markers of serum markers, which can only be determined once the study population has been completed.

9.4 Notification of findings

The results of genetic and other low informative (i.e. from a medical point of view for quality of life, progression, therapy or diagnostics. The APoE status mentioned above, for example, is important for the differentiation of dementia, as it represents a risk factor, especially for Alzheimer's dementia. However, no treatments can be derived from this knowledge [45].

10 Communication of the Results

10.1 Scientific Publication

The DelpHi-MV study is conducted with the primary aim of generating and publishing scientific findings. Individual, subject-related results of investigations are not published.

10.2 Information of Test Subjects, Relatives, GPs

The data collected in the study will be disclosed to the test subjects on request. The right to self-determination, including the right not to know, is respected: subjects who do not wish to receive such information will not receive it.

One exception concerns cases in which self-harm cannot be ruled out (e.g. suicidal tendencies). Only in these cases will the GP be informed of the relevant findings.

Patient data is only collected on the basis of an existing patient authorization. Data queries (general practitioners and specialists, health and long-term care insurance companies as well as the Association of Statutory Health Insurance Physicians, register of residents) are only carried out on the basis of an existing authorization to contact selected persons and healthcare facilities (release from confidentiality vis-à-vis the DelpHi-MV study management). This information is only accessed in pseudonymized form for the purposes of analysis.

10.3 Communication to decision-makers, general public

Population-relevant (disease-relevant) results of the study are communicated to the non-scientific public in an appropriate form. Individual, subject-related results of studies are not published.

11 Attachments

11.1 Participant information for test subjects with suspected dementia

11.2 Participant information Respondent without cognitive impairment

11.3 Declaration Test Subject

11.4 Declaration of consent by relatives of the test person

11.5 Declaration of consent for subjects without cognitive impairments

Reference List

(1) Fendrich K, Hoffmann W. More than just aging societies: The demographic change has an impact on actual numbers of patients. J Public Health 2007; 15(5):345-351.

(2) Bickel H. Die Epidemiologie der Demenz. Deutsche Alzheimer Gesellschaft e.V., editor. 1, 1-3. 2008. Berlin. Das Wichtigste - Informationsblatt der Deutschen Alzheimer Gesellschaft e.V. 11-7-2008.
Ref Type: Serial (Book,Monograph)

(3) Bickel H. Demenzsyndrom und Alzheimer Krankheit: Eine Schätzung des Krankenbestandes und der jährlichen Neuerkrankungen in Deutschland. Gesundheitswesen 2000; 62(4):211-218.

(4) Institut für Qualität und Wirtschaftlichkeit im Gesundheitswesen. Abschlussbericht A05-19A: Cholinesterasehemmer bei Alzheimer Demenz. 10-4-2007.
Ref Type: Report

(5) Schulz R, Visintainer P, Williamson GM. Psychiatric and physical morbidity effects of caregiving. J Gerontol 1990; 45(5):181-191.

(6) McCallion P, Toseland RW, Freeman K. An evaluation of a family visit education program. J Am Geriatr Soc 1999; 47(2):203-214.

(7) Mitrani VB, Czaja SJ. Family-based therapy for dementia caregivers: clinical observations. Aging Ment Health 2000; 4(3):200-209.

(8) Gwyther LP. Social issues of the Alzheimer's patient and family. Am J Med 1998; 104(4A):17S-21S.

(9) Belle SH, Burgio L, Burns R, Coon D, Czaja SJ, Gallagher-Thompson D et al. Enhancing the quality of life of dementia caregivers from different ethnic or racial groups: a randomized, controlled trial. Ann Intern Med 2006; 145(10):727-738.

(10) Reuben D, Levin J, Frank J, Hirsch S, McCreath H, Roth C et al. Closing the dementia care gap: Can referral to Alzheimer's Association chapters help? Alzheimers Dement 2009; 5(6):498-502.

(11) Etters L, Goodall D, Harrison BE. Caregiver burden among dementia patient caregivers: a review of the literature. J Am Acad Nurse Pract 2008; 20(8):423-428.

(12) Etters L, Goodall D, Harrison BE. Caregiver burden among dementia patient caregivers: a review of the literature. J Am Acad Nurse Pract 2008; 20(8):423-428.

(13) Thompson C, Spilsbury K. WITHDRAWN: Support for carers of people with Alzheimer's type dementia. Cochrane Database Syst Rev 1998;(3):CD000454.

(14) Thompson CA, Spilsbury K, Hall J, Birks Y, Barnes C, Adamson J. Systematic review of information and support interventions for caregivers of people with dementia. BMC Geriatr 2007; 7:18.

(15) Dietl M, Kornhuber J, Schoffski O, Grassel E. [Cost-effectiveness model of a community-based service for dementia caregivers]. Gesundheitswesen 2010; 72(2):99-105.

(16) Donath C, Luttenberger K, Grassel E. [Dementia caregiver skill training--predictors for utilisation and expected quality from the family caregiver's point of view]. Gesundheitswesen 2009; 71(5):291-292.

(17) Terschüren C, Fendrich K, van den Berg N, Hoffmann W. Implementing telemonitoring in the daily routine of a GP practice in a rural setting in northern Germany. J Telemed Telecare 2007; 13(4):197-201.

(18) van den Berg N, Kleinke S, Heymann R, Oppermann RF, Jakobi B, Hoffmann W. Transfer of the AGnES Concept to the Regular German Health-Care System: Legal Evaluation, Reimbursement, Qualification. Gesundheitswesen 2010; 72:285-292.

(19) van den Berg N, Fiss T, Meinke C, Heymann R, Scriba S, Hoffmann W. GP-support by means of AGnES-practice assistants and the use of telecare devices in a sparsely populated region in Northern Germany - proof of concept. BMC Fam Pract 2009;1-8.

(20) Dreier A, Rogalski H, Oppermann RF, Terschueren C, van den Berg N, Hoffmann W. A curriculum for nurses in Germany undertaking medically-delegated tasks in primary care. J Adv Nurs 2010; 66(3):635-644.

(21) Fiss T, Ritter CA, Alte D, van den Berg N, Hoffmann W. Detection of drug related problems in an interdisciplinary health care model for rural areas in Germany. Pharm World Sci 2010; in press.

(22) Kalbe E, Kessler J, Calabrese P, Smith R, Passmore AP, Brand M et al. DemTect: a new, sensitive cognitive screening test to support the diagnosis of mild cognitive impairment and early dementia. Int J Geriatr Psychiatry 2004; 19(2):136-143.

(23) Kalbe E, Brand M. Der DemTect in der klinischen Anwendung: Sensitivität und Spezifität eines kognitiven Screeninginstruments. Zeitschrift für Gerontologie & -psychiatrie 2005; 18(3):121-130.

(24) Monsch AU. CERAD. Neuropsychologische Testbatterie. Universität Basel; 1997.

(25) Yesavage JA, Brink TL, Rose TL, Lum O, Huang V, Adey M et al. Development and validation of a geriatric depression screening scale: a preliminary report. J Psychiatr Res 1982; 17(1):37-49.

(26) Luck T, Busse A, Hensel A, Angermeyer MC, Riedel-Heller SG. [Mild cognitive impairment and development of dementia]. Psychiatr Prax 2008; 35(7):331-336.

(27) Riedel-Heller SG, Busse A, Aurich C, Matschinger H, Angermeyer MC. Prevalence of dementia according to DSM-III-R and ICD-10: results of the Leipzig Longitudinal Study of the Aged (LEILA75+) Part 1. Br J Psychiatry 2001; 179:250-254.

(28) Ehreke L, Luppa M, Luck T, Wiese B, Weyerer S, Eifflaender-Gorfer S et al. Is the clock drawing test appropriate for screening for mild cognitive impairment?--Results of the German study on Ageing, Cognition and Dementia in Primary Care Patients (AgeCoDe). Dement Geriatr Cogn Disord 2009; 28(4):365-372.

(29) Sunderland T, Hill JL, Mellow AM, Lawlor BA, Gundersheimer J, Newhouse PA et al. Clock drawing in Alzheimer's disease. A novel measure of dementia severity. J Am Geriatr Soc 1989; 37(8):725-729.

(30) Bullinger M, Kirchberger I. SF-36 Fragenbogen zum Gesundheitszustand. Göttingen 1998: Hogrefe-Verlag GmBH & Co.KG; 1998.

(31) Cummings JL. The Neuropsychiatric Inventory: assessing psychopathology in dementia patients. Neurology 1997; 48(5 Suppl 6):S10-S16.

(32) Erzigkeit H, Lehfeld H, Pena-Casanova J, Bieber F, Yekrangi-Hartmann C, Rupp M et al. The Bayer-Activities of Daily Living Scale (B-ADL): results from a validation study in three European countries. Dement Geriatr Cogn Disord 2001; 12(5):348-358.

(33) Hindmarch I, Lehfeld H, de JP, Erzigkeit H. The Bayer Activities of Daily Living Scale (B-ADL). Dement Geriatr Cogn Disord 1998; 9 Suppl 2:20-26.

(34) Galasko D, Bennett D, Sano M, Ernesto C, Thomas R, Grundman M et al. An inventory to assess activities of daily living for clinical trials in Alzheimer's disease. The Alzheimer's Disease Cooperative Study. Alzheimer Dis Assoc Disord 1997; 11 Suppl 2:S33-S39.

(35) Logsdon RG, Gibbons LE, McCurry SM, Teri L. Assessing quality of life in older adults with cognitive impairment. Psychosom Med 2002; 64(3):510-519.

(36) Fydrich T, Sommer G, Brähler E. F-SozU. Fragebogen zur sozialen Unterstützung. Göttingen: Hogrefe; 2007.

(37) Zank S, Schacke C, Leipold B. Berliner Inventar zur Angehörigenbelastung - Demenz (BIZA-D). Zeitschrift für Klinische Psychologie und Psychotherapie 2006; 35(4):296-305.

(38) Derogatis LR, Melisaratos N. The Brief Symptom Inventory: an introductory report. Psychol Med 1983; 13(3):595-605.

(39) Löwe B, Zipfel S, Herzog W. PHQ-D Gesundheitsfragebogen für Patienten. Pfizer; 2002.

(40) Spitzer RL, Kroenke K, Williams JB. Validation and utility of a self-report version of PRIME-MD: the PHQ primary care study. Primary Care Evaluation of Mental Disorders. Patient Health Questionnaire. JAMA 1999; 282(18):1737-1744.

(41) Spitzer P, Klafki H, Blennow K, Buée L, Esselmann H, Herruka S et al. cNEUPRO: Novel Biomarkers for Neurodegenerative Diseases. Int J Alzheimers Dis 2010;(Article ID 548145).

(42) Sturgeon C. Perspectives in Clinical Proteomics Conference: translating clinical proteomics into clinical practice. Expert Rev Proteomics 2010; 7(4):469-471.

(43) Guhne U, Matschinger H, Angermeyer MC, Riedel-Heller SG. Incident dementia cases and mortality. Results of the leipzig Longitudinal Study of the Aged (LEILA75+). Dement Geriatr Cogn Disord 2006; 22(3):185-193.

(44) Hoffmann W, Latza U, Terschüren C. Leitlinien und Empfehlungen zur Sicherung von Guter Epidemiologischer Praxis (GEP) - überarbeitete Fassung nach Evaluation. Gesundheitswesen 2005; 67(3):217-225.

(45) Deuschl G, Reichmann H. Gerontoneurologie. Stuttgart: Thieme; 2010.

Statistical Analyses Plan for the Primary Efficacy Analyses in the DelpHi trial

Johannes Hertel*

March, 2016 (final editing November, 2016)

**Address for correspondence:*
Johannes Hertel
Department of Psychiatry, University Medicine Greifswald, Germany
Ellernholzstrasse 1-2, 17475 Greifswald
Tel.: ++49 – (0)3834/ 86 22 1 66; Fax.:++49 – (0)3834/86 68 89
e-mail: hertelj@uni-greifswald.de

0 Preamble

Summary of the trial´s protocol

This statistical analysis refers to the “Dementia: life- and person-centered help in Mecklenburg-Western-Pommerania (DelpHi)”-study. The study protocol submitted to the ethics committee and the local IRB was written in german. However, the paper “Life- and person-centred help in Mecklenburg-Western Pomerania, Germany (DelpHi): study protocol for a randomised controlled trial” (Thyrian et al, Trials. 2012 May 10;13:56. doi: 10.1186/1745-6215-13-56) provides an english summary of the protocol and is open access. A brief summary is given here.

The study plan was designed to assemble a general physician-based epidemiological cohort of people above the age of 70 who live at home (DelpHi cohort). These people are systematically screened for eligibility to participate in a trial of dementia care management (DelpHi trial, eligibility criteria: age 70+. living at home, dementia screening positive). The trial is a complex, cluster-randomised, controlled intervention trial with two arms (intervention and control), assessments at baseline and yearly follow-up. The study was designed to test the efficacy and efficiency of implementing a subsidiary support system for persons with dementia who live at home. This subsidiary support system is initiated and coordinated by a dementia care manager: a nurse with dementia-specific qualifications who delivers the intervention according to a systematic, detailed protocol. The intervention starts right after baseline assessment and is planned to last 6 months. The follow-up assessment that is planned for the evaluation of efficacy and efficiency is planned 12 months after baseline assessment.

The primary outcomes in this complex intervention trial are quality of life and healthcare for patients with dementia and their caregivers. These are multidimensional outcomes with a

focus on four dimensions: (1) quality of life, (2) caregiver burden, (3) behavioural and psychological symptoms of dementia and (4) pharmacotherapy with an antidementia drug and prevention or suspension of PIM. Quality of life will be measured using the Quality of Life in Alzheimer's Disease instrument, which consists of 13 items and includes "assessments of the individual's relationships with friends and family, concerns about finances, physical condition, mood, and an overall assessment of life quality" . To measure caregiver burden, the Berliner Inventar zur Angehörigenbelastung (BIZA-D) will be used. BIZA-D is a standardised, theoretically grounded, psychometrically validated instrument used to assess burden and stress. It covers physical exhaustion, personal restrictions in life, missing social appreciation, personal development and negative appraisal. It also assesses tasks required in caring for the person with dementia, in motivating and guiding, in supporting in care, in emotional support and in oversight. The Neuropsychiatric Inventory (NPI), developed by the Alzheimer's Disease Cooperative Study investigators, is used as a standardised instru instrument to assess behavioural and psychological symptoms. The assessment of pharmacotherapy with antidementia drugs focuses on the following substances which are approved by the drug authorities and recommended by the current guidelines: donepezil, galantamine, rivastigmine and memantine. Additionally, we evaluate the reduction of PIM according to the PRISCUS criteria as well as the reduction of anticholinergic drugs. The following are the secondary outcomes: The Structured Interview for the Diagnosis of Dementia, or SIDAM, will serve to identify screening false-positives as well as to differentiate different syndromes of dementia. Activities of daily living will be assessed using the Bayer Activities of Daily Living Scale, or B-ADL. 3. Social support will be assessed using the Social Support Questionnaire (F-SozU). 4. Health status will be assessed using several instruments that measure health-related variables of

the person with dementia, including the GP records, the SF-12 Health Survey, the standardized assessment for elderly patients in a primary care setting (STEP), the Brief Symptom Inventory (BSI) and the Gesundheitsfragebogen für Patienten (PHQ-D). 5. Utilisation of health care resources will be assessed according to GP and specialist visits, outpatient treatments, inpatient treatments, hospitalisations, nursing home admissions, therapeutic appliances and provision of informal care. 6. Medications will be assessed by the DCM, who will conduct an information technology-supported standardised home medication review at the patient's home with subsequent medication management by the patient's local pharmacy regarding the frequency of drug-related problems, intake of PIMs, clinically relevant drug-drug interactions, adherence, adherence to supportive activities (that is, medication plan, drug dispenser, support by care service, reduction of the number of drugs taken and home medication review).

1 Preliminary Remarks for the statistical analysis plan

The statistical analyses plan was written in its first version in November/Dezember 2015 and was then finalized in March 2016. The follow-up data was analyzed beginning April 2016 until October 2016. Thus, the analysis plan was written before analyzing the follow-up data. However, the baseline data was analyzed since 2013 and the design paper was published in 2011. At the time of the publication of the design paper, the study statistician (Johannes Hertel) was not yet employed in the DZNE and was therefore neither involved in designing the DelpHi-MV trial, nor in writing the design paper. The statistical analyses plan therefore is not coherent with the design paper in several points. The statistical analyses plan here only describes the efficacy analyses regarding the primary endpoints of the DelpHi trial.

The DelpHi cohort consists of individuals (above 70 years) who were screened positively on dementia. The sampling was done in primary care in the general practice (GP). Additionally, if possible, the principle care giver was included into the study. The GP was randomized by fair coin tossing to care

as usual or intervention group. Thus, DelpHi is a cluster randomized clinical trial and all analyses have to respect the stochastic dependency of the data on the GP clustering. The randomization was done before baseline assessment of the individuals and the intervention cannot be classified as blinded, neither on the level of the GP, nor on the level of the study participant. The DelpHi trial can be classified as exploratory as no effect sizes a priori were known for the complex intervention that was performed. Moreover, the trial is clearly a pragmatic trial as it is implemented in primary care. Thus, the internal validity is potentially limited and several sources of bias (especially selection, attrition and performance bias) bias have to be assessed.

The power calculations mentioned in the design paper did not reflect the clustering of the data. The power of cluster randomized trials is not only a function of effect size, significance level and sample size, but dependent on the parameters of the concrete parameters of the clustering which were not known before sampling the data. In general, the power of a cluster randomized trial is lower in comparison to a randomized trial of equal sample size. The concrete loss of power is a function of the intra-class correlation (ICC) of the outcome, the number and size of the clusters and the variability of the cluster sizes in the sample. It is known that with higher ICC the statistical power gets lower. If the ICC would be zero, normal power calculations apply. Thus, the sample size calculations noted in the design paper can be seen as upper bounds assuming that the ICC is zero for all outcome parameters. More accurate power calculations were therefore performed during the baseline sampling when first estimates of the ICCs where known and can be found at the end of this document.

2 Analysis Plan

2.1 Definition of the Primary Efficacy Endpoints

The design paper defined four dimensions of primary endpoints:

(1) Quality of life

(2) Caregiver burden

(3) Behavioural and psychological symptoms of dementia

(4) Pharmacotherapy with an antidementia drugs and prevention or suspension of potentially inadequate medication (PIM)

The fourth dimension includes two outcome measurements (antidementia drug treatment) and the suspension of PIM, thus, in total five outcome variables are derived. The first follow-up values on these dimensions (12 months after baseline) were defined as efficacy endpoints.

2.2 Operationalization of the Primary Endpoints

(1) Quality of life

Quality of life was measured using the Quality of Life in Alzheimer's Disease instrument [1]. It consists of 13 Lickert scale items that covers the following dimensions: physical health, energy level, mood, living situation, relationship with family members, caregivers and friends, memory as well as the ability to meaningful activities and financial situations. This instrument has been shown reliable and valid ratings by persons with mild to moderate dementia. Higher scores indicated better quality of life. The mean response on all answered items is used as outcome measurement, regardless whether the response on single items is missing. The mean response is treated as an interval scaled metric outcome.

(2) Caregiver burden

Caregiver burden was measured via the Berliner Inventar zur Angehörigenbelastung (BIZA-D) [2]. The BIZA-D was developed to assess objective and subjective burden due to caring for a PWD. It consists of 88 items covering 20 dimensions of caregiver burden. Objective burden is divided into six dimensions, assessed by 25 items: 1) basic care tasks, such as supporting eating and hygiene (seven items), 2) extended care tasks, such as supporting grocery shopping and legal affairs (three items), 3) motivation and guidance (four items), 4) emotional support (four items), 5) support of the maintenance of social contacts (3 items) and 6) supervision (four items). Each item has to be rated regarding the frequency of the support needed on a 5-point scale (e.g., supervision: Does the patient need this type of support 1=always, 2=mostly, 3=partly, 4=hardly, 5=not at all). Subjective burden is divided into a) the subjective burden of behavior change (six dimensions: burden due to cognition with four items, aggression with five items, depression with four items, disorientation with five items, late symptoms with three items, and loss of relationship with five items); b) the subjective burden of perceived conflicts between needs and responsibilities to care (six dimensions: burden due to personal constraints with nine items, negative evaluation of one's own caring with four items, missing social appreciation with four items, financial losses with four items, personal development with three items, missing institutional support with three items); and c) role conflicts (two dimensions; professional role conflicts with four items, family role conflicts with five items).

From analyzing the baseline data, it was known that the 20 sum scores of the scales were highly intercorrelated with each other. Consequently, in a principle component analysis, one principle component explaining 38.6% of variance dominated. As the sequential analyses of each scale would lead to massive multiple testing, the first principle component of the BIZA-D is chosen as outcome parameter, representing a compound measure of the caregiver burden. This compound measurement is treated as an interval scaled metric outcome.

(3) Behavioral and psychological symptoms of dementia

(2) Caregiver burden

Caregiver burden was measured via the Berliner Inventar zur Angehörigenbelastung (BIZA-D) [2]. The BIZA-D was developed to assess objective and subjective burden due to caring for a PWD. It consists of 88 items covering 20 dimensions of caregiver burden. Objective burden is divided into six dimensions, assessed by 25 items: 1) basic care tasks, such as supporting eating and hygiene (seven items), 2) extended care tasks, such as supporting grocery shopping and legal affairs (three items), 3) motivation and guidance (four items), 4) emotional support (four items), 5) support of the maintenance of social contacts (3 items) and 6) supervision (four items). Each item has to be rated regarding the frequency of the support needed on a 5-point scale (e.g., supervision: Does the patient need this type of support 1=always, 2=mostly, 3=partly, 4=hardly, 5=not at all). Subjective burden is divided into a) the subjective burden of behavior change (six dimensions: burden due to cognition with four items, aggression with five items, depression with four items, disorientation with five items, late symptoms with three items, and loss of relationship with five items); b) the subjective burden of perceived conflicts between needs and responsibilities to care (six dimensions: burden due to personal constraints with nine items, negative evaluation of one's own caring with four items, missing social appreciation with four items, financial losses with four items, personal development with three items, missing institutional support with three items); and c) role conflicts (two dimensions; professional role conflicts with four items, family role conflicts with five items).

From analyzing the baseline data, it was known that the 20 sum scores of the scales were highly intercorrelated with each other. Consequently, in a principle component analysis, one principle component explaining 38.6% of variance dominated. As the sequential analyses of each scale would lead to massive multiple testing, the first principle component of the BIZA-D is chosen as outcome parameter, representing a compound measure of the caregiver burden. This compound measurement is treated as an interval scaled metric outcome.

(3) Behavioral and psychological symptoms of dementia

The behavioral and psychological symptoms of dementia were operationalized by the Neuropsychiatric Inventory (NPI), developed by the Alzheimer's Disease Cooperative Study investigators [3]. The NPI represents an interview by proxy on twelve dimensions of neuropsychiatric behaviors, i.e. delusions, hallucinations, agitation, dysphoria, anxiety, apathy, irritability, euphoria, disinhibition, aberrant motor behavior, night-time behavior disturbances, and appetite and eating abnormalities. The severity and frequency of each neuropsychiatric symptom are rated on the basis of scripted questions administered to the patient's caregiver. A total NPI score is calculated as the product sum of the frequency by severity scores within each domain. The NPI also assesses the amount of caregiver distress engendered by each of the neuropsychiatric disorders, but the caregiver distress scores were not used in our analysis. The total score of all twelve scales were used as interval scale metric outcome variable as supposed in the NPI manual.

(4) Pharmacotherapy with an antidementia drugs

Antidementia drug treatment was defined as in Wucherer et al. [4] The computer-based collection of primary data on medication in the context of the home medication review includes both prescription drugs (Rx) and over-the-counter (OTC) drugs. The assignment was integrated using a master file of the Pharmaceutical Index (GKV-Arzneimittelindex) [5]. Active substances were coded according to the Anatomical Therapeutic Chemical (ATC) classification system (German Modification)[6]. The outcome variable is dichotomous with individuals taking one of the following drugs (N06DA02: donepezil; N06DA03: rivastigmine; N06DA04: galantamine; N06DA52: donepezil and memantine; N06DX01: memantine) coded with “1”. Individuals not taking one these drugs were coded with “0”.

(5) Prevention or suspension of potentially inadequate medication (PIM)

Suspension of PIM taking was defined according to Priscus list [7]. A list of all ATC codes considered as PIMs is given in the appendix. From the baseline analyses, it was known that multiple prescriptions of PIMs are seldom in the study population (Wucherer et al. 2016). Thus, for efficacy analyses, we dichotomize the PIM prescriptions: “1”= one or more PIMs prescribed; “0”= no PIM prescribed.

2.3 Efficacy Analyses

2.3.1 Per protocol analyses

2.3.1.1 Definition of the analyses set

We define the per protocol analysis as complete case analyses. The only reason not to be included into the per-protocol analysis is due to missing data in the baseline assessment or the follow-up assessment. Contaminations by changing treatment assignments after randomization are impossible due to the study design. The main reason for missing data is supposed to be drop-out because of death or withdrawal of informed consent.

2.3.1.2 Primary efficacy Analyses

For primary efficacy analyses, for each of the main outcome of the study a separate generalized linear mixed model is fitted with the model specification corresponding to the scale level of the outcome under investigation. Thus, for quality of life, caregiver burden and neuropsychiatric symptoms linear mixed regressions will be used assuming a Gaussian distribution, while for antidementive drug treatment and PIM prescription logistic mixed models will be used. To account for the stochastic dependency of patients treated by the same GP, the GP will be included as random effect variable.

The follow up outcome variable at T_1 (twelve months after finishing the baseline) will be the dependent variable in these analyses; the study group is the predictor of interest, while the baseline value (T_0) of the outcome variable will be included as covariate to diminish residual variance and to account for inter-individual variance at baseline. Furthermore, sociodemographic variables (age, sex and living situation (alone vs. not alone)) are planned to be included as covariates, too. These variables can be supposed to contribute proportions of variance to the outcome variables which should be independent of possible study effects as the intervention cannot change age, sex or living situation. Thus, the inclusion of these variables should result in decreased residual variance enhancing thereby

the statistical power to detect differences between study groups. A positive intervention effect is then defined as a significant regression coefficient (one-sided test) of the study group variable. The p-values will be interpreted one-sided as one-sided hypotheses are tested. The design of the DelpHi trial allows for multiple testing (five primary outcomes), therefore, additionally the Bonferoni corrected p-values will be reported.

2.3.1.3 Sensitivity Analyses

Including the GP as random effect variable in the equation using random intercepts only implies that the effect of the GP on the outcome variable is independent of the effects of the predictors which may not be correct for the study group variable and the baseline variable of the outcome. Thus, to test the robustness of the potential treatment effect, in sensitivity analyses random slopes for the baseline variable of the outcome will be introduced into the models. Descriptively, these models allow for GP dependent treatment effect and thus will result in treatment estimates respecting the possibility of differentially collaborating GPs, thus assessing the robustness of the results regardless of a potentially present performance bias.

The treatment effect estimates from the different models will be compared descriptively against each other and, moreover, the random slopes models will be tested against the random intercept models via likelihood ratio tests. Note that these likelihood ratio tests are known to be overly conservative.

Furthermore, in the case of the metric outcomes, we will derive additionally confidence intervals and p-values via non-parametric bootstrapping using 2000 replications. These sensitivity analyses are planned to get confidence intervals and p-values independent on the parametric assumptions of Gaussian distributions and homoscedasticity. Especially in the case of neuropsychiatric symptoms, baseline analyses indicated a skewed distribution for the NPI scores.

2.3.1.4 Secondary Analyses

In secondary analyses, the above described statistical modelling will be performed stratified for the living situation (living alone vs. not living alone). As the intervention targets also the care-giver and

the social context of a person suffering from dementia, it is very plausible that the effects of the intervention are different for persons living alone and persons living not alone. For example, the care-giver burden might response differentially to the intervention. Thus, effect sizes of the intervention for the efficacy endpoints will be derived once for persons living alone and once for persons not living alone. Additionally, to allow an inference-statistical assessment, a study-group living status interaction term will be introduced in the models explained above. A significant interaction term will be interpreted correspondingly that the intervention does not have the same effect for individuals living alone and individuals living not alone.

Moreover, several subgroup analyses regarding differential treatment effects are planned following the workflow of the described stratification living alone vs. not living alone. The subgroups under consideration will be:

1) Moderate/severe vs. Mild dementia
2) Men vs. Women
3) Age under 80 vs Age above 80

2.3.2 Intention to Treat (ITT) Analyses

To control a possible attrition bias, intention to treat analyses will be performed using multiple imputation techniques for the imputation of missing data. The method of imputation was not defined in the first version of the analyses plan. It was defined in March 2016 after the study statistician had completed a workshop for the handling of missing data in the end of February in Stockholm (Metrika Consulting – The Nordic STATA Contributor).

2.3.2.1 Definition of the Analyses Set

The ITT analyses will be carried out on the subsample of baseline-sample with baseline data regarding the primary efficacy endpoint under consideration. Thus, individuals which dropped out during the baseline assessment before the efficacy endpoint was surveyed will be excluded from the intention to treat analyses as there is not the necessary data available to allow the imputation of the follow data.

Missing values in the follow-up variable of the primary efficacy outcome will be imputed if the baseline value is not missing. Hence the ITT analyses can be seen as partial ITT analyses. The attrition bias caused by drop-out between the measurement points will be controlled by the imputation. A possible attrition bias caused by drop-out during baseline assessment will be not controlled by the ITT analyses proposed here.

2.3.2.2 Methods of Imputation

The imputation model will be chosen congenial to the analyses model with the difference that the GP will be included as a fixed effect in the imputation model. Thus, the model of imputation will be a linear regression for the care-giver burden, quality of live and NPI and logistic for antidementive drug use and the PIM, including the respective baseline variable, age, sex, the living status and the study group variable as predictors. The method of imputation is chosen to be multiple imputations via chained equations, using 50 runs of imputations. Standardized effect sizes will be also computed from these imputations and compared to the estimates of the per protocol analyses. Note that an important statistical pre-requisite for the usage of imputations is that the missing of the outcome variable is stochastically independent from the value of the outcome variable at follow-up given its baseline value.

2.3.2.3 Primary Efficacy Analyses (ITT)

The ITT analyses will be performed following the schedule of the per protocol analyses with the only difference being that missing follow-up values will be imputed.

2.3.2.4 Sensitivity Analyses (ITT)

The sensitivity analyses described in the per protocol analyses are not feasible using multiple imputation for computational reasons (bootstrap, random slopes). We plan, however, to use a second imputation model (predictive mean matching) for sensitivity analyses to test the dependency of the results on the concrete imputation method. Thus, we will rerun the ITT analyses only with the

difference that the imputation models (including the same variables as before) will be based on predictive mean matching. Again, 50 runs of imputations will be used.

2.3.2.5 Secondary Analyses (ITT)

The same analyses will be carried out as described in the per protocol analyses with the difference that missing values in the outcome variable at follow-up will be imputed.

2.4 Methods against Bias

Here, we will describe the statistical treatment of potential bias. The methods used in the design of DelpHi-trial to reduce a priori certain kind of bias will be not discussed. Three types of bias need special attention in the DelpHi trial:

1) Selection Bias
2) Attrition Bias
3) Performance Bias

2.4.1 Selection Bias

Although the trial is randomized it is possible that there is selection bias leading to differential baseline values in the primary efficacy outcomes regarding control and intervention group. As the baseline value of the outcome variable is included as covariate in the primary analysis model, the effect sizes of the intervention are thereby independent of baseline differences. Regardless, we will screen the two study group on differences regarding primary and secondary outcome variables, sociodemographic variables and study parameters on differences using generalized linear mixed models with the baseline variable as dependent variable and the study group as predictor. The GP will be included as random effect. The model specification will be specified according the type of variable tested (e.g. logistic for binary variables, Gaussian for metric variables, poisson for count outcomes).

2.4.2 Attrition Bias

We will perform ITT analyses as described to reduce the impact of drop-out during the follow-up interval. To check, whether systematic drop-out during the baseline assessment may influence the results, we will run a drop-out analyses. To this point, we will fit multivariable logistic regressions with drop-out (yes/no) being the dichotomous outcome and the study group, sociodemographic and the screening value of the DEMTECT as predictors. These analyses will be performed three-times:

1) Drop-out overall
2) Drop-out due to death
3) Drop-out due to withdrawal of informed consent

If it would be possible to predict drop-out during baseline by study-group parameters, it would be possible that attrition bias is still present despite the ITT analyses. In this case, the results of the study have to be treated cautiously.

2.4.3 Performance Bias

As the DelpHi-Trial is conducted in the setting of primary care, the control of the treatment in the control group as "care as usual" is not possible. It could be that already the inclusion of a GP into the study will change the treatment of the included patient. This effect could easily mask potential intervention effects. Thus, a non-significant result cannot be easily interpreted. The treatment adherence of the GPs in the intervention group was evaluated and generally quite high. Nevertheless, it is very plausible that the GPs were different in their adherence to the study protocol. For this reason, we performed special sensitivity analyses (see section "per protocol analyses"). These sensitivity analyses deliver a weak test of the robustness of the effects despite the heterogeneity between the GPs.

2.5 Post Hoc Power Calculations

The actual power of the trial was assessed during the baseline sampling when first estimates of the ICCs where known, for reasons explained above. Table 1 gives the estimates for the ICC of the baseline values of the primary efficacy endpoints and the detectable intervention effect (power 80%) given an assumed coefficient of variation of cluster sizes of 1, a baseline follow-up correlation of 0.6,

a mean cluster size of 5 and 100 clusters (GP) included into analysis (total n=500). The power analyses were done with STATA 13/SE using the package *clustersampsi*. Note that the estimates reported in table 1 were derived while the baseline sampling was still ongoing.

Table 1: Detectable Difference (power 80%) between Intervention (n=250) and Control group (250)

	Intra-Class Correlation	Detectable Difference	Model
Quality of Life	0.061	0.26	linear
Neuropsychiatric Symptoms	0.361	0.43	linear
Caregiver Burden	0.192	0.35	linear

3 References

1. Logsdon, R.G., et al., *Quality of life in Alzheimer's disease: Patient and caregiver reports.* Journal of Mental health and Aging, 1999. **5**(1): p. 21-32.
2. Zank, S., C. Schacke, and B. Leipold, *Berliner Inventar zur Angehörigenbelastung - Demenz (BIZA-D).* Zeitschrift für Klinische Psychologie und Psychotherapie, 2006. **35**(4): p. 296-305.
3. Cummings, J.L., *The Neuropsychiatric Inventory: assessing psychopathology in dementia patients.* Neurology, 1997. **48**(5 Suppl 6): p. S10-S16.
4. Wucherer, D., et al., *Antidementia drug treatment in people screened positive for dementia in primary care.* J Alzheimers Dis, 2015. **44**(3): p. 1015-1021.
5. WIdO (Wissenschaftliches Institut der AOK) *GKV-Arzneimittelindex*. http://www.wido.de/amtl_atc-code.html, 2016.
6. (DIMDI), D.I.f.m.D.u.I. *Anatomisch-therapeutisch-chemische Klassifikation mit Tagesdosen. Amtliche Fassung des ATC-Index mit DDD-Angaben für Deutschland im Jahre 2014.* 2016 2016 [cited 2016 16.09.2016].
7. Holt, S., S. Schmiedl, and P.A. Thurmann, *Potentially inappropriate medications in the elderly: the PRISCUS list.* Dtsch Arztebl Int, 2010. **107**(31-32): p. 543-551.
