# Supplementary material for: Clinical Outcomes and Cost-Effectiveness of Collaborative Dementia Care: A Secondary Analysis of a Cluster Randomized Clinical Trial
Source: JAMA Netw Open. 2024 Jul 5;7(7):e2419282. doi: 10.1001/jamanetworkopen.2024.19282 (PMC11227088; doi:10.1001/jamanetworkopen.2024.19282)
Supplement: Supplement 2. — eTable 1. Drop-Out Analysis for Patients Excluded From Outcome and Cost-Effectiveness Analysis eTable 2. Drop-Out Analysis for Patients Who Dropped Out After Completing the Baseline Assessment Before Follow-Up at Years 1, 2, and 3 eTable 3. Cost Calculation for the Implementation of 1 Nurse Carrying Out the Collaborative Dementia Care Management for 90 Patients per Year eTable 4. Cost Categories and Unit Costs for Monetary Valuation of Medical and Formal Health Care Resources and Services eTable 5. Regression Analyses for the Treatment Effect of CDCM vs Usual Care eTable 6. Regression Analyses for the Treatment Effect of CDCM vs Usual Care Separately Over Years for Complete Cases eTable 7. Regression Analyses for the Treatment Effect of CDCM vs Usual Care Separately Over 1, 2, and 3 Years eTable 8. Adjusted Incremental Cost and Effects of CDCM vs Usual Care for Year 1, 2, 3, and Cumulative eTable 9. Unadjusted Health Care Resource Utilization, Cost, and Quality-Adjusted Life of CDCM vs Usual Care for Year 1, 2, 3, and Cumulative eTable 10. Adjusted Incremental Cost and Effects on QALYs of the Collaborative Model of Dementia Care vs Usual Care for Year 1, 2, 3, and Cumulative, Separately for Those Living Alone and Not Living Alone eTable 11. Comparison of the Participant Characteristics at Baseline and 3 Years After Baseline eFigure 1. Flow Chart of the DelpHi-MV Trial eFigure 2. Cost-Effectiveness Acceptability Curves of the Scenario Analyses eFigure 3. Cost-Effectiveness Plane and Cost-Effectiveness Acceptability Curves of the CDCM vs Usual Care After 3 Years, Shown Separately for Patients Living Alone and Not Living Alone eAppendix 1. Methods Used to Handle Missing Data and That Used Stata Code eAppendix 2. Intraclass Correlation and Design Effects for Each Outcome and Time Point eAppendix 3. Standardized Measure of Effect Size for Each Outcome and Time Point eAppendix 4. Calculation of Utility Values and Quality-Adjusted Life-Years [file jamanetwopen-e2419282-s002.pdf]

Supplementary Online Content

Michalowsky B, Blotenberg I, Platen M, et al. Clinical outcomes and cost-effectiveness of collaborative dementia care: a secondary analysis of a cluster randomized clinical trial. *JAMA Netw Open*. 2024;7(7):e2419282. doi:10.1001/jamanetworkopen.2024.19282

eTable 1. Drop-Out Analysis for Patients Excluded From Outcome and Cost-Effectiveness Analysis
eTable 2. Drop-Out Analysis for Patients Who Dropped Out After Completing the Baseline Assessment Before Follow-Up at Years 1, 2, and 3
eTable 3. Cost Calculation for the Implementation of 1 Nurse Carrying Out the Collaborative Dementia Care Management for 90 Patients per Year
eTable 4. Cost Categories and Unit Costs for Monetary Valuation of Medical and Formal Health Care Resources and Services
eTable 5. Regression Analyses for the Treatment Effect of CDCM vs Usual Care
eTable 6. Regression Analyses for the Treatment Effect of CDCM vs Usual Care Separately Over Years for Complete Cases
eTable 7. Regression Analyses for the Treatment Effect of CDCM vs Usual Care Separately Over 1, 2, and 3 Years
eTable 8. Adjusted Incremental Cost and Effects of CDCM vs Usual Care for Year 1, 2, 3, and Cumulative
eTable 9. Unadjusted Health Care Resource Utilization, Cost, and Quality-Adjusted Life of CDCM vs Usual Care for Year 1, 2, 3, and Cumulative
eTable 10. Adjusted Incremental Cost and Effects on QALYs of the Collaborative Model of Dementia Care vs Usual Care for Year 1, 2, 3, and Cumulative, Separately for Those Living Alone and Not Living Alone
eTable 11. Comparison of the Participant Characteristics at Baseline and 3 Years After Baseline
eFigure 1. Flow Chart of the DelpHi-MV Trial
eFigure 2. Cost-Effectiveness Acceptability Curves of the Scenario Analyses
eFigure 3. Cost-Effectiveness Plane and Cost-Effectiveness Acceptability Curves of the CDCM vs Usual Care After 3 Years, Shown Separately for Patients Living Alone and Not Living Alone
eAppendix 1. Methods Used to Handle Missing Data and That Used Stata Code
eAppendix 2. Intraclass Correlation and Design Effects for Each Outcome and Time Point
eAppendix 3. Standardized Measure of Effect Size for Each Outcome and Time Point
eAppendix 4. Calculation of Utility Values and Quality-Adjusted Life-Years

This supplementary material has been provided by the authors to give readers additional information about their work.

© 2024 Michalowsky B et al. *JAMA Network Open*.

eTable 1. Drop-out-analysis for patients excluded from outcome and cost-effectiveness analysis

	**Excluded from outcome analysis**		**Excluded from cost-effectiveness analysis**	
	OR (95%-CI)	*p-value*	OR (CI)	*p-value*
Study group (Ref. intervention)	**0.61 (0.42 – 0.89)**	**0.012**	**0.46 (0.25 – 0.85)**	**0.014**
Age	1.04 (1.00 – 1.08)	0.053	1.00 (0.95 – 1.06)	0.966
Sex (Ref. female)	**0.51 (0.31 – 0.84)**	**0.008**	0.87 (0.49 – 1.54)	0.624
Informal caregiver (Ref. available)	**0.47 (0.28 – 0.79)**	**0.004**	**0.35 (0.19 – 0.66)**	**0.001**
Living situation (Ref. alone)	1.09 (0.67 – 1.79)	0.727	0.94 (0.57 – 1.56)	0.806
Comorbidity (ICD-10 Diagnoses)	**0.96 (0.93 – 0.99)**	**0.016**	0.96 (0.90 – 1.02)	0.163
Cognitive impairment (MMSE)	**0.94 (0.91 – 0.98)**	**0.001**	**0.94 (0.91 – 0.98)**	**0.001**
Depression (GDS)	1.08 (0.98 – 1.18)	0.123	0.98 (0.86 – 1.11)	0.751
Activities in daily living (B-ADL)	**1.13 (0.01 – 5.09)**	**0.016**	0.96 (0.10 – 190.05)	0.469

Abbreviations: OR, odds ratios; Ref, reference; CI, confidence interval; MMSE, Mini-Mental State Examination; B-ADL, Bayer-Activities of Daily Living Scale; GDS, Geriatric Depression Scale; ICD, International Statistical Classification of Diseases and Related Health Problems, SD, standard deviation.

Footnotes: Drop out due to death included in cost-utility-analysis as zero cost and utility values; Multivariate logistic regression analysis with random effects for the general practitioner.

© 2024 Michalowsky B et al. *JAMA Network Open*.

eTable 2. Drop-out-analysis for patients who dropped out after completing the baseline assessment before follow-up 1, 2 and 3

	Withdrawal of IC		Death		Overall	
	OR (95%-CI)	*p-value*	OR (95%-CI)	*p-value*	OR (95%-CI)	*p-value*
**Lost to follow – up 1**						
Study group (Ref. control group)	**0.29 (0.11 – 0.77)**	**0.014**	0.76 (0.30 – 1.90)	0.553	**0.32 (0.14 – 0.71)**	**0.006**
Age	0.97 (0.89 – 1.06)	0.456	1.00 (0.93 – 1.08)	0.919	1.0 (0.94 – 1.06)	0.904
Sex (Ref. female)	0.89 (0.38 – 2.12)	0.792	**0.28 (0.11 – 0.75)**	**0.012**	**0.48 (0.25 – 0.95)**	**0.034**
Informal caregiver (Ref. available)	**0.30 (0.11 – 0.82)**	**0.019**	1.03 (0.36 – 2.96)	0.955	**0.45 (0.22 – 0.93)**	**0.031**
Living situation (Ref. alone)	0.77 (0.31 – 1.88)	0.560	2.66 (0.95 – 7.43)	0.063	1.07 (0.55 – 2.07)	0.835
Comorbidity (ICD-10 Diagnoses)	0.98 (0.90 – 1.06)	0.588	**0.93 (0.88 – 0.98)**	**0.005**	**0.95 (0.90 – 1.00)**	**0.047**
Cognitive impairment (MMSE)	0.96 (0.89 – 1.04)	0.282	0.97 (0.87 – 1.07)	0.541	0.95 (0.90 – 1.01)	0.105
Depression (GDS)	0.96 (0.79 – 1.17)	0.675	1.19 (1.00 – 1.42)	0.056	1.07 (0.95 – 1.19)	0.272
Activities in daily living (B-ADL)	0.92 (0.71 – 1.20)	0.532	1.09 (0.85 – 1.39)	0.495	1.01 (0.87 – 1.18)	0.850
**Lost to follow – up 2**						
Study group (Ref. intervention)	1.20 (0.06 – 22.40)	0.900	0.65 (0.27 – 1.60)	0.345	0.83 (0.35 – 1.98)	0.664
Age	0.93 (0.76 – 1.14)	0.488	1.06 (0.98 – 1.15)	0.142	1.04 (0.95 – 1.14)	0.344
Sex (Ref. female)	1.06 (0.25 – 4.41)	0.939	0.52 (0.19 – 1.39)	0.188	0.51 (0.22 – 1.17)	0.113
Informal caregiver (Ref. available)	1.02 (0.10 – 10.27)	0.987	0.52 (0.20 – 1.31)	0.163	0.61 (0.27 – 1.37)	0.229
Living situation (Ref. alone)	0.89 (0.11 – 7.45)	0.914	0.77 (0.28 – 2.07)	0.598	0.76 (0.30 – 1.95)	0.566
Comorbidity (ICD-10 Diagnoses)	0.88 (0.74 – 1.06)	0.169	0.97 (0.89 – 1.05)	0.384	0.94 (0.89 – 1.01)	0.078
Cognitive impairment (MMSE)	1.17 (0.97 – 1.41)	0.099	0.97 (0.89 – 1.07)	0.548	0.99 (0.90 – 1.08)	0.771
Depression (GDS)	1.29 (0.82 – 2.03)	0.272	1.10 (0.93 – 1.31)	0.240	1.12 (0.96 – 1.30)	0.141
Activities in daily living (B-ADL)	1.10 (0.62 – 1.95)	0.751	**1.26 (1.06 – 1.49)**	**0.009**	1.21 (0.99 – 1.47)	0.060
**Lost to follow – up 3**						
Study group (Ref. intervention)	1.05 (0.21 – 5.27)	0.951	0.75 (0.31 – 1.80)	0.511	1.01 (0.57 – 1.81)	0.963
Age	0.98 (0.85 – 1.13)	0.810	1.06 (0.98 – 1.14)	0.160	1.03 (0.97 – 1.08)	0.379
Sex (Ref. female)	1.04 (0.26 – 4.13)	0.951	0.46 (0.20 – 1.06)	0.068	0.71 (0.40 – 1.28)	0.248
Informal caregiver (Ref. available)	1.17 (0.27 – 5.13)	0.835	1.21 (0.44 – 3.34)	0.707	0.93 (0.50 – 1.74)	0.827
Living situation (Ref. alone)	0.73 (0.17 – 3.06)	0.663	1.30 (0.56 – 2.99)	0.540	1.17 (0.62 – 2.20)	0.632
Comorbidity (ICD-10 Diagnoses)	1.00 (0.94 – 1.07)	0.973	1.00 (0.93 – 1.06)	0.893	1.01 (0.97 – 1.05)	0.610
Cognitive impairment (MMSE)	1.05 (0.88 – 1.26)	0.569	0.94 (0.85 – 1.04)	0.203	0.98 (0.91 – 1.05)	0.540
Depression (GDS)	0.97 (0.69 – 1.35)	0.834	1.03 (0.85 – 1.23)	0.778	0.96 (0.82 – 1.13)	0.639
Activities in daily living (B-ADL)	0.97 (0.67 – 1.42)	0.887	1.15 (0.96 – 1.37)	0.129	**1.16 (1.01 – 1.32)**	**0.036**

Abbreviations: OR, Odds Ratios; Ref, reference; CI, confidence interval; MMSE, Mini-Mental State Examination; B-ADL, Bayer-Activities of Daily Living Scale; GDS, Geriatric Depression Scale; ICD, International Statistical Classification of Diseases and Related Health Problems, SD, standard deviation; IC, informed consent.

Footnotes: Drop out due to death included in cost-utility-analysis as zero cost and utility values; Multivariate logistic regression analysis with random effects for the general practitioner.

© 2024 Michalowsky B et al. *JAMA Network Open*.

eTable 3. Cost calculation for the implementation of one nurse carrying out the collaborative dementia care management for 90 patients per year

Type of cost	Annual costs
**Personnel expenses**	**35.939 €**
Gross salary	29.268 €
Incidental wage cost	6.671 €
**Material costs**	**4.507 €**
Rental cost	1.051 €
Service	301 €
Material cost for home visits	148 €
Office supplies	259 €
Cellphone fee	512 €
Operating costs for the company car	1.975 €
Cost for IT-Support	258 €
**Amortizations**	**6.401 €**
Company car (life=6 years)	2.582 €
Office furniture (life=13 years)	167 €
Workplace/computer (life=3 years)	445 €
Printer (life=3 years)	82 €
Cell phone (life=8 years)	55 €
Tablet-computer (life=3 years)	1.406 €
Software license (life=5 years)	1.000 €
Cost for dementia-specific qualification (life=10 years)	661 €
**Total cost per Dementia Care Manager**	**46.847 €**
**Total cost per patient**[1]	**520 €**

[1] based on the assumption that one dementia care manager could treat 90 patients annually.

© 2024 Michalowsky B et al. *JAMA Network Open*.

eTable 4. Cost categories and unit costs for monetary valuation of medical and formal healthcare resources and services

Cost categories	Services	Units	Unit costs[†]	Unit cost & source for monetary valuation
**Medical care**				
**Out-patient physician treatment**	GP or specialists	Contact	20.95€ - 81.56€, depending on specialization	Cost per contact [48]
**In-patient treatment**	In-hospital treatment and rehabilitation	Days	593.04€ and 121.85, respectively	Average per diem cost for in-hospital treatment in Mecklenburg-Western Pomerania & for specialization of rehabilitation [48]
**Medications**	Regularly prescribed drugs (Rx-drugs)	Quantity	Market prices, 253.58€[‡]	Pharmaceutical Index of the Scientific Institute of the AOK [67]
**Medical aids**	Aids such as tub-lifts, tub-seats, walking sticks, walkers and others	Quantity	Market prices, 168.92€[‡]	Market prices
**Other out-patient treatment**	Occupational therapy, speech therapy, physiotherapy and others	Contacts	20.46€	Cost per contact & reimbursement schedules of statutory health insurance [68]
**Formal care**				
**Ambulatory care**	Home care provided by professionals	Quantity/ Contacts	Market prices, 11.48€[‡]	Market prices for Mecklenburg Western-Pomerania
**Day care**	Partial in-patient day- and night-time nursing care and short-term care	Days	43.31€, 50.74€ and 57.94€, depending on care level[*]	Insurance rates of compulsory long-term in relation to patient level of care, including cost for board and lodging [69]
**Nursing home care**	Long-term care (institutionalization)	Days	61.17€, 76.36€ and 92.39€, depending on care level[*]	Insurance rates of compulsory long-term in relation to patient level of care, including cost for board and lodging [69]

GP, general practitioner; AOK, allgemeine Ortskrankenkasse; [*]care level one: mild functional impairment, care level two: moderate functional impairment, care level three: severe functional impairment; [‡] when drugs, aids or services were unknown or market prices were not available; [†] inflation included.

© 2024 Michalowsky B et al. *JAMA Network Open*.

eTable 5. Regression analyses for the treatment effect of the collaborative model of dementia care compared with usual care

	**Standardized treatment effect of the cDCM**			**Explained variance**
	Adjusted mean difference	*95%-CI*	*p-value*	R^2
**Behavioral and psychological symptoms of dementia (NPI)[1]**				
year 1	**-0.57**	**-0.84 – -0.29**	***<0.001***	***0.275******
year 2	**-0.58**	**-0.91 – -0.24**	***0.001***	***0.244******
year 3	**-0.73**	**-1.20 – -0.25**	***0.003***	***0.194******
**Caregiver burden (BIZA-D)**				
year 1	-0.18	-0.39 – 0.02	*0.081*	*0.511****
year 2	-0.07	-0.31 – 0.18	*0.602*	*0.316****
year 3	**-0.59**	**-0.81 – -0.37**	***<0.001***	***0.353******
**Quality of Life (QoL-AD)**				
year 1	0.03	-0.04 – 0.09	*0.394*	*0.430****
year 2	0.06	-0.01 – 0.01	*0.130*	*0.325****
year 3	0.06	-0.02 – 0.13	*0.139*	*0.283****
**Mental Health (SF-12 MCS)**				
year 1	0.21	-0.01 – 0.45	*0.085*	*0.192****
year 2	**0.30**	**0.04 – 0.55**	***0.024***	***0.145******
year 3	**0.31**	**0.04 – 0.58**	***0.023***	***0.085******
**Physical HRQoL (SF-12 PCS)**				
year 1	0.01	-0.20 – 0.21	*0.914*	*0.417****
year 2	0.07	-0.15 – 0.29	*0.540*	*0.326****
year 3	0.14	-0.09 – 0.37	*0.232*	*0.312****
**Health Utility (SF-6D)**				
year 1	0.06	-0.05 – 0.17	0.271	*0.414****
year 2	0.07	-0.04 – 0.17	0.203	*0.274****
year 3	0.04	-0.04 – 0.13	0.328	*0.159****
	OR	*95%-CI*	*p-value*	R^2
**Antidementia drug treatment[1]**				
year 1	**2.56**	**1.18 – 5.55**	***0.017***	***0.344******
year 2	**3.06**	**1.39 – 6.75**	***0.006***	***0.342******
year 3	**1.91**	**0.96 – 3.77**	***0.065***	***0.275******
**Potentially inappr. medication[2]**				
year 1	1.94	0.91 – 4.18	*0.088*	*0.209****
year 2	1.37	0.66 – 2.87	*0.397*	*0.168****
year 3	1.76	0.82 – 3.94	*0.142*	*0.139****

Abbreviations: BIZA-D, Berlin Inventory of Caregivers' Burden with Dementia Patients-questionnaire; NPI, Neuropsychiatric Inventory; ; SF-12, 12-Item Short-Form Health Survey; SF-12 PCS, Physical Component Score; SF-12 MCS, Mental Component Score; B-ADL, Bayer–Activities of Daily Living Scale; GDS, Geriatric Depression Scale; MMSE, Mini-Mental State Examination; QoL-AD; Quality of Life in Alzheimer's Diseases; cDCM, collaborative dementia care management; inappr., inappropriate; b, beta coefficient; CI, confidence interval.

[1]Antidementia drugs: donepezil, rivastigmine, galantamine, memantine and donepezil and memantine; [2]According to PRISCUS list

Footnotes: Linear regression analyses adjusted for age, sex, living situation and baseline value; the study group was the predictor of interest; p-values are given two-sided.

© 2024 Michalowsky B et al. *JAMA Network Open*.

eTable 6. Regression analyses for the treatment effect of the collaborative model of dementia care compared with care as usual separately over years for complete cases

	**Standardized treatment effect of the cDCM**			**Explained variance**
	Adjusted mean difference	95%-CI	*p-value*	R^2
**Behavioral and psychological symptoms of dementia (NPI)[1]**				
year 1	**-0.70**	**-1.00 – -0.43**	***<0.001***	*0.325****
year 2	**-0.52**	**-0.81 – -0.22**	***<0.001***	*0.249****
year 3	**-0.82**	**-1.22 – -0.42**	***<0.001***	*0.230****
**Caregiver burden (BIZA-D)**				
year 1	-0.18	-0.40 – 0.03	*0.218*	*0.520****
year 2	-0.10	-0.37 – 0.17	*0.756*	*0.276****
year 3	**-0.55**	**-0.80 – -0.31**	***<0.001***	*0.339****
**Quality of Life (QoL-AD)**				
year 1	0.08	-0.12 – 0.29	*0.425*	*0.417****
year 2	**0.29**	**0.07 – 0.51**	***0.009***	*0.376****
year 3	0.19	-0.04 – 0.42	*0.119*	*0.285****
**Mental Health (SF-12 MCS)**				
year 1	0.17	-0.08 – 0.42	*0.176*	*0.173****
year 2	**0.35**	**0.10 – 0.61**	***0.006***	*0.156****
year 3	**0.28**	**0.02 – 0.55**	***0.033***	*0.091****
**Physical HRQoL (SF-12 PCS)**				
year 1	0.03	-0.16 – 0.24	*0.716*	*0.447****
year 2	0.10	-0.11 – 0.32	*0.363*	*0.353****
year 3	0.15	-0.07 – 0.38	*0.118*	*0.323****
**Health Utility (SF-6D)**				
year 1	0.13	-0.08 – 0.34	*0.229*	*0.424****
year 2	0.22	-0.01 – 0.45	*0.055*	*0.342****
year 3	0.18	-0.07 – 0.43	*0.159*	*0.182****
	OR	*95%-CI*	*p-value*	R^2
**Antidementia drug treatment[1]**				
year 1	2.10	0.91 – 4.83	*0.081*	*0.360****
year 2	**2.82**	**1.21 – 6.55**	***0.016***	***0.347*****
year 3	1.65	0.79 – 3.40	*0.179*	*0.275****
**Potentially inappr. medication[2]**				
year 1	1.95	0.91 – 4.18	*0.088*	*0.209****
year 2	1.37	0.66 – 2.87	*0.397*	*0.168****
year 3	1.80	0.82 – 3.94	*0.142*	*0.139****

Abbreviations: BIZA-D, Berlin Inventory of Caregivers' Burden with Dementia Patients-questionnaire; NPI, Neuropsychiatric Inventory; SF-12, Short-Form-Health Survey; cDCM, collaborative dementia care management;, SF-12 PCS, Physical Component Score; SF-12 MCS, Mental Component Score; SD, standard deviation; CI, confidence intervalm; b, beta

Footnotes: NPI and BIZA-D only consider cases where a caregiver was present.

© 2024 Michalowsky B et al. *JAMA Network Open*.

eTable 7. Regression analyses for the treatment effect (secondary outcomes) of the collaborative model of dementia care compared with care as usual separately over one, two and three years

	Treatment effect of the cDCM			Explained variance
	Adjusted mean difference	95%-CI	*p-value*	R^2
**Cognitive status (MMSE)**				
year 1	-0.63	-1.99 – 0.73	*0.365*	*0.521****
year 2	**-2.08**	**-3.88 – -0.28**	***0.024***	***0.386*****
year 3	-0.50	-2.77 – 1.77	*0.666*	*0.246****
**Functional impairment (B-ADL)**				
year 1	0.09	-0.42 – 0.59	*0.736*	*0.484****
year 2	0.35	-0.27– 0.97	*0.265*	*0.393****
year 3	0.44	-0.21– 1.08	*0.184*	*0.336****
**Geriatric Depression Score (GDS)**				
year 1	-0.17	-0.66 – 0.32	*0.495*	*0.415****
year 2	0.20	-0.32 – 0.71	*0.458*	*0.253****
year 3	**-0.62**	**-1.14 – -0.10**	***0.020***	***0.251*****

Abbreviations: B-ADL, Bayer–Activities of Daily Living Scale; GDS, Geriatric Depression Scale; MMSE, Mini-Mental State Examination; QoL-AD; Quality of Life in Alzheimer's Diseases; cDCM, collaborative dementia care management; b, beta coefficient.

[1]Antidementia drugs: donepezil, rivastigmine, galantamine, memantine and donepezil and memantine

[2]According to PRISCUS list

Footnotes: Linear regression analyses adjusted for age, sex, living situation and baseline value; the study group was the predictor of interest; p-values are given two-sided.

© 2024 Michalowsky B et al. *JAMA Network Open*.

eTable 8. Adjusted incremental cost and effects of the collaborative model of dementia care versus usual care for year one, two, three and cumulated (including death patients, incurring no costs)

	Year 1	*Year 2*	*Year 3*	*Total (cumulated)*
	Adjusted difference[1](SE)	*Adjusted difference[1] (SE)*	*Adjusted difference[1](SE)*	*Adjusted difference[1] (SE)*
**Healthcare cost**	**-1,061 (1,218)**	**-238 (1,509)**	**+1,217 (1,193)**	**-82 (2,989)**
**Medical treatments**	**-255 (946)**	**-92 (1,319)**	**-289 (578)**	**-636 (2,089)**
Physicians	-8 (25)	-22 (25)	-57 (37)	-87 (67)
In-hospital	-576 (878)	-730 (1,261)	-545 (415)	-1,853 (1,903)
Medications	+306 (152)*	+531 (208)**	+81 (216)	+919 (421)*
Medical aids	+42 (127)	+194 (113)	+203 (80)**	+439 (244)
Therapies	-19 (38)	-64 (50)	+28 (52)	-54 (108)
**Formal care**	**-806 (601)**	**-146 (702)**	**+1,507 (863)**	**+554 (1,766)**
Day/ night care	-345 (249)	-101 (184)	+289 (271)	-157 (510)
Ambulatory care	-354 (417)	-305 (425)	+295 (408)	-364 (918)
Nursing home	-106 (340)	+206 (616)	+921 (754)	-1,076 (1,518)
**QALYs**	**+0.03 (0.02)**	**+0.06 (0.03)***	**0.06 (0.03)***	**+0.149 (0.07)***
			**Cost for intervention (cDCM),** mean (SE)	**+520 (0)**
			**Incremental costs (cDCM vs usual care),** mean (SE) [95%CI]	**+437 (2,989) [-5,438 – 6,313]**
			**Incremental QALY (cDCM vs usual care),** mean (SE) [95%CI]	**+0.137 (0.07)* [0.01 – 0.27]**
			**Incremental cost per QALY gained**	**3,186€/ QALY**

Abbreviations: QALYs, quality-adjusted life years; SE, standard error; b, beta coefficient.

[1]positive (negative) beta coefficients indicate cost increases (cost savings) of the collaborative dementia care management, meaning that receiving the model of collaborative care increases (decreases) costs; *p<0.05; **p<0.01; ***p<0.001;

© 2024 Michalowsky B et al. *JAMA Network Open*.

eTable 9. Unadjusted healthcare resource utilization, cost and quality-adjusted life of the collaborative model of dementia care versus usual care for year one, two, three and cumulated (including death patients, incurring no costs)

	Year 1		*Year 2*		*Year 3*		*Total (cumulated)*	
	cDCM N=303	***Care as Usual N=125***	***cDCM N=303***	***Care as Usual N=125***	***cDCM N=303***	***Care as Usual N=125***	***cDCM N=303***	***Care as Usual N=125***
Healthcare resource use, mean (SD)								
**Medical treatments**								
Physician, visits	11.3 (6.3)	12.0 (6.9)	11.1 (6.4)	11.6 (8.4)	8.5 (8.2)	10.2 (13.4)	30.9 (15.7)	33.8 (22.6)
In-hospital, days	3.9 (12.8)	4.8 (13.8)	5.4 (13.2)	6.6 (29.2)	2.6 (6.2)	3.5 (6.8)	11.9 (20.8)	14.9 (42.5)
Medications, number	7.0 (3.3)	6.5 (3.1)	**7.1 (3.0)** [b]	**6.2 (2.8)** [b]	5.8 (4.5)	5.3 (4.4)	**19.9 (8.7)** [a]	**17.9 (8.6)**[a]
Medical aids, number	6.0 (3.0)	5.6 (3.0)	**7.2 (3.1)** [b]	**6.2 (2.8)** [b]	**6.4 (5.0)** [a]	**5.1 (4.4)** [a]	**19.6 (8.3)** [b]	**17.0 (7.5)**[b]
Therapies, visits	6.0 (15.9)	6.3 (23.0)	8.0 (21.4)	10.7 (32.9)	8.2 (26.2)	6.6 (26.0)	22.2 (52.5)	23.6 (59.9)
**Formal care**								
Day/ night care, days	18.6 (45.3)	22.5 (70.5)	17.4 (38.5)	16.3 (43.7)	17.9 (58.8)	10.8 (51.7)	54.2 (105.9)	49.6 (120.0)
Ambulatory care, visits	181.8 (360.2)	181.0 (404.6)	205.0 (363.4)	202.2 (421.0)	165.8 (375.6)	128.0 (343.0)	552.6 (798.8)	511.2 (943.1)
Nursing home, days	9.2 (47.4)	9.3 (45.6)	28.6 (89.5)	20.7 (81.7)	46.6 (116.1)	27.5 (87.1)	84.4 (222.6)	57.5 (199.8)
Time to institutionalization*, days	188.9 (128.3)	171.0 (92.3)	535.6 (88.9)	462.5 (135.1)	867.9 (121.8)	930.9 (70.7)	543.5 (303.7)	564.5 (376.1)
Costs in Euros, mean (SD)								
**Healthcare cost**	**9,503 (11,622)**	**9,893 (12,366)**	**12,008 (11,838)**	**11,501 (19,638)**	**9,844 (11,434)**	**8,403 (11,168)**	**31,355 (26,528)**	**29,797 (34,763)**
**Medical treatments**	**5,940 (8,700)**	**6,083 (9,127)**	**7,080 (9,112)**	**7,029 (17,949)**	**4,257 (5,293)**	**4,574 (5,684)**	**17,277 (15,443)**	**17,686 (26,978)**
Physicians	367 (254)	384 (228)	349 (232)	379 (256)	**257 (271)**[a]	**331 (502)** [a]	972 (586)	1,094 (763)
In-hospital	2,489 (8,005)	3,019 (8,645)	3,341 (8,133)	4,036 (17,842)	1,570 (3,715)	2,121 (4,273)	7,400 (12,911)	9,186 (26,086)
Medications	**1,804 (1,521)** [a]	**1,496 (1,211)** [a]	**1,905 (2,164)**[b]	**1,348 (1,289)**[b]	1,264 (2,008)	1,191 (2,121)	**4,972 (4,153)**[a]	**4,036 (3,345)**[a]
Medical aids	1,158 (1,302)	1,045 (1,046)	**1,328 (1,200)**[a]	**1,055 (893)**[a]	**1,009 (780)**[a]	**804 (692)**[a]	**3,495 (2,510)**[a]	**2,903 (2,076)**[a]
Therapies	123 (325)	128 (471)	158 (423)	212 (650)	157 (500)	126 (495)	438 (1,031)	466 (1,186)
**Formal care**	**3,562 (5,742)**	**3,810 (7,509)**	**4,928 (6,830)**	**4,472 (7,609)**	**5,587 (8,543)** [a]	**3,829 (7,777)** [a]	**14,078 (17,442)**	**12,111 (19,312)**
Day/ night care	861 (2,140)	1,060 (3,253)	777 (1,746)	750 (2,038)	781 (2,613)	480 (2,393)	2,419 (4,800)	2,290 (5,556)
Ambulatory care	2,088 (3,621)	2,078 (4,646)	2,272 (4,027)	2,241 (4,665)	1,761 (4,016)	1,368 (3,668)	6,121 (8,859)	5,688 (10,510)
Nursing home	613 (3149)	673 (3,290)	1,880 (5,835)	1,480 (5,781)	3,045 (7,497)	1,981 (6,148)	5,537 (14,461)	4,134 (14,091)
**Cost for intervention (cDCM)**	520 (0)	0 (0)	0 (0)	0 (0)	0 (0)	560 (0)	520 (0)	0 (0)
**Total costs (incl. intervention costs)**	**10,023 (11,882)**	**9,893 (12,626)**	**12,008 (11,838)**	**11,501 (19,638)**	**9,844 (11,434)**	**8,403 (11,168)**	**31,875 (26,575)**	**29,797 (34,763)**
**QALYs**	**0.719 (0.19)**	**0.704 (0.21)**	**0.632 (0.26)**	**0.594 (0.28)**	**0.529 (0.31)**	**0.496 (0.31)**	**1.880 (0.69)**	**1.794 (0.72)**
**Incremental costs (cDCM vs usual care),** mean (SE) [95%CI]							**+2,078 €** (3,103) [-4,022 – 8,179]	
**Incremental QALY (cDCM vs usual care),** mean (SE) [95%CI]							**+0.085 QALY** (0.07) [-0.06 – 0.23]	
**Incremental cost per QALY gained**							**24,323€/ QALY**	

*from baseline [a] $p<0.05$; [b] $p<0.01$; [c] $p<0.001$

© 2024 Michalowsky B et al. *JAMA Network Open*.

eTable 10. Adjusted incremental cost and effects on QALYs of the collaborative model of dementia care versus usual care for year one, two, three and cumulated (including death patients, incurring no costs) separately for those living alone and those living not alone

	Year 1		***Year 2***		***Year 3***		***Total (cumulated)***	
	Alone (n=216)	*Not Alone (n=212)*	*Alone (n=216)*	*Not Alone (n=212)*	*Alone (n=216)*	*Not Alone (n=212)*	*Alone (n=216)*	*Not Alone (n=212)*
	Adj. diff.[1] (SE)	*Adj. diff.[1] (SE)*	*Adj. diff.[1] (SE)*	*Adj. diff.1 (SE)*	*Adj. diff.1 (SE)*	*Adj. diff.1 (SE)*	*Adj. diff.1 (SE)*	*Adj. diff.1 (SE)*
**Healthcare cost**	**-3,303 (1,602)***	**+714 (1,826)**	**-2,068 (2,682)**	**+1,481 (1,467)**	**+1,555 (1,816)**	**+1,088 (1,551)**	**-3,815 (4,706)**	**+3,283 (3,711)**
**Medical treatments**	**-1,510 (1,262)**	**+645 (1,408)**	**-1,176 (2,484)**	**+823 (1,024)**	**-219 (825)**	**-360 (823)**	**-2,907 (3,648)**	**+1,107 (2,133)**
Physicians	-14 (34)	0 (38)	-65 (33)*	+15 (37)	-31 (43)	-77 (60)	-111 (86)	-63 (102)
In-hospital	-1,872 (1,178)	+392 (1,307)	-2,115 (2,397)	+428 (925)	-1,045 (657)	-151 (518)	-5,034 (3,414)	+670 (1,780)
Medications	+312 (232)	+283 (193)	+758 (349)*	342 (239)	446 (222)*	-237 (370)	+1,516 (596)*	+388 (598)
Medical aids	+172 (185)	-84 (178)	+288 (163)*	+110 (158)	+336 (114)*	+105 (112)	+797 (353)*	+131 (341)
Therapies	-108 (57)	+54 (50)	-42 (47)	-73 (87)	-74 (50)	0 (92)	-76 (119)	-19 (181)
**Formal care**	**-1,791 (921)***	**+68 (774)**	**-890 (1,051)**	**657 (943)**	**+1,774 (1,368)**	**1,449 (1,054)**	**-908 (2,650)**	**+2,175 (2,349)**
Day/ night care	-588 (368)	-117 (339)	-441 (300)	+184 (219)	+283 (497)	+347 (232)	-747 (809)	+414 (629)
Ambulatory care	-524 (724)	-330 (434)	-354 (673)	-330 (533)	+436 (741)	+37 (366)	-442 (1,572)	-624 (990)
Nursing home	-678 (422)	+517 (529)	-95 (886)	+804 (848)	+1,055 (1,118)	+1,064 (1,014)	-281 (2,095)	+2,386 (2,178)
**Quality-adj. Life-Years (QALYs)**	**+0.028 (0.03)**	**+0.025 (0.03)**	**+0.093 (0.04)***	**0.033 (0.03)**	**0.103 (0.04)***	**0.022 (0.04)**	**+0.224 (0.10)***	**+0.079 (0.10)**
**Incremental Cost-Effectiveness Ratio (ICER)[2,3]**	**cDCM dominates**	**32,320€/QALY**	**cDCM dominates**	**46,810€/QALY**	**cDCM dominates**	**47,538€/QALY**	**cDCM dominates**	**47,538€/QALY**

[1]positive (negative) beta coefficients indicate cost increases (cost savings) of the collaborative dementia care management, meaning that receiving the model of collaborative care increases (decreases) costs; [2] including intervention costs of 520€; [3] cumulatively includes costs and QALYs from previous years in year 2 and 3; *p<0.05; **p<0.01; ***p<0.001; Abbreviations: cDCM, collaborative dementia care management; QALYs, Quality-adjusted Life Years; SE, standard error; b, beta coefficient.

© 2024 Michalowsky B et al. *JAMA Network Open*.

eTable 11. Comparison of the participant characteristics (efficacy analysis) at baseline and 3 years after baseline

	Baseline					Follow-up 3 (three years after baseline)				
	cDCM (n=221)		Usual Care (n=87)		*p-value*[a]	cDCM (n=221)		Usual Care (n=87)		*p-value*[a]
Demographics										
Age, mean (SD)	80.1	(5.3)	79.2	(4.5)	*0.153*	83.3	(5.2)	82.3	(4.5)	*0.138*
Sex (female), n (%)	142	(64.3)	50	(57.5)	*0.297*	142	(64.3)	50	(57.5)	*0.297*
Caregiver included, n (%)	159	(71.9)	54	(62.0)	*0.106*	123	(55.7)	45	(51.7)	*0.611*
Living Alone, n (%)	115	(52.0)	39	(44.8)	*0.311*	107	(48.4)	44	(50.6)	*0.800*
Clinical characteristics										
Cognitive status (MMSE), mean (SD)	22.2	(5.4)	22.9	(5.7)	*0.323*	13.6	(10.1)	14.7	(11.2)	*0.652*
Positive screening not confirmed, n (%)	50	(22.6)	28	(32.2)		18	(8.1)	17	(19.5)	
Mild dementia, n (%)	115	(52.1)	42	(48.3)		58	(26.2)	21	(24.1)	
Moderate dementia, n (%)	50	(22.6)	14	(16.1)	*0.275*	64	(29.0)	19	(21.8)	***0.047***
Severe dementia, n (%)	6	(2.7)	3	(3.5)		81	(36.7)	30	(34.5)	
Depression, mean (SD)	3.1	(2.4)	2.7	(1.8)	*0.176*	3.3	(2.2)	3.8	(2.9)	*0.154*
Functional impairment (BADL), mean (SD)	**3.6**	**(2.5)**	**2.7**	**(1.8)**	***0.006***	6.2	(3.1)	5.1	(3.1)	***0.005***
Number of ICD-10 diagnoses [b], mean (SD)	13.9	(8.1)	14.2	(6.8)	*0.778*	18.1	(10.7)	19.7	(9.2)	*0.745*
Charlson comorbidity ccore, mean (SD)	3.5	(2.2)	3.3	(2.3)	*0.522*	4.1	(2.6)	4.2	(2.6)	*0.697*
No, n (%)	6	(2.7)	4	(4.6)		3	(1.4)	1	(1.2)	
Low, n (%)	35	(15.8)	19	(21.8		25	(11.3)	13	(14.9)	
High, n (%)	81	(36.7)	29	(33.3	*0.483*	75	(33.9)	28	(32.2)	*0.855*
Very High, n (%)	99	(44.8)	35	(40.2		118	(53.4)	45	(51.7)	
Number of drugs taken[c], mean (SD)	6.5	(3.2)	6.4	(2.7)	*0.305*	8.0	(3.3)	7.6	(3.2)	*0.402*

Abbreviations: cDCM*,* collaborative dementia care management; MMSE, Mini Mental Status Examination; GDS, Geriatric Depression Score; BADL, Bayer Activities of Daily Living; SD, standard deviation.

Footnotes:[a] bold numbers indicate a statistically significant difference between groups based on generalized linear (metric variables) or logistic regression models (categorical variables) with random intercepts for the general practitioner (metric variables), representing the cluster.; [b] Number of ICD-10 diagnoses recorded in the medical record of treating general practitioners.; [c] Number of regular drugs taken based on cabinet review at patients' homes.

© 2024 Michalowsky B et al. *JAMA Network Open*.

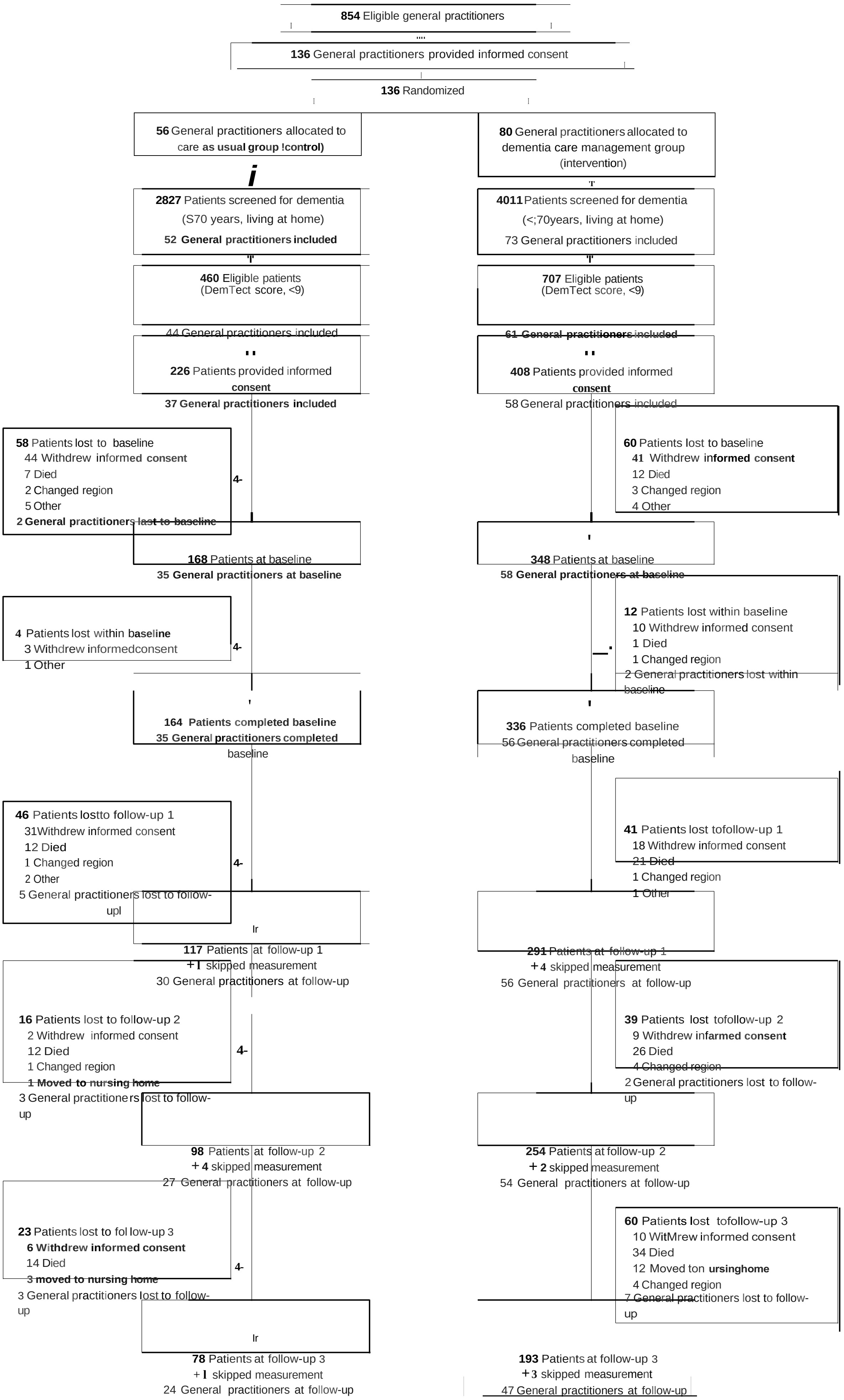

© 2024 Michalowsky B et al. *JAMA Network Open*.

eFigure 1. Flow Chart of the DelpHi-MV trial

© 2024 Michalowsky B et al. *JAMA Network Open*.

eFigure 2. Cost-effectiveness acceptability curves of the scenario analyses

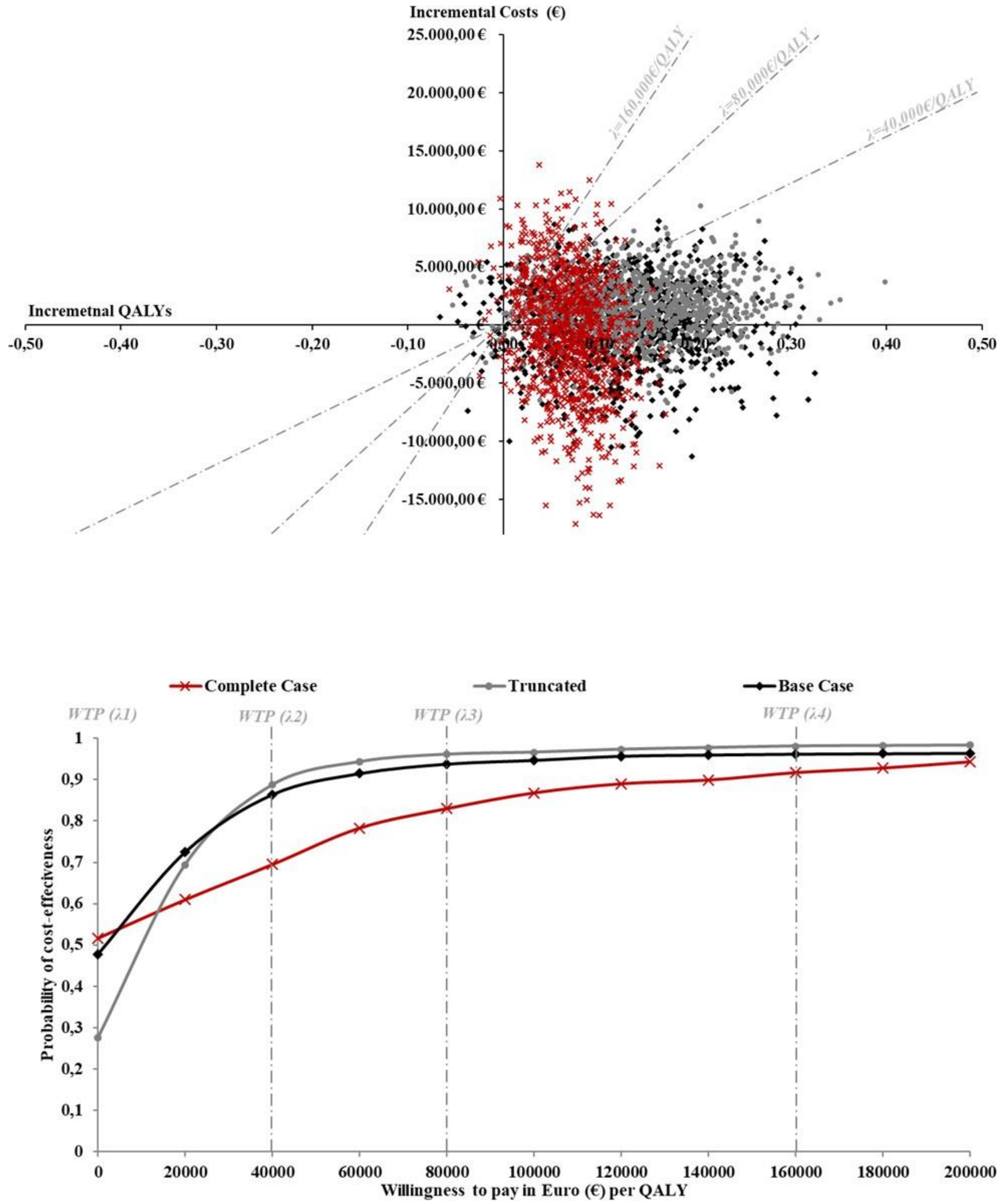

Footnote: Estimates based on regression analyses of incremental costs and effects within 1,000 bootstrap sample replications of the initial sample stratified for intervention and control group. Pain explanation of graphs: The figure above illustrates the relationship between costs and Quality-adjusted Life Years (QALYs) of collaborative dementia care management compared to usual care. Each point represents for several resamples the incremental cost and the incremental QALYs of the intervention compared to usual care, demonstrating whether the intervention is more effective and less costly (southeast quadrant), more effective but more costly (north-east quadrant), less effective and less costly (south-west quadrant), or less effective but more costly (north-west quadrant). The figure below illustrates the probability that the collaborative dementia care management is cost-effective compared to usual care at different willingness-to-pay thresholds. For example, if society is willing to pay a certain amount per Quality-adjusted Life Years (QALY), the curve indicates the likelihood that the intervention is cost-effective at that threshold. Higher curves indicate a higher probability of cost-effectiveness at various thresholds, helping decision-makers understand the uncertainty around the cost-effectiveness of the intervention.

Abbreviations: QALYs, quality-adjusted life years; WTP (l), willingness-to-pay threshold.

© 2024 Michalowsky B et al. *JAMA Network Open*.

eFigure 3. Cost-effectiveness plane and cost-effectiveness acceptability curves of the collaborative dementia care management versus care as usual after three years shown separately for patients living alone and those not living alone

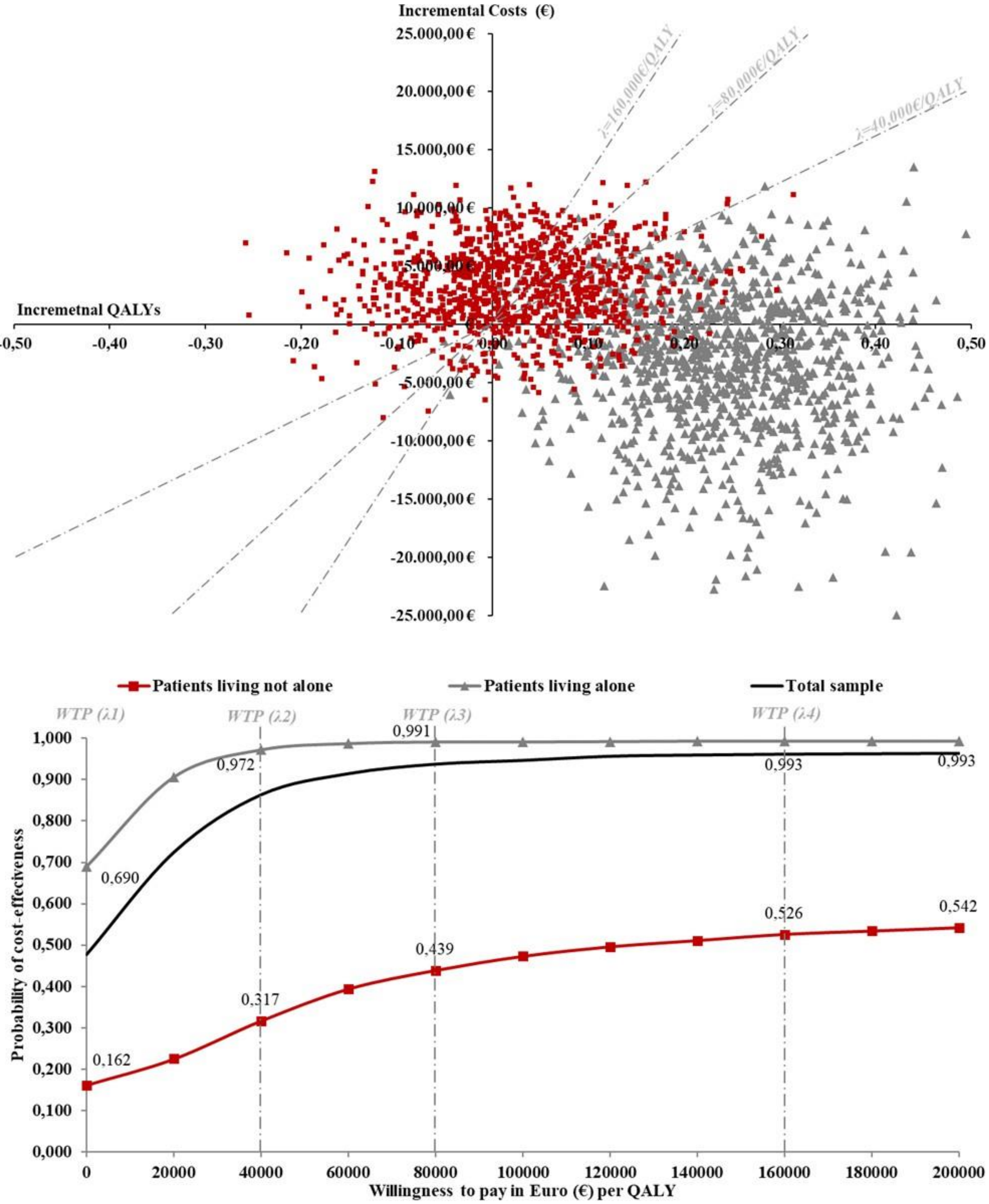

Footnote: Estimates based on regression analyses of incremental costs and effects within 1,000 bootstrap sample replications of the initial sample stratified for intervention and control group. Pain explanation of graphs: The figure above illustrates the relationship between costs and Quality-adjusted Life Years (QALYs) of collaborative dementia care management compared to usual care. Each point represents for several resamples the incremental cost and the incremental QALYs of the intervention compared to usual care, demonstrating whether the intervention is more effective and less costly (southeast quadrant), more effective but more costly (north-east quadrant), less effective and less costly (south-west quadrant), or less effective but more costly (north-west quadrant). The figure below illustrates the probability that the collaborative dementia care management is cost-effective compared to usual care at different willingness-to-pay thresholds. For example, if society is willing to pay a certain amount per Quality-adjusted Life Years (QALY), the curve indicates the likelihood that the intervention is cost-effective at that threshold. Higher curves indicate a higher probability of cost-effectiveness at various thresholds, helping decision-makers understand the uncertainty around the cost-effectiveness of the intervention.
Abbreviations: QALYs, quality-adjusted life years; WTP (l), willingness-to-pay threshold.

© 2024 Michalowsky B et al. *JAMA Network Open*.

eAppendix 1. Methods used to handle missing data and used STATA code

Missing data analyses revealed that in the DelpHi-MV-trial, patients who dropped out were more likely to be in the control group, to have higher comorbidity burden and to not have a participating caregiver. To handle missing data, we used multiple Imputation by Chained Equations (MICE) [44,70,71]. Mechanism of Multiple Imputations is described in detail by Little & Rubin [72]Mechanism of Multiple Imputations is described in detail by Little & Rubin [72] Mechanism of Multiple Imputations is described in detail by Little & Rubin [72].

Using MICE, we specify one imputation model for each variable. For resource utilization we used a Poisson regression and for health utilities a linear regression. We further adjusted each model for age, sex, living situation (alone vs. not alone) and comorbidity (number of ICD-10 diagnoses). For the imputation of missing utility values at the first and second follow-up, we further adjusted for baseline value and first follow-up value, respectively. Due to the fact that health resource utilization was assessed retrospective for a period of 12 months, we only imputed missing values at the first and second follow-up and not for baseline. Furthermore, models used solely to impute missing values at the second follow-up were adjusted for values observed or imputed at first follow-up. This was due to the fact that an adjustment for baseline utilization would consider the care situation up to 12 months prior to starting the intervention.

An imputation model for the intervention and control group together would only recognize differential means by the study group but not a differential covariance structure. Therefore, MICE were implemented separately by randomization treatment allocation for all missing values of the first and second follow-ups. Missing values of the baseline were imputed without any stratification for study groups [44].

Missing resource utilization was imputed on an item level, adding 50 additional data sets for each missing variable. In patients where only one question of the SF-12 was missing, we used a simple median imputation. If more than one value was missing, we imputed health utility scores on the item level, adding 50 additional data sets for each missing variable. Estimates obtained from each imputed data set were combined using Rubin's Rule [72] to generate an overall mean estimate together with its standard error [73]. The used STATA code was as follows:

Baseline imputation of health utilities (Q_{i0}):

mi set flong
mi register imputed Q_0
mi xtset $cluster_j$
mi impute chained (regress) Q_{i0}= var_{gender} var_{age} $var_{living\_situation}$ $var_{comorbidity}$, add(50)
egen Q_{i0_mean}= mean(Q_{i0}) by(var_{pat_id})
replace Q_{i0}= Q_{i0_mean} if Q_{i0}==.
mi unregister Q_0

Follow up 1 imputation of health utilities (Q_{i1}) and all health resources (RU_{i1}):

mi set flong
mi register imputed Q_1
mi xtset $cluster_j$
mi impute chained (regress) Q_{i1} = $var_{baseline}$ var_{gender} var_{age} $var_{living\_situation}$ $var_{comorbidity}$ if d==[0;1], add(50)
egen Q_{i1_mean}= mean(Q_{i1}) if d==[0;1], by(var_{pat_id})
replace Q_{i1}= Q_{i1_mean} if Q_{i1}==. & d==[0;1]
mi unregister Q_1

mi register imputed RU_1

© 2024 Michalowsky B et al. *JAMA Network Open*.

mi xtset $cluster_j$
mi impute chained (poisson) RU_{i1}= var_{gender} var_{age} $var_{living\_situation}$ $var_{comorbidity}$ if d==[0;1], add(50)
egen RU_{i1_mean}= mean(RU_{i1}) if d==[0;1], by(var_{pat_id})
replace RU_{i1}= RU_{i1_mean} if RU_{i1}==. & d==[0;1]
mi unregister RU_1

Follow up 2 imputation of health utilities (Q_{i2}) and all health resources (RU_{i2}):
mi register imputed Q_2
mi xtset $cluster_j$
mi impute chained (regress) Q_{i2}= $var_{baseline}$ $var_{follow\text{-}up1}$ var_{gender} var_{age} $var_{living\_situation}$ $var_{comorbidity}$ if d==[0;1], add(50)
egen Qi_{2_mean}= mean(Qi_2) if d==[0;1], by(var_{pat_id})
replace Qi_2= Qi_{2_mean} if Qi_2==. & d==[0;1]
mi unregister Q_2
mi register imputed RU_2
mi xtset $cluster_j$
mi impute chained (poisson) RU_{i2}= $var_{follow\text{-}up1}$ var_{gender} var_{age} $var_{living\_situation}$ $var_{comorbidity}$ if d==[0;1], add(50)
egen RU_{i2_mean}= mean(RUi_2) if d==[0;1], by(var_{pat_id})
replace RU_{i2}= RU_{i2_mean} if RU_2==. & d==[0;1]
mi unregister RU_2

Follow up 3 imputation of health utilities (Q_{i2}) and all health resources (RU_{i2}):
mi register imputed Q_3
mi xtset $cluster_j$
mi impute chained (regress) Q_{i3}= $var_{baseline}$ $var_{follow\text{-}up1}$ $var_{follow\text{-}up2}$ var_{gender} var_{age} $var_{living\_situation}$ $var_{comorbidity}$ if d==[0;1], add(50)
egen Qi_{3_mean}= mean(Qi_3) if d==[0;1], by(var_{pat_id})
replace Qi_3= Qi_{3_mean} if Qi_3==. & d==[0;1]
mi unregister Q_3
mi register imputed RU_3
mi xtset $cluster_j$
mi impute chained (poisson) RU_{i3}= $var_{follow\text{-}up1}$ $var_{follow\text{-}up2}$ var_{gender} var_{age} $var_{living\_situation}$ $var_{comorbidity}$ if d==[0;1], add(50)
egen RU_{i3_mean}= mean(RUi_3) if d==[0;1], by(var_{pat_id})
replace RU_{i3}= RU_{i3_mean} if RU_3==. & d==[0;1]
mi unregister RU_3

$\boldsymbol{Q_{itj}}$ $= Health\ utility\ of\ patient\ \boldsymbol{i}\ at\ point\ \boldsymbol{t}\ in\ cluster\ \boldsymbol{j}$

$\boldsymbol{RU_{itj}}$ $= Health\ utilization\ of\ patient\ \boldsymbol{i}\ at\ point\ \boldsymbol{t}\ in\ cluster\ \boldsymbol{j}$

$\boldsymbol{Q_{itj\_mean}}$ $= Mean\ estimated\ health\ utility\ of\ patient\ \boldsymbol{i}\ at\ point\ \boldsymbol{t}\ in\ cluster\ \boldsymbol{j}$

$\boldsymbol{RU_{itj\_mean}}$ $= Mean\ estimated\ health\ utility\ of\ patient\ \boldsymbol{i}\ at\ point\ \boldsymbol{t}\ in\ cluster\ \boldsymbol{j}$

$\boldsymbol{d_{ij}}$ $= Treatment\ dummy\ variable\ (0 = control\ group;\ 1 = intervention)$

© 2024 Michalowsky B et al. *JAMA Network Open*.

eAppendix 2. Intraclass correlation (ICC) and design effects (deff) for each outcome and time point

NPI

- Baseline
  - ICC=0.48; **ICC > 0.3** | *deff* = -0.056; ***deff* < 2,00**
- Follow-up 1
  - ICC=0.00; **ICC < 0.05** | *deff* = 1; ***deff* < 2,00**
- Follow-up 2
  - ICC=0.17; **0,05 < ICC < 0.3** | *deff* = 0.626; ***deff* < 2,00**
- Follow-up 3
  - ICC=0.32; **ICC > 0.3** | *deff* = 0.296; ***deff* < 2,00**

BIZA

- Baseline
  - ICC= 0.00; **ICC < 0.05** | *deff* = 1; ***deff* < 2,00**
- Follow-up 1
  - ICC=0.08; **0,05 < ICC < 0.3** | *deff* = 0.824; ***deff* < 2,00**
- Follow-up 2
  - ICC=0.06; **0,05 < ICC < 0.3** | *deff* = 0.868; ***deff* < 2,00**
- Follow-up 3
  - ICC=0.16; **0,05 < ICC < 0.3** | *deff* = 0.648; ***deff* < 2,00**

BADL

- Baseline
  - ICC=0.03; **ICC < 0.05** | *deff* = 0.91; ***deff* < 2,00**
- Follow-up 1
  - ICC=0.07; **0,05 < ICC < 0.3** | *deff* = 0.79; ***deff* < 2,00**
- Follow-up 2
  - ICC=0.06; **0,05 < ICC < 0.3** | *deff* = 0.82; ***deff* < 2,00**
- Follow-up 3
  - ICC= 0.08; **0,05 < ICC < 0.3** | *deff* = 0.76; ***deff* < 2,00**

SF12 mental

- Baseline
  - ICC=0.10; **0,05 < ICC < 0.3** | *deff* = 0.7; ***deff* < 2,00**
- Follow-up 1
  - ICC=0.11; **0,05 < ICC < 0.3** | *deff* = 0.67; ***deff* < 2,00**
- Follow-up 2
  - ICC=0.03; **ICC < 0.05** | *deff* = 0.91; ***deff* < 2,00**
- Follow-up 3
  - ICC=0.14; **0,05 < ICC < 0.3** | *deff* = 0.58; ***deff* < 2,00**

SF12 Physical

- Baseline
  - ICC=0.00; **ICC < 0.05** | *deff* = 1; ***deff* < 2,00**
- Follow-up 1
  - ICC=0.00; **ICC < 0.05** | *deff* = 1; ***deff* < 2,00**
- Follow-up 2
  - ICC=0.03; **ICC < 0.05** | *deff* = 0.91; ***deff* < 2,00**
- Follow-up 3
  - ICC=0.04; **ICC < 0.05** | *deff* = 0.88 ; ***deff* < 2,00**

© 2024 Michalowsky B et al. *JAMA Network Open*.

eAppendix 3. Standardized measure of effect size (Cohens f^2) for each outcome and time point

NPI

- Follow-up 1
  - Cohens f^2 = -0.005; **$|f^2| < 0.02$**
- Follow-up 2
  - Cohens f^2 = -0.005; **$|f^2| < 0.02$**
- Follow-up 3
  - Cohens f^2 = 0.03; **$0.02 < |f^2| < 0.15$**

BIZA

- Follow-up 1
  - Cohens f^2 = -0.003; **$|f^2| < 0.02$**
- Follow-up 2
  - Cohens f^2 = -0.005; **$|f^2| < 0.02$**
- Follow-up 3
  - Cohens f^2 = 0.008; **$|f^2| < 0.02$**

BADL

- Follow-up 1
  - Cohens f^2 =0.006; **$|f^2| < 0.02$**
- Follow-up 2
  - Cohens f^2 =0.003; **$|f^2| < 0.02$**
- Follow-up 3
  - Cohens f^2 =-0.002; **$|f^2| < 0.02$**

SF12 Mental

- Follow-up 1
  - Cohens f^2 =0.006; **$|f^2| < 0.02$**
- Follow-up 2
  - Cohens f^2 =-0.002; **$|f^2| < 0.02$**
- Follow-up 3
  - Cohens f^2 =-0.0032; **$|f^2| < 0.02$**

SF12 Physical

- Follow-up 1
  - Cohens f^2 =-0.002; **$|f^2| < 0.02$**
- Follow-up 2
  - Cohens f^2 =-0.002; **$|f^2| < 0.02$**
- Follow-up 3
  - Cohens f^2 =-0.002; **$|f^2| < 0.02$**

© 2024 Michalowsky B et al. *JAMA Network Open*.

eAppendix 4. Calculation of Utility Values and Quality Adjusted Life Years

$$Q_i(t) = \begin{cases} Q_{i0}: & 0 \le t \\ Q_{ih} + \dfrac{(Q_{i,h+1} - Q_{ih})\,(t - t_{ih})}{t_{i,h+1} - t_{ih}}: & t_{ih} \le t \le t_{i,h+1} \\ Q_{im_i}: & t_{im_i} \le t < X_i \\ 0: & t \ge X_i \end{cases}$$

$Q_i(t)$ $= utility\ value\ of\ subject\ \boldsymbol{i}\ at\ point\ \boldsymbol{t}$

Q_{i0} $= utility\ value\ of\ subject\ \boldsymbol{i}\ at\ baseline$

Q_{ih} $= utility\ value\ of\ subject\ \boldsymbol{i}\ at\ assessment\ point\ \boldsymbol{h}$

Q_{im_i} $= utility\ value\ of\ subject\ \boldsymbol{i}\ at\ the\ last\ point\ of\ assessment\ \boldsymbol{m}$

X_i $= duration\ of\ interest\ i.e.\ length\ of\ the\ trial\ or\ death$

$$q_i(t) = \begin{cases} (t - t_{i0})\left(\dfrac{Q_i(t) + Q_{i0}}{2}\right): & t_{i0} \le t \le t_{i1} \\ (t_{i1} - t_{i0})\left(\dfrac{Q_{i0} + Q_{i1}}{2}\right) + (t - t_{i1})\left(\dfrac{Q_i(t) + Q_{i1}}{2}\right): & t_{i1} \le t \le t_{i2} \\ (t_{i1} - t_{i0})\left(\dfrac{Q_{i0} + Q_{i1}}{2}\right) + (t_{i2} - t_{i1})\left(\dfrac{Q_{i2} + Q_{i1}}{2}\right) + (t - t_{i2})Q_{i2}: & t_{i2} \le t \le X_i \\ (t_{i1} - t_{i0})\left(\dfrac{Q_{i0} + Q_{i1}}{2}\right) + (t_{i2} - t_{i1})\left(\dfrac{Q_{i2} + Q_{i1}}{2}\right) + (X_i - t_{i2})Q_{i2}: & t > X_i \end{cases}$$

$q_i(t)$ $= QALY\ of\ patient\ \boldsymbol{i}\ at\ point\ \boldsymbol{t}$

Q_{i1} $= utility\ value\ of\ subject\ \boldsymbol{i}\ at\ FU1$

Q_{i2} $= utility\ value\ of\ subject\ \boldsymbol{i}\ at\ FU2$

$$q_{ij}(t) = \alpha + \left(\sum_{k=1}^{p} \beta_k x_{ijk}\right) + \Delta_e d_{ij} + \gamma Q_{ij}(0) + u_j + \varepsilon_{ij}$$

$q_{ij}(t)$ $= QALY\ of\ patient\ \boldsymbol{i}\ in\ cluster\ \boldsymbol{j}\ at\ point\ \boldsymbol{t}$

α $= Intercept$

β_k $= Coefficient\ of\ the\ covariate\ \boldsymbol{k}$

© 2024 Michalowsky B et al. *JAMA Network Open*.

$\boldsymbol{x_{ijk}} = \boldsymbol{k}^{th}\ covariate\ for\ patient\ \boldsymbol{i}\ in\ cluster\ \boldsymbol{j}$

$\Delta_{\boldsymbol{e}} = Estimate\ of\ the\ between-arm\ difference\ in\ mean\ QALY$

© 2024 Michalowsky B et al. *JAMA Network Open*.

$\boldsymbol{d_{ij}}$ $= Treatment\ dummy\ variable\ (0\ = control\ group;\ \ = DelpHi - MV)\ in\ cluster\ \boldsymbol{j}$

$\boldsymbol{\gamma}$ $= Coefficient\ of\ baseline\ utility\ value$

$\boldsymbol{Q_{ij}(0)}$ $= \ Baseline\ utility\ value\ of\ patient\ \boldsymbol{i}\ in\ cluster\ \boldsymbol{j}$

$\boldsymbol{u_j}$ $= Random\ effect\ for\ the\ \boldsymbol{j}^{th}\ cluster$

$\boldsymbol{\varepsilon_{ij}}$ $= Level\ one\ residual\ for\ the\ \boldsymbol{i}^{th}\ patient\ in\ the\ \boldsymbol{j}^{th}\ cluster$

$$ICER = \frac{(\bar{c}_{I1} - \bar{c}_{C1}) + [(\bar{c}_{I2} \times r) - (\bar{c}_{C2} \times r)] + [(\bar{c}_{I3} \times r) - (\bar{c}_{C3} \times r^2)]}{(\bar{q}_{I1} - \bar{q}_{C1}) + [(\bar{q}_{I2} \times r) - (\bar{q}_{C2} \times r)] + [(\bar{q}_{I3} \times r) - (\bar{q}_{C3} \times r^2)]} = \frac{(\bar{C}_I - \bar{C}_C)}{(\bar{E}_I - \bar{E}_C)} = \frac{\Delta\bar{C}}{\Delta\bar{E}}$$

$\boldsymbol{q_{It}}$ $= Mean\ QALY\ of\ intervention\ group\ at\ point\ \boldsymbol{t}$

$\boldsymbol{q_{Ct}}$ $= Mean\ QALY\ of\ control\ group\ at\ point\ \boldsymbol{t}$

$\boldsymbol{c_{It}}$ $= Mean\ cost\ of\ intervention\ group\ at\ point\ \boldsymbol{t}$

$\boldsymbol{c_{Ct}}$ $= Mean\ cost\ of\ c\ group\ at\ point\ \boldsymbol{t}$

$\boldsymbol{\bar{E}_I}$ $= Mean\ effect\ of\ the\ intervention\ group$

$\boldsymbol{\bar{E}_C}$ $= Mean\ effect\ of\ the\ control\ group$

$\boldsymbol{\bar{C}_I}$ $= Mean\ cost\ of\ the\ intervention\ group$

$\boldsymbol{\bar{C}_C}$ $= Mean\ cost\ of\ the\ control\ group$

$\Delta\boldsymbol{\bar{C}} = Incremental\ cost$

$\Delta\boldsymbol{\bar{E}} = Incremental\ effect$

$\boldsymbol{r}$ $= dicouting\ rate$

$$\overline{\boldsymbol{NB}} = \Delta\boldsymbol{\bar{E}} \times \boldsymbol{\lambda} - \Delta\boldsymbol{\bar{C}} > \boldsymbol{0}$$

λ $= Willingness - to - pay\ threshold$

© 2024 Michalowsky B et al. *JAMA Network Open*.
