# Supplementary material for: Clinical Outcomes and Cost-Effectiveness of Collaborative Dementia Care: A Secondary Analysis of a Cluster Randomized Clinical Trial
Source: JAMA Netw Open. 2024 Jul 5;7(7):e2419282. doi: 10.1001/jamanetworkopen.2024.19282 (PMC11227088; doi:10.1001/jamanetworkopen.2024.19282)
Supplement: Supplement 3. — Data Sharing Statement [file jamanetwopen-e2419282-s003.pdf]

Data Sharing Statement

Michalowsky. Clinical Outcomes and Cost-Effectiveness of Collaborative Dementia Care.
JAMA Netw Open. Published July 05, 2024. doi:10.1001/jamanetworkopen.2024.19282

Data
Data available: No

Additional Information
Explanation for why data not available: Data can be made available after request.
